# Supplementary material for: CD20 tails interact with the 14-3-3/GEF-H1 complex and microtubule network upon PKCδ phosphorylation
Source: EMBO J. 2026 Apr 17;45(11):3859–79. doi: 10.1038/s44318-026-00781-5 (PMC13226681; doi:10.1038/s44318-026-00781-5)
Supplement: Supplementary file 1 — Appfendix [file 44318_2026_781_MOESM1_ESM.pdf]

Appendix for:

CD20 tails interact with the 14-3-3/GEF-H1 complex and microtubule network upon PKCδ phosphorylation

Table of Contents:

Appendix Figure S1 Page 2

Appendix Figure S2 Page 3

Appendix Figure S3 Page 4

Appendix Figure S4 Page 5

Appendix Figure S5 Page 6

Appendix Figure S6 Page 7

Appendix Figure S1

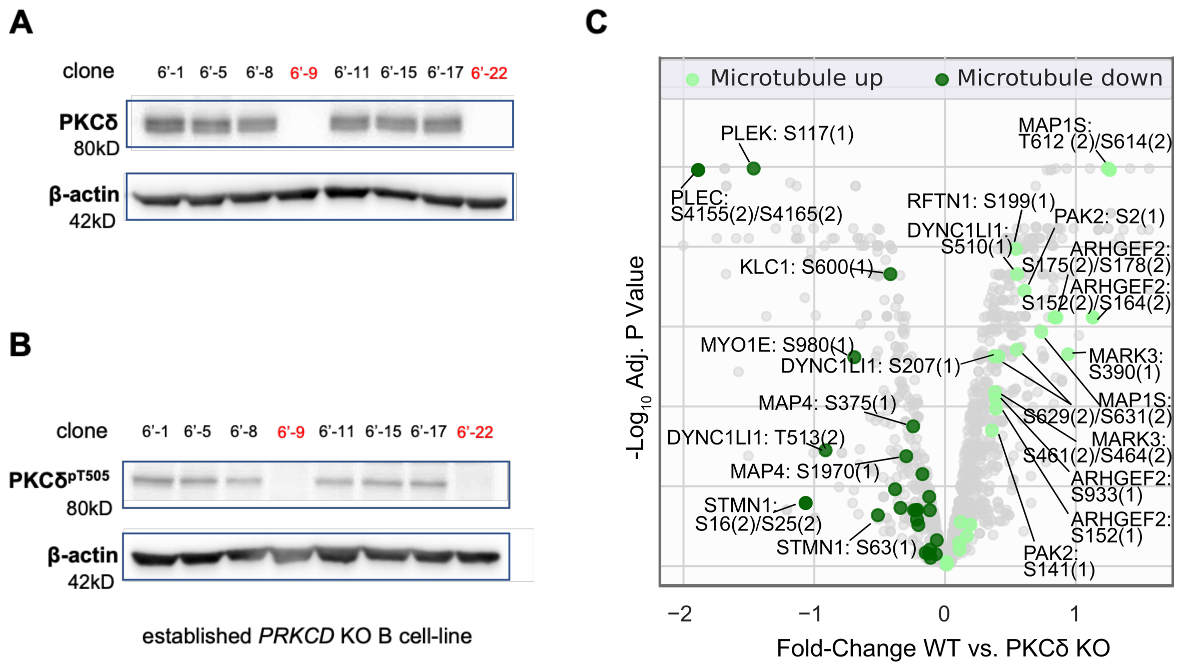

Appendix figure S1:

Western blot analysis of PKCδ after CRISPR-Cas9 mediated PKCδ KO. Lysates were stained for PKCδ **A**. or PKCδ phospho-threonine 505 (PKCδ pT505) **B**. Clones 6'-9 and 6'-22 were selected for further PKCδ KO studies. **C**. Volcano blot indicating the changes of the constitutive serine or threonine protein phosphosites of PKCδ KO compared to Ramos cells. On the X-axis the plot depicts log2 fold change of phosphorylated serine or threonine residues in the whole proteome of the two cell lines. The Y-axis shows the log10 FDR adjusted p-value. Decreased phosphorylation of serine residues of the MT pathway are shown in dark green, increase in light green. The phosphorylated serine residues of MT proteins are indicated (multiplicities in brackets). The complete raw dataset is shown in Dataset EV1 and via the PRIDE repository (PXD063667).

Appendix Figure S2

A

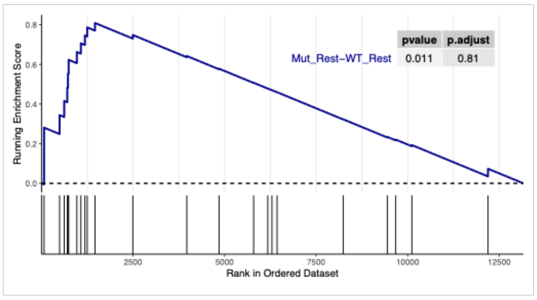

GOBP_REGULATION_OF_MICROTUBULE_DEPOLYMERIZATION_Mut_Rest-WT_Rest_plot

B

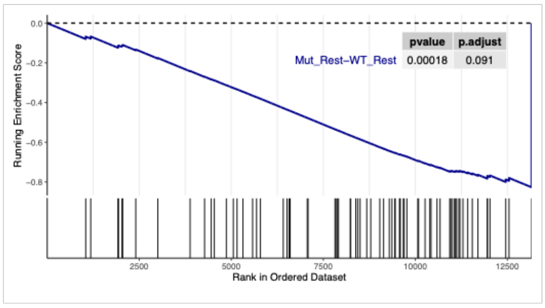

C

GOBP_REGULATION_OF_MICROTUBULE_DEPOLYMERIZATION, UP in PKCδ KO, rest<br>Any process that modulates the frequency, rate or extent of microtubule depolymerization.
APC2, MAP6D1, MID1, MAP1S, CAMSAP2, CKAP2, TTBK2, APC, SPAST, MID1IP1, TAOK1

GOCC_CORTICAL_CYTOSKELETON, DOWN in PKCδ KO, rest<br>The portion of the microtubule cytoskeleton that lies just beneath the plasma membrane.
SPTBN1, RIMS3, RIMS1

Appendix figure S2:

Gene Set Enrichment Analysis (GSEA) of genes associated with microtubule destabilization and cytoskeletal organization.

A. The plot illustrates the enrichment of genes related to positive regulation of microtubule disassembly in PKCδ knockout (KO) cells compared to wild-type (WT) Ramos cells. The x-axis represents the position of genes from the specified gene set within the ranked list derived from the comparison between PKCδ-KO and Ramos WT cells. The y-axis depicts the running enrichment score (ES). Vertical black lines indicate the positions of genes associated with microtubule depolymerization within the ranked list (p-value = 0.011). **B.** The plot shows a decrease in genes essential for the localization of the cortical cytoskeleton (the cytoskeletal region just beneath the plasma membrane) in PKCδ-KO cells relative to Ramos WT cells (p-value = 0.00018). The peak of the blue curve represents the maximum enrichment score, indicating the point where the gene set is most significantly enriched based on the expression differences between two independent, unstimulated PKCδ-KO Ramos cell clones and Ramos WT cells. **C.** the table shows some of the regulated key genes of A (top) and B (bottom) GSEA.

Appendix Figure S3

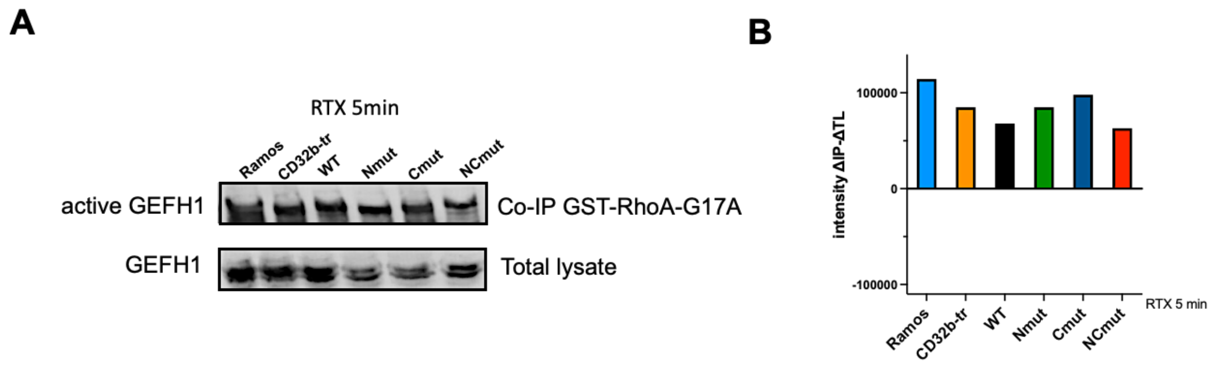

Appendix figure S3:

A. Western blot analysis of free GEF-H1 in the lysate of Ramos and CD32b-tr cells as well as reconstituted Ramos cells expressing CD20 WT, Nmut, Cmut or NCmut after activation with RTX for 5 min. The amount of GEF-H1 purified with the GST-RhoAG17A beads and in the total lysate is shown at the top and bottom, respectively. **B.** Quantification of the Western blot data with Image Studio Lite (Licor) showing the difference of signal intensity in the immunoprecipitate (IP) versus that of the total lysates (TL).

Appendix Figure S4

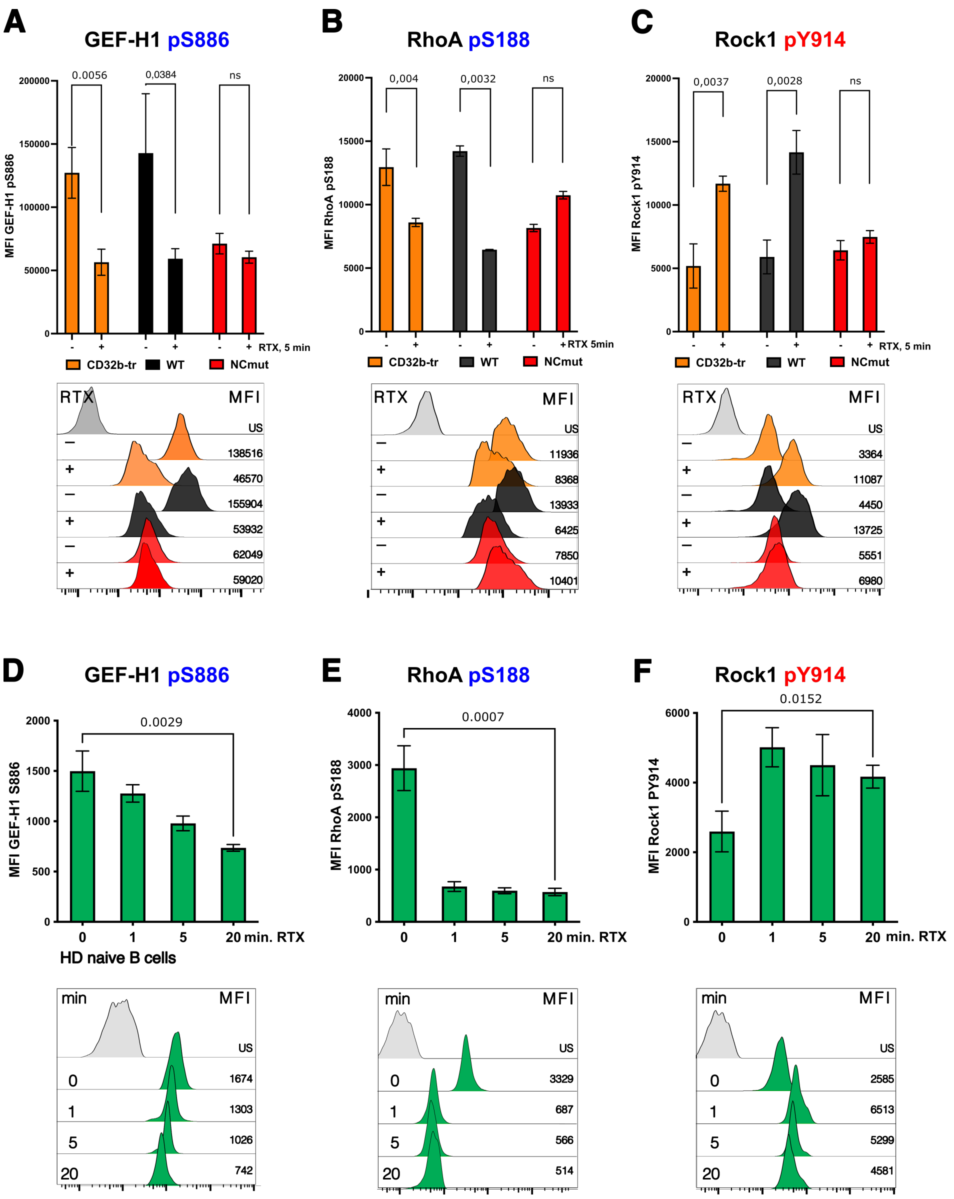

Appendix figure S4:

Intracellular phospho-flow cytometry analysis of intracellular signal proteins upon RTX treatment for 5 min compared to unstimulated CD20 WT and NCmut S>A transfectant show: **A** Loss of inhibitory GEF-H1 phosphorylation Serine^{886}, which is a prerequisite for the activation of RhoA. **B** Loss of inhibitory RhoA Serine^{188} phosphorylation, which renders RhoA active. RhoA-GTP allows the increase of **C** active Rock1, phosphorylated at Y^{914}**.**

Students t-tests calculated with PRISM 10, show significant differences between the resting and stimulated state of CD32b-tr and WT cells, but not between S>A mutants. Representative NCmut CD20 mutant is presented. Top: summary data, n=6, bottom: corresponding example flow data of A-C. **D- F** Intracellular phospho-flow of the same protein residues as in **A-C** but on HD naive B cells and as a time course after RTX stimulation for 0, 1, 5, 20 min. **D** inhibitory GEF-H1 S^{886} phosphorylation, **E** inhibitory RhoA S^{188}, and **F** activatory Rock1 Y914 phosphorylation, n=6. Significant differences between the groups were calculated with PRISM 10, one-way ANOVA. Activatory phosphorylations are depicted in red, inhibitory are shown in blue. Top: summary data, n=3, bottom: the corresponding example flow data of D-F. Mean with SD is presented.

Appendix Figure S5

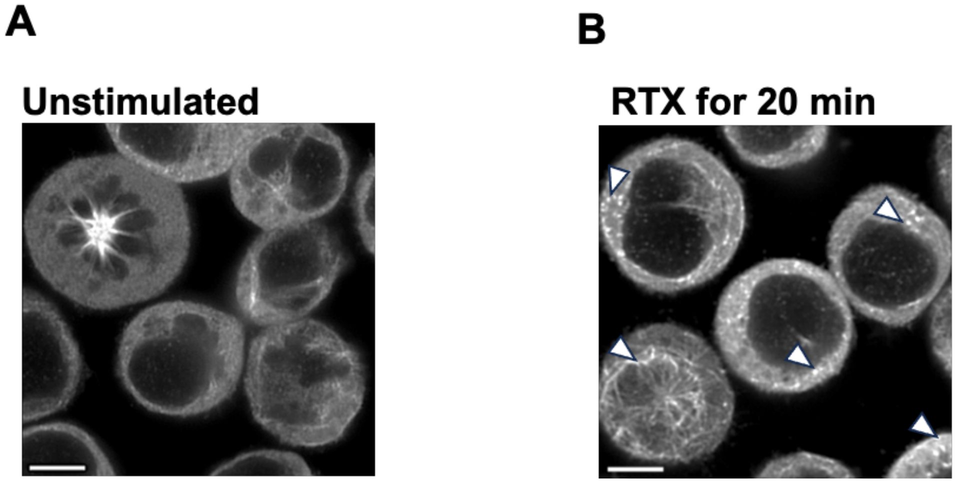

Appendix figure S5:

Airyscan confocal microscopy images of Ramos CD32b-tr cells **A.** unstimulated or **B.** incubated with RTX for 20 min. Cells were fixed and stained for α-tubulin with Alexa Fluor 555 to visualize MTs. Images present the mid-section of an Airyscan processed z-stack. White arrows in **B.** indicate the peeling of the curved protofilaments away from MT in the treated cells. Scale bar: 5μm.

Appendix Figure S6

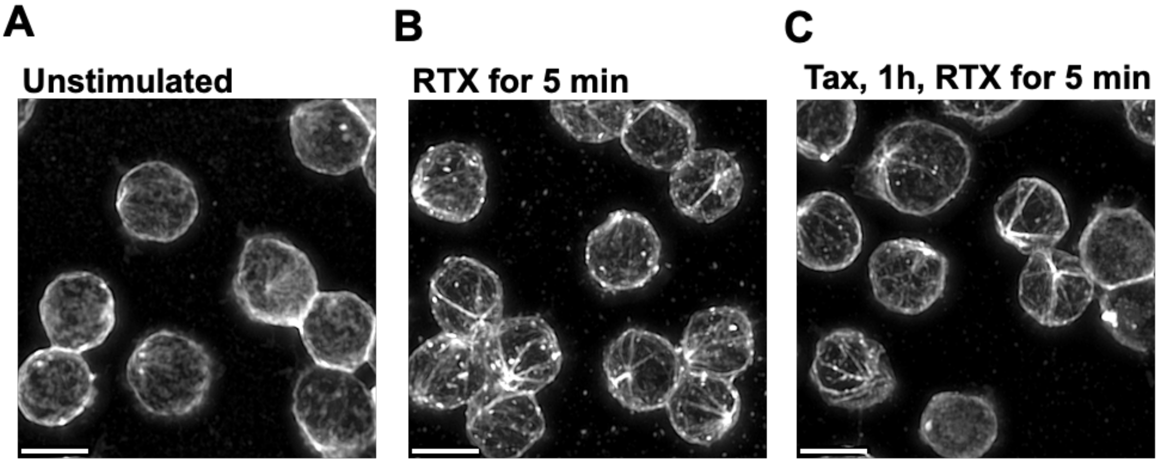

Appendix figure S6:

Maximum intensity projection of Airyscan (SR) confocal z-stacks of HD naïve B cells **A.** untreated, **B.** upon exposure to RTX for 5 min or **C**. with Taxol pretreatment for 1h before exposure to RTX for 5 min. Cells were fixed and stained for α-tubulin with Alexa Fluor 555. In **B.** background is brightened due to higher presence of dead or destroyed cells. Scale bar: 5µm.
