# Supplementary material for: CD20 tails interact with the 14-3-3/GEF-H1 complex and microtubule network upon PKCδ phosphorylation
Source: EMBO J. 2026 Apr 17;45(11):3859–79. doi: 10.1038/s44318-026-00781-5 (PMC13226681; doi:10.1038/s44318-026-00781-5)
Supplement: Supplementary file 2 — Table EV1 [file 44318_2026_781_MOESM2_ESM.docx]

**Table EV1**

**Extended Table 1**

CD20-WT geneblock

5'/5Phos/TCTACCCAATACTGTTACAGCATACAATCTCTGTTCTTGGGCATTTTGTCAGTGATGCTGATCTTTGCCTTCTTCCAGGAACTTGTAATAGCTGGCATCGTTGAGAATGAATGGAAAATAACGTGCTCCAGACCCTTTGCCTTCTTCCAGGAACTTGTAATAGCTGGCATCGTTGAGAATGAATGGAAAATAACGTGCTCCAGACCCAAATCTAACATAGTTCTCCTGTCAGCAGAAGAAAAAAAAGAACAGACTATTGAAATAAAAGAAGAAGTGGTTAAATCTAACATAGTTCTCCTGTCAGCAGAAGAAAAAAAAGAACAGACTATTGAAATAAAAGAAGAAGTGGTTGGGCTAACTGAAACATCTTCCCAACCAAAGAATGAAGAAGACATTGAAATTATTCCAATCCAAGAAGAGGAAGGGCTAACTGAAACATCTTCCCAACCAAAGAATGAAGAAGACATTGAAATTATTCCAATCCAAGAAGAGGAAGAAGAAGAAACAGAGACGAACTTTCCAGAACCTCCCCAAGATCAGGAATCCTCACCAATAGAAAATGACAGCGAAGAAGAAACAGAGACGAACTTTCCAGAACCTCCCCAAGATCAGGAATCCTCACCAATAGAAAATGACAGCTCTCCTGATTACAAGGATGACGACGATAAGTGACGCCCCCCCCCCCTAACGTTACTGGCCGAAGCCGCTTGGTCTCCTGATTACAAGGATGACGACGATAAGTGACGCCCCCCCCCCCTAACGTTACTGGCCGAAGCCGCTTGGAATAAGGCCGGTGTGCGTTTGTCTATATGTTATTTTCCACCATATTGCCGTCTTTTGGCAATAAGGCCGGTGTGCGTTTGTCTATATGTTATTTTCCACCATATTGCCGTCTTTTGGC-3'

CD20-C-term Cmut geneblock

5'/5Phos/GTTGAGAATGAATGGAAAAGAACGTGCGCCAGACCCAAAGCTAACATAGTTCTCCTGTCAGCAGAAGAAAAAAAAGAACAGACTATTGAAATAAAAGAAGAAGTGGTTGGGCTAACTGAAACATCTTCCCAACCAAAGAATGAAGAAGACATTGAAATTATTCCAATCCAAGAAGAGGAAGAAGAAGAAACAGAGACGAACTTTCCAGAACCTCCCCAAGATCAGGAATCCTCACCAATAGAAAATGACAGCTCTCCT-3'

CD20-N-term Nmut geneblock

5'/5Phos/TCCTCCATCCGCCCCGTCTCTCCCCCTTGAACCTCCTCGTTCGACCCCGCCTCGATCCTCCCTTTATCCAGCCCTCACTCCTTCTCTAGGCGCCGGAATTAGATCTCTCGAGGTTAACGAATTCATGACAACACCCAGAAATGCAGTAAATGGGACTTTCCCGGCAGAGCCAATGAAAGGCCCTATTGCTATGCAATCTGGTCCAAAACCACTCTTCAGGAGGATGGCTGCACTGGTGGGCCCCACGCAAAGCTTCTTCATGAGGGAAGCTAAGACTTTGGGGGCTGTCCAGATTATGAATGGGCTCTTCCACATTGCCCTGGGGGGTGTTGTGATGATCCCAGCAGGGATCTATGCACCCATCTGTGTGACTGTGTGGTACCCTCTCTGGGGAGGC-3’
