# Supplementary figures and images for: CD20 tails interact with the 14-3-3/GEF-H1 complex and microtubule network upon PKCδ phosphorylation
Source: EMBO J. 2026 Apr 17;45(11):3859–79. doi: 10.1038/s44318-026-00781-5 (PMC13226681; doi:10.1038/s44318-026-00781-5)

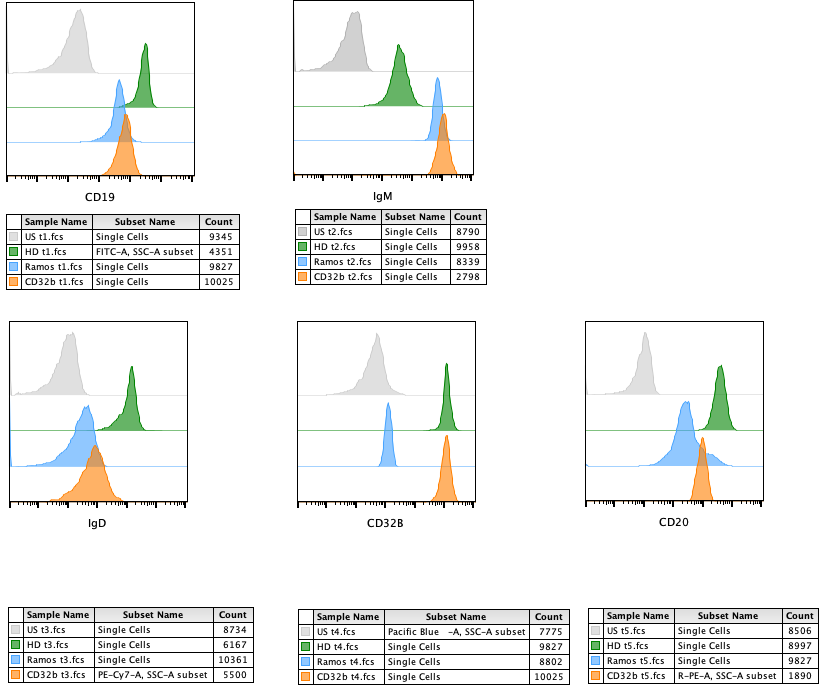

Supplement: Supplementary file 5 — Source data Fig. 1 [file 44318_2026_781_MOESM5_ESM.zip › Figure 1/A/Copy layout workspace.docx]

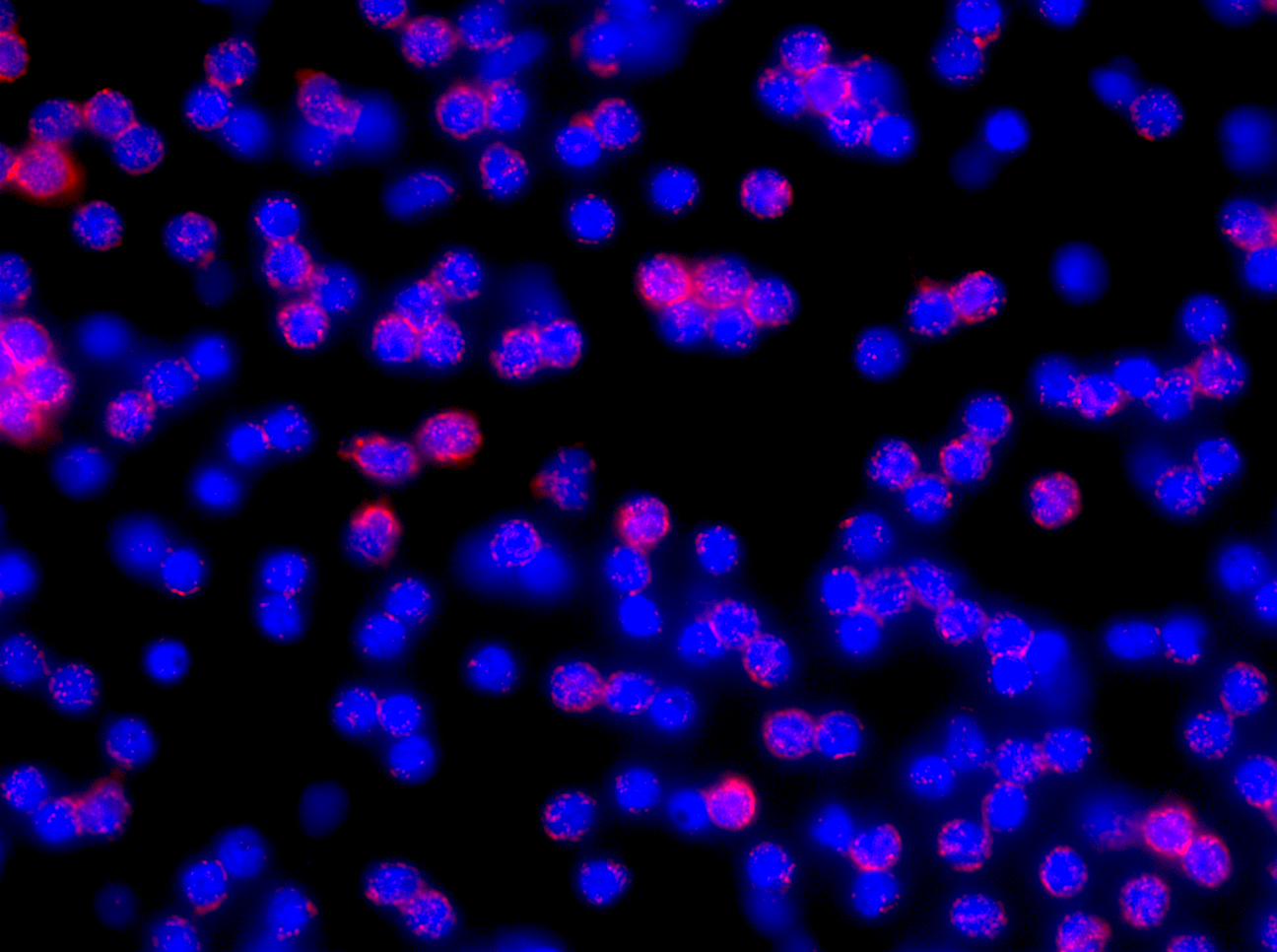

Supplement: Supplementary file 5 — Source data Fig. 1 [file 44318_2026_781_MOESM5_ESM.zip › Figure 1/D/Fig 1 D CD32B HD.tiff]

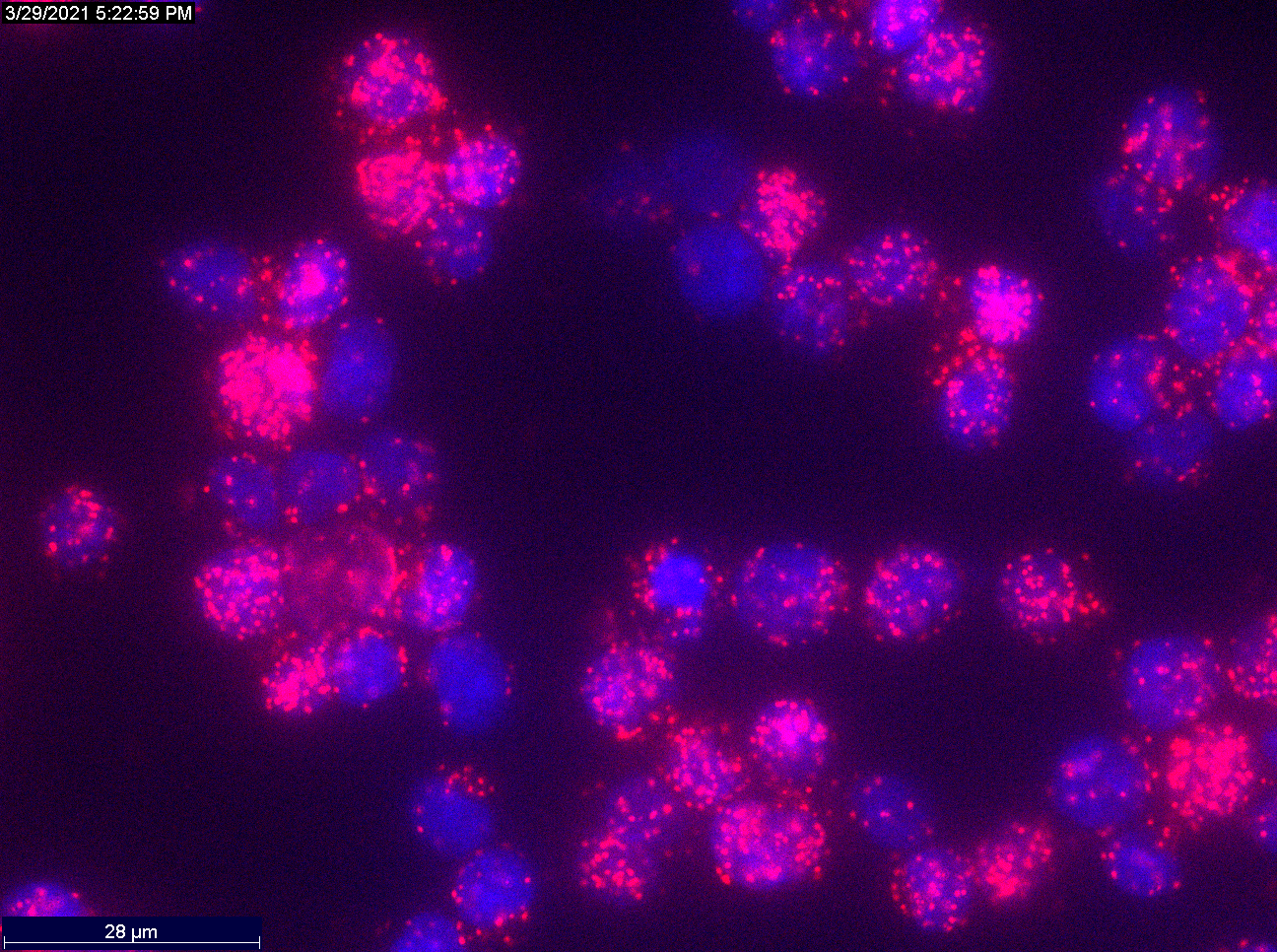

Supplement: Supplementary file 5 — Source data Fig. 1 [file 44318_2026_781_MOESM5_ESM.zip › Figure 1/D/Fig 1 D CD32B_25.tif]

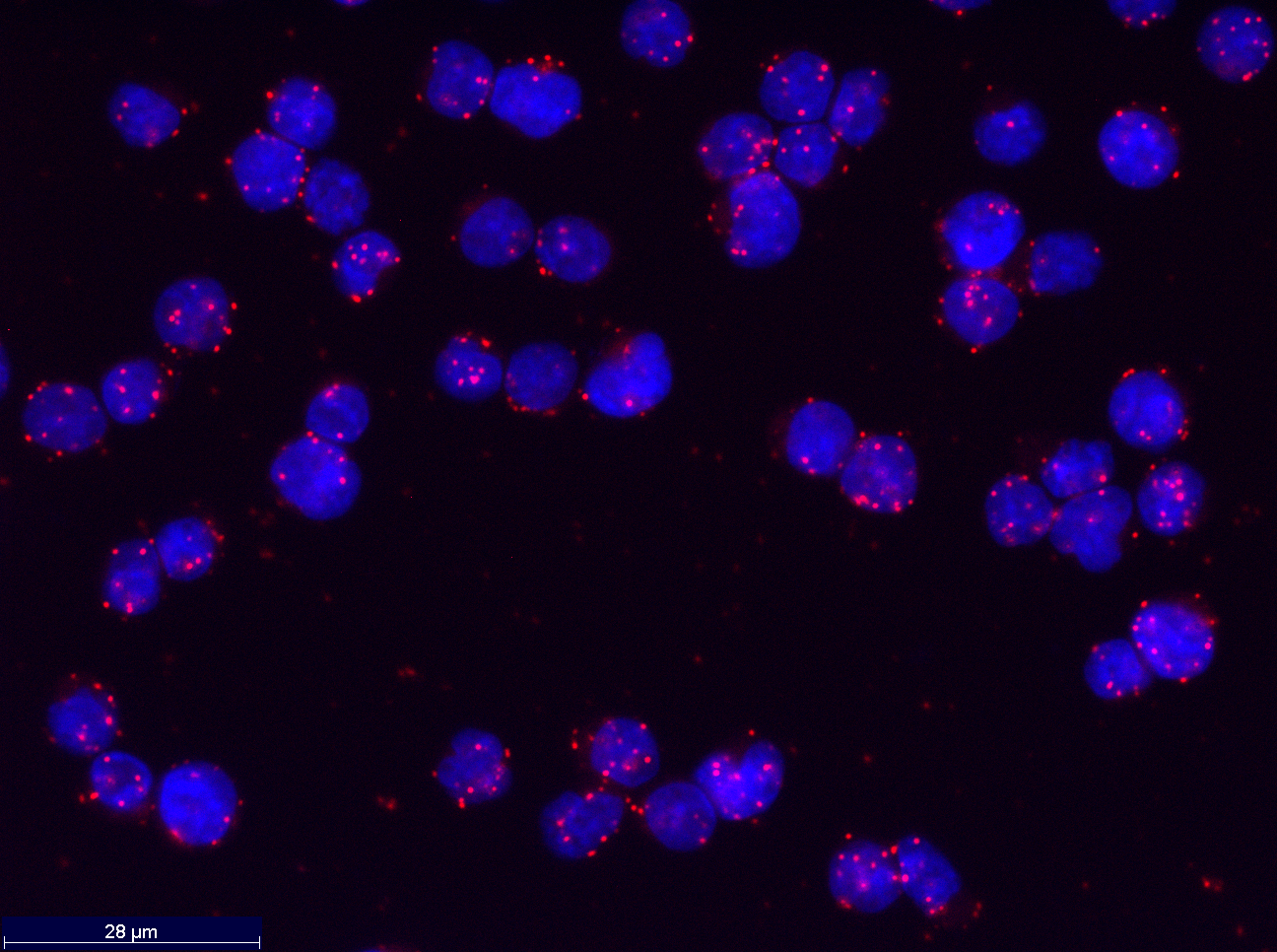

Supplement: Supplementary file 5 — Source data Fig. 1 [file 44318_2026_781_MOESM5_ESM.zip › Figure 1/D/221115 Fig 1 D Ramos paper.tiff]

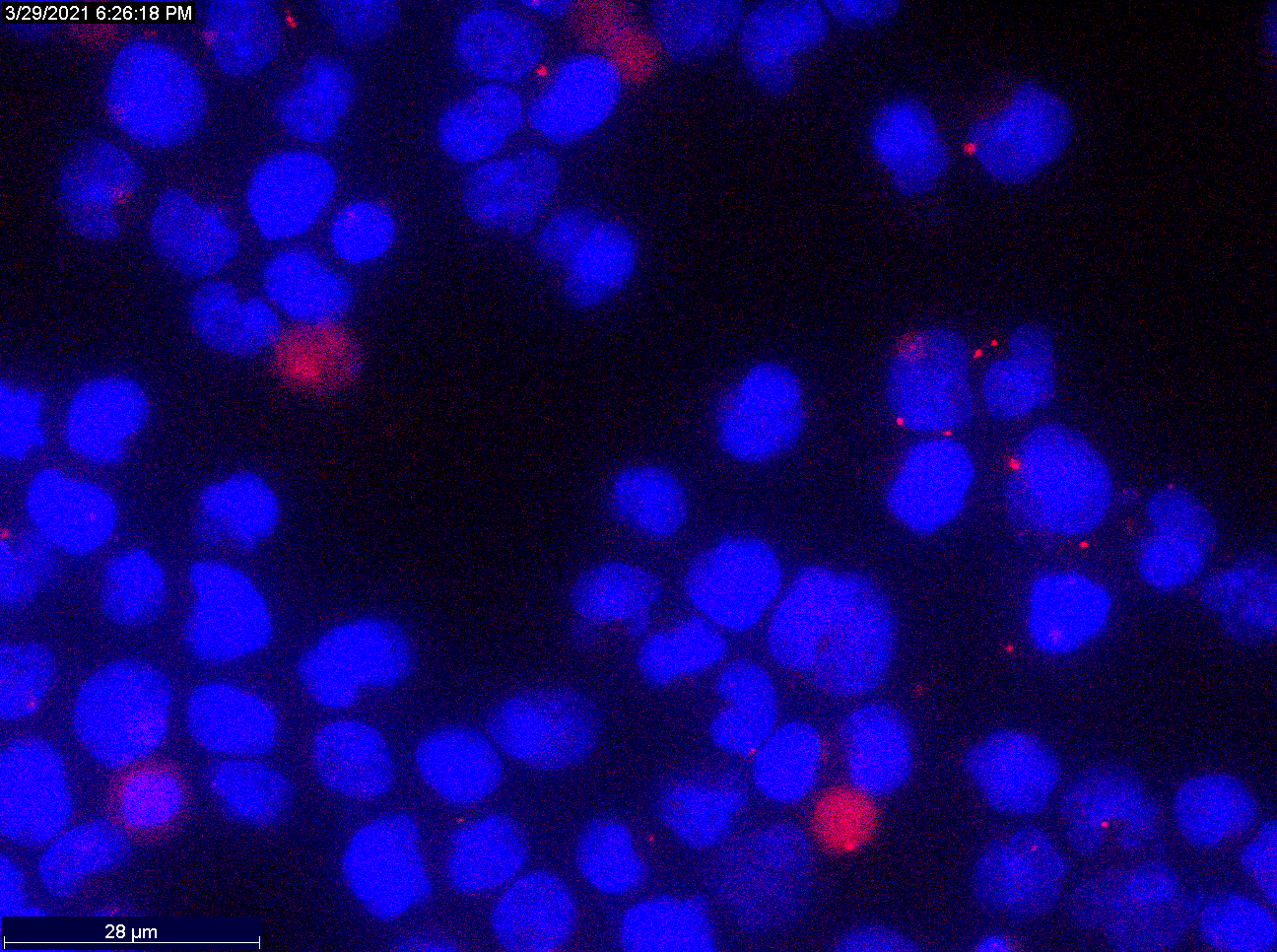

Supplement: Supplementary file 5 — Source data Fig. 1 [file 44318_2026_781_MOESM5_ESM.zip › Figure 1/D/Fig 1 D CD32B_mock CD32B M A M.tif]

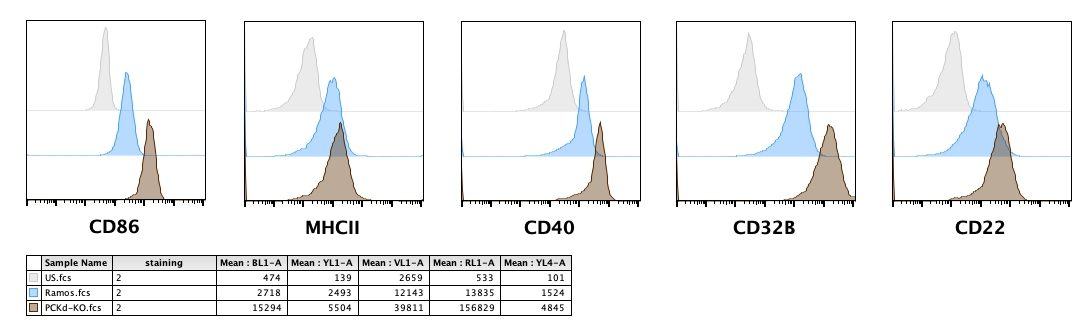

Supplement: Supplementary file 6 — Source data Fig. 2 [file 44318_2026_781_MOESM6_ESM.zip › Figure 2/A/Fig 2A PKCdKO s3-Layout-2.jpg]

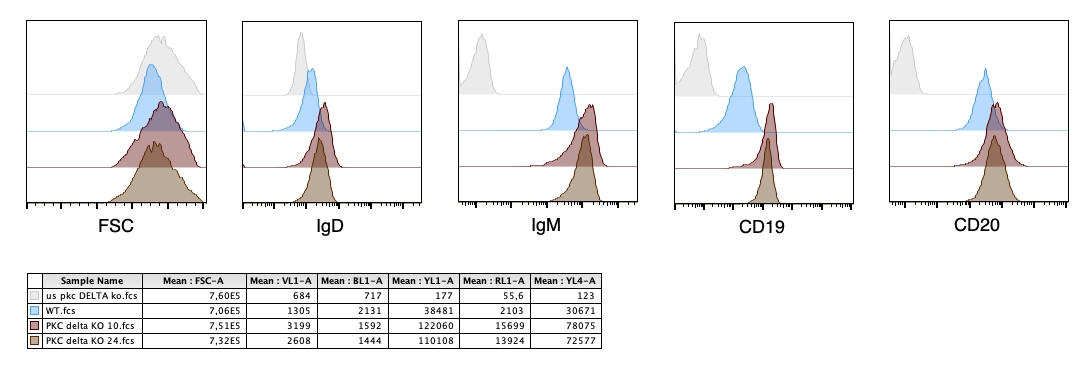

Supplement: Supplementary file 6 — Source data Fig. 2 [file 44318_2026_781_MOESM6_ESM.zip › Figure 2/A/Fig 2A PKCd KO s2-Layout.jpg]

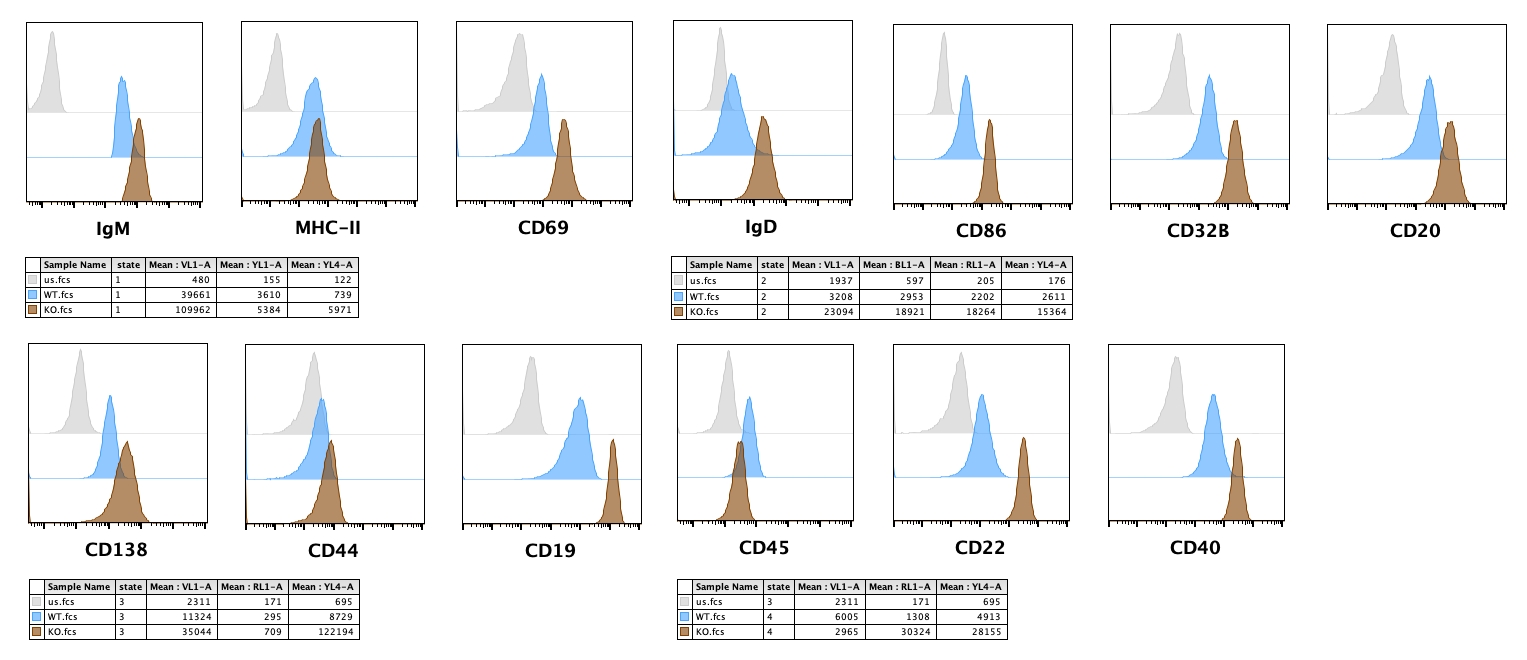

Supplement: Supplementary file 6 — Source data Fig. 2 [file 44318_2026_781_MOESM6_ESM.zip › Figure 2/A/fig 2A PKCd KO S4.jpg]

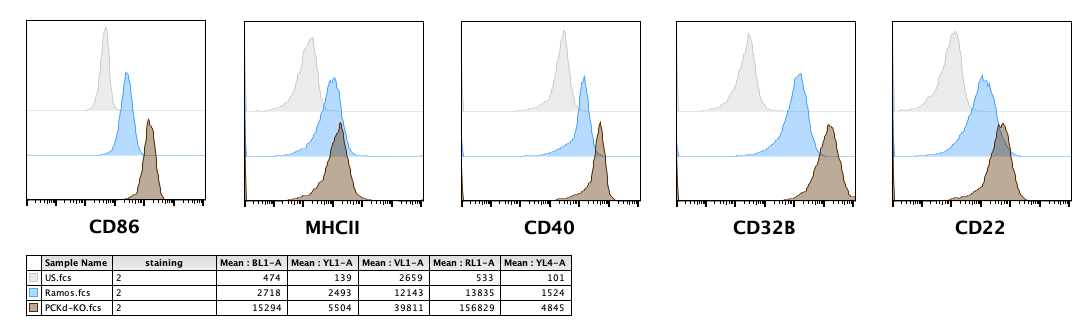

Supplement: Supplementary file 6 — Source data Fig. 2 [file 44318_2026_781_MOESM6_ESM.zip › Figure 2/A/s3 wsp and files/Fig 2A PKCdKO s3-Layout-2.tiff]

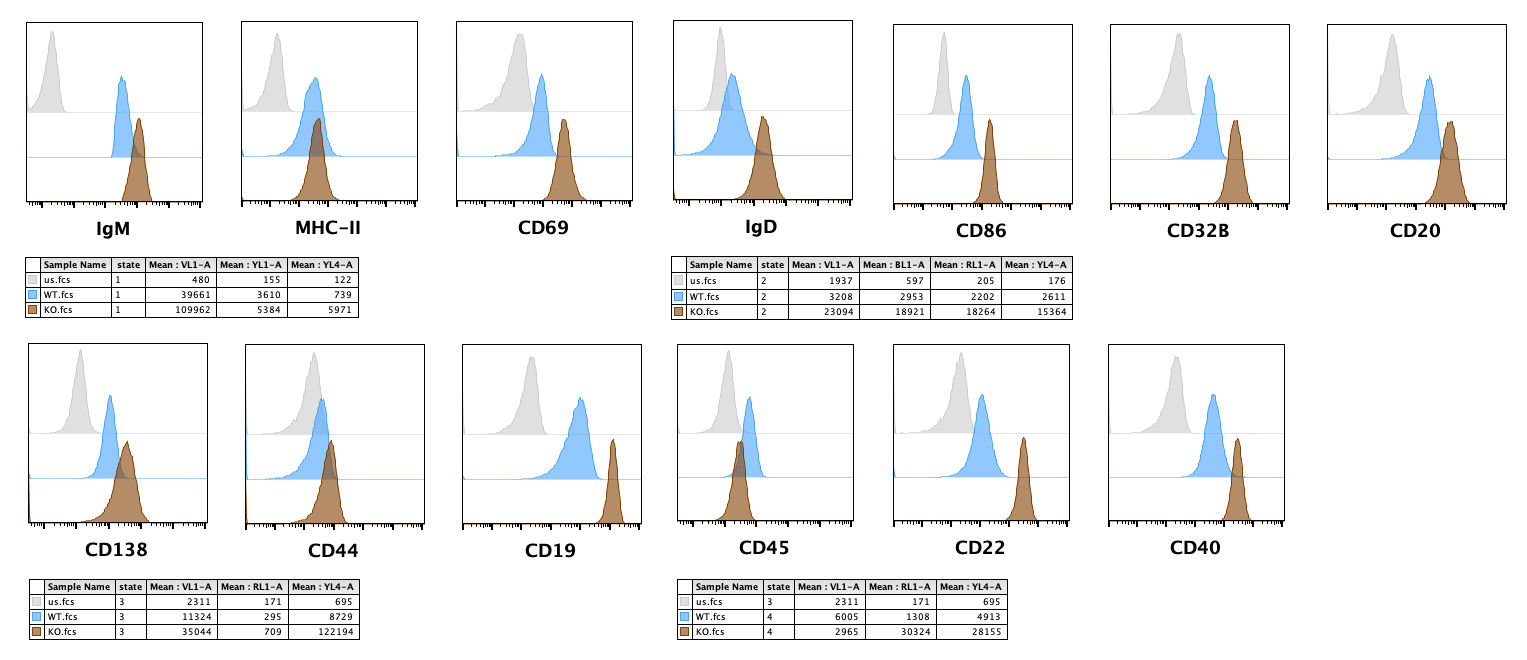

Supplement: Supplementary file 6 — Source data Fig. 2 [file 44318_2026_781_MOESM6_ESM.zip › Figure 2/A/s4 wsp and files/Fig 2A PKCd KO s4-Layout.tiff]

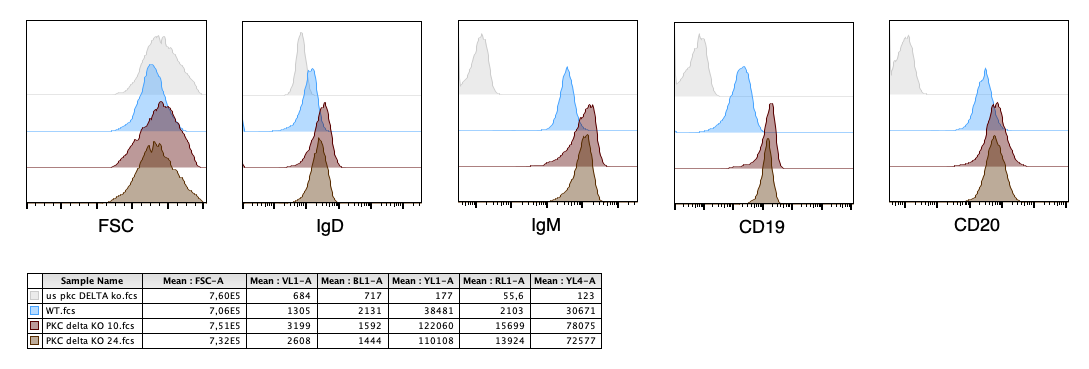

Supplement: Supplementary file 6 — Source data Fig. 2 [file 44318_2026_781_MOESM6_ESM.zip › Figure 2/A/S2 wsp and files/Fig 2A PKCd KO s2-Layout.tiff]

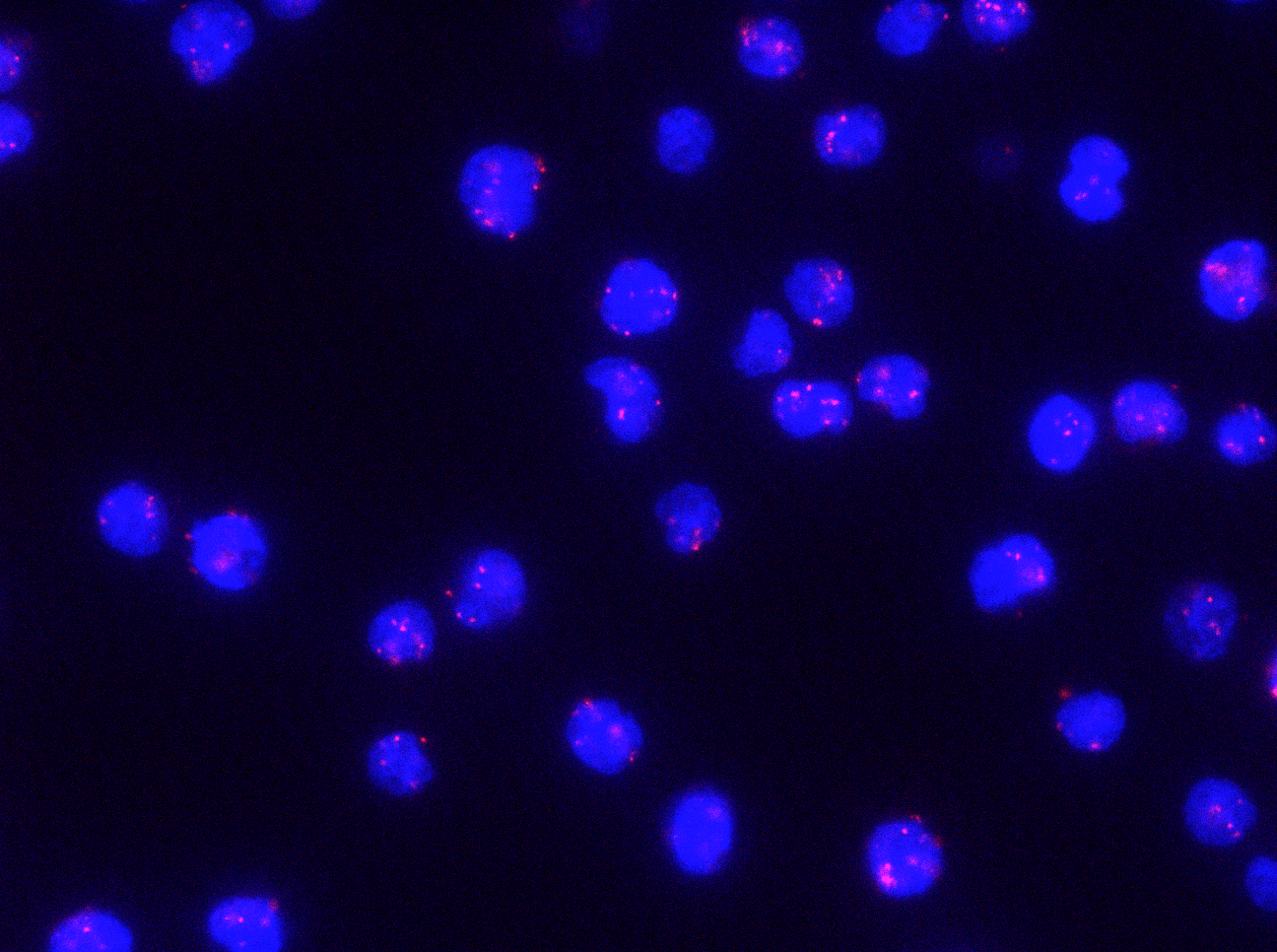

Supplement: Supplementary file 7 — Source data Fig. 3 [file 44318_2026_781_MOESM7_ESM.zip › Figure 3/D/Fig 3D Cmut.png]

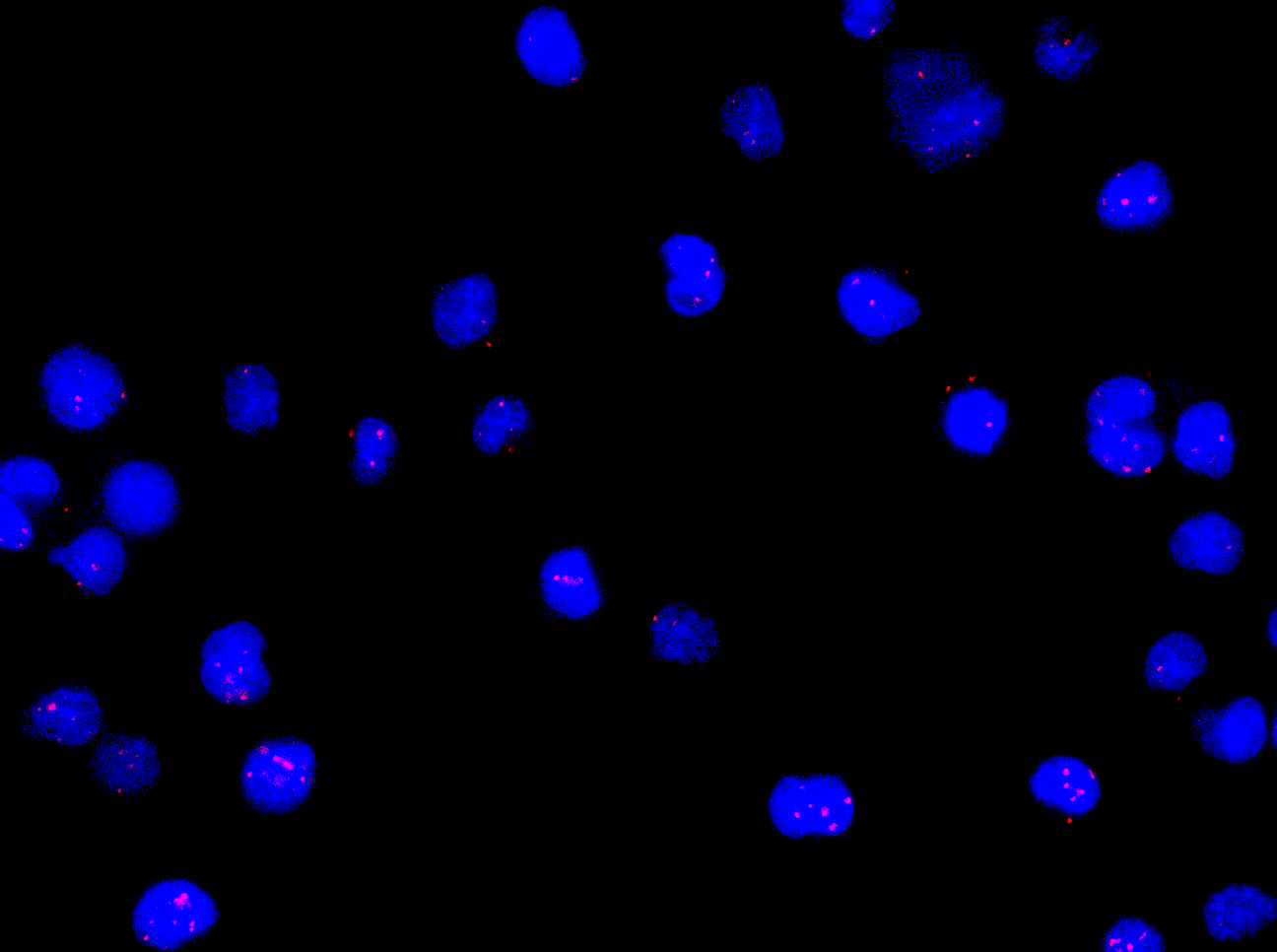

Supplement: Supplementary file 7 — Source data Fig. 3 [file 44318_2026_781_MOESM7_ESM.zip › Figure 3/D/Fig 3D Nmut.png]

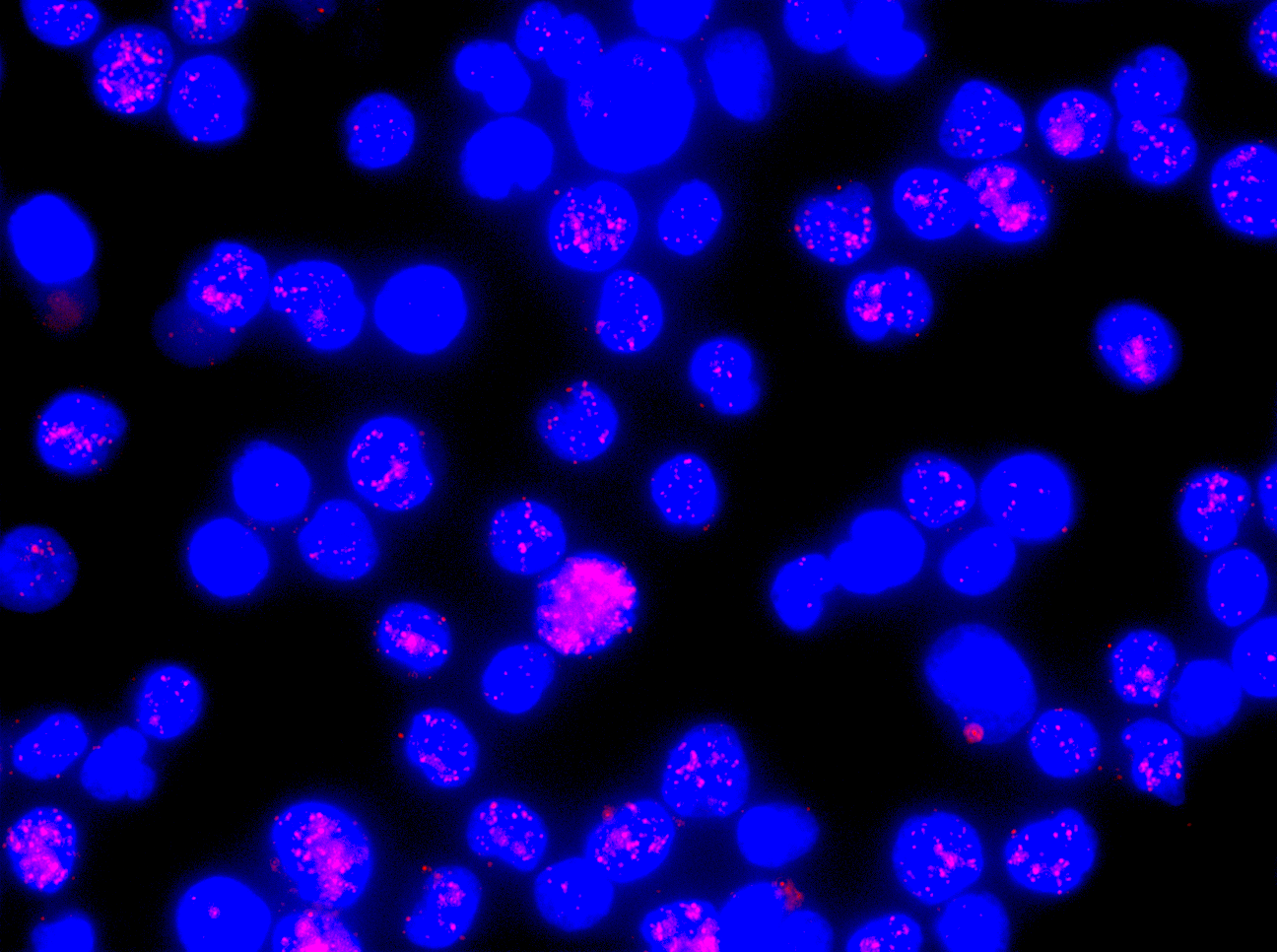

Supplement: Supplementary file 7 — Source data Fig. 3 [file 44318_2026_781_MOESM7_ESM.zip › Figure 3/D/Fig 3D WT paper.png]

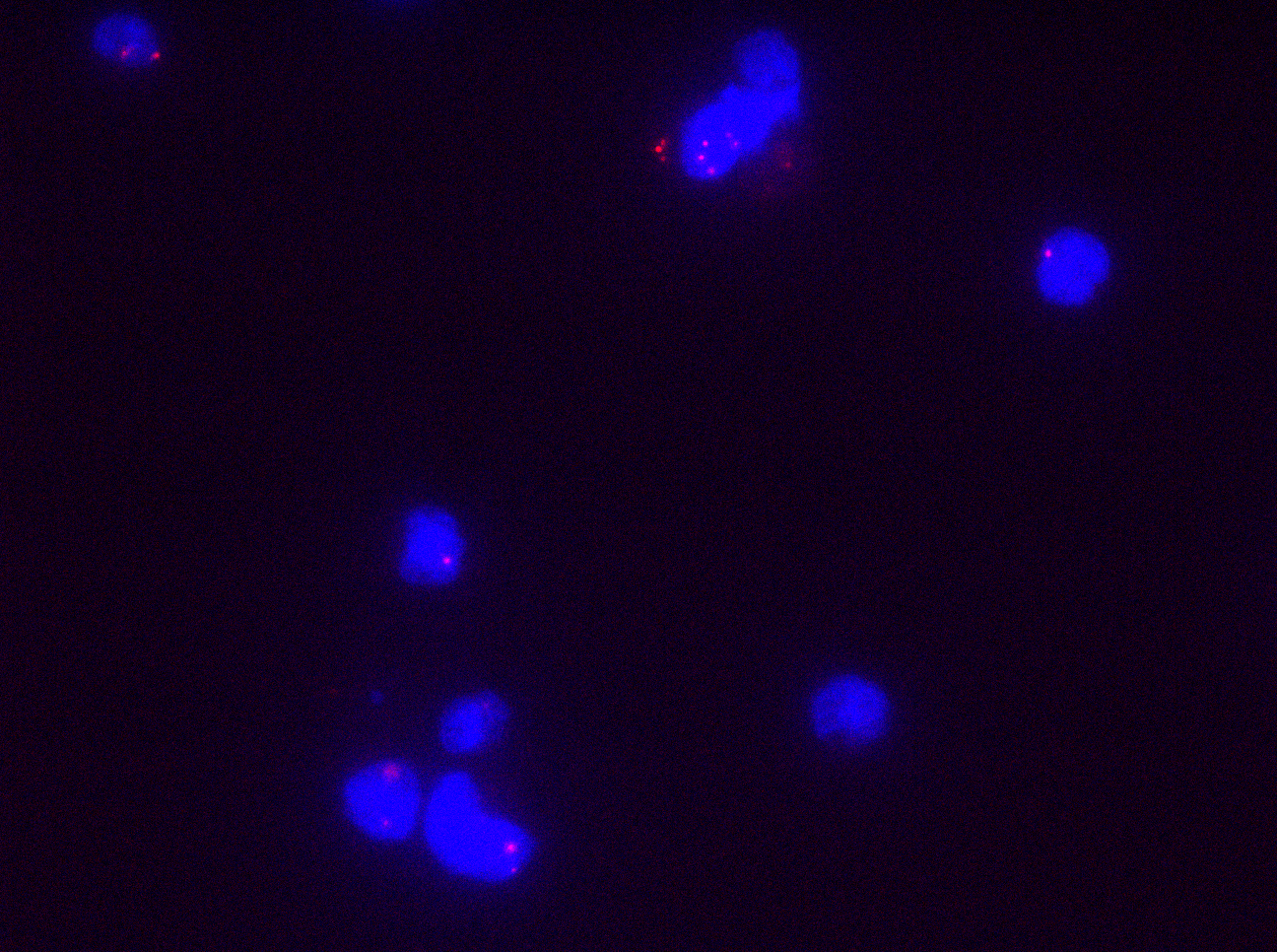

Supplement: Supplementary file 7 — Source data Fig. 3 [file 44318_2026_781_MOESM7_ESM.zip › Figure 3/D/Fig 3D NCmut.png]

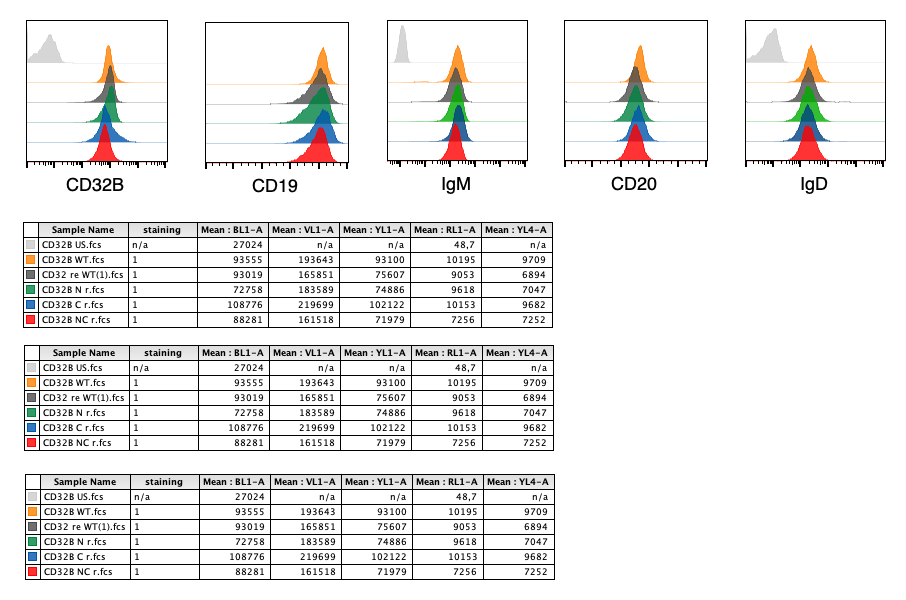

Supplement: Supplementary file 7 — Source data Fig. 3 [file 44318_2026_781_MOESM7_ESM.zip › Figure 3/C/Fig 3 C -1/Fig 3 C-1 CD20 mutants extra-Layout-4.tiff]

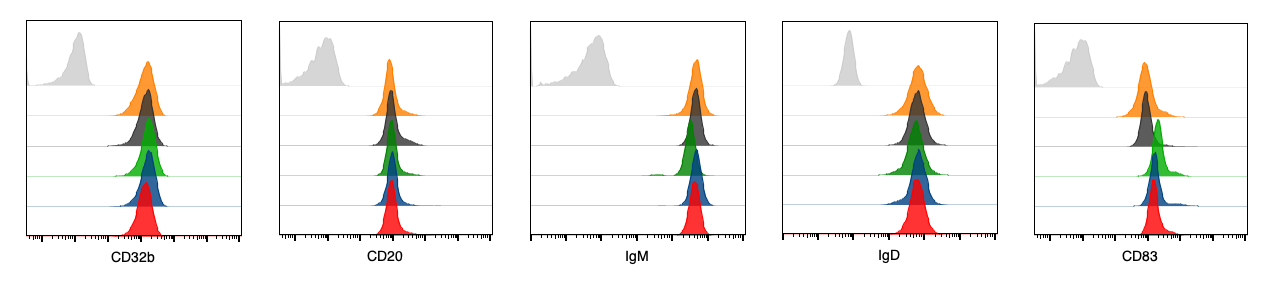

Supplement: Supplementary file 7 — Source data Fig. 3 [file 44318_2026_781_MOESM7_ESM.zip › Figure 3/C/Fig 3 C -2/Fig. 3 C-2 CD20 mut extra paper-Layout-2.tiff]

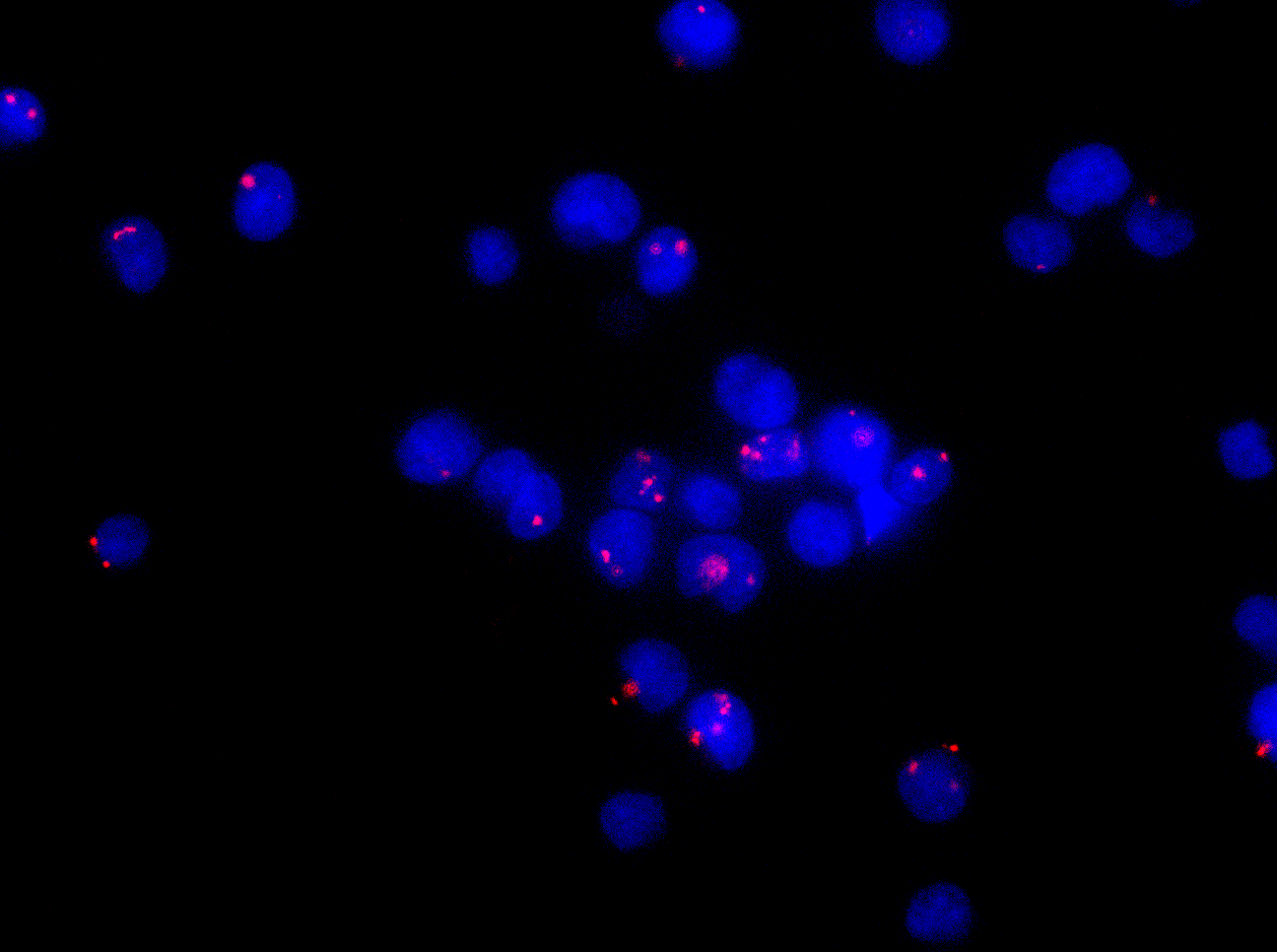

Supplement: Supplementary file 8 — Source data Fig. 4 [file 44318_2026_781_MOESM8_ESM.zip › Figure 4/B/Fig 4 B Nmut paper.png]

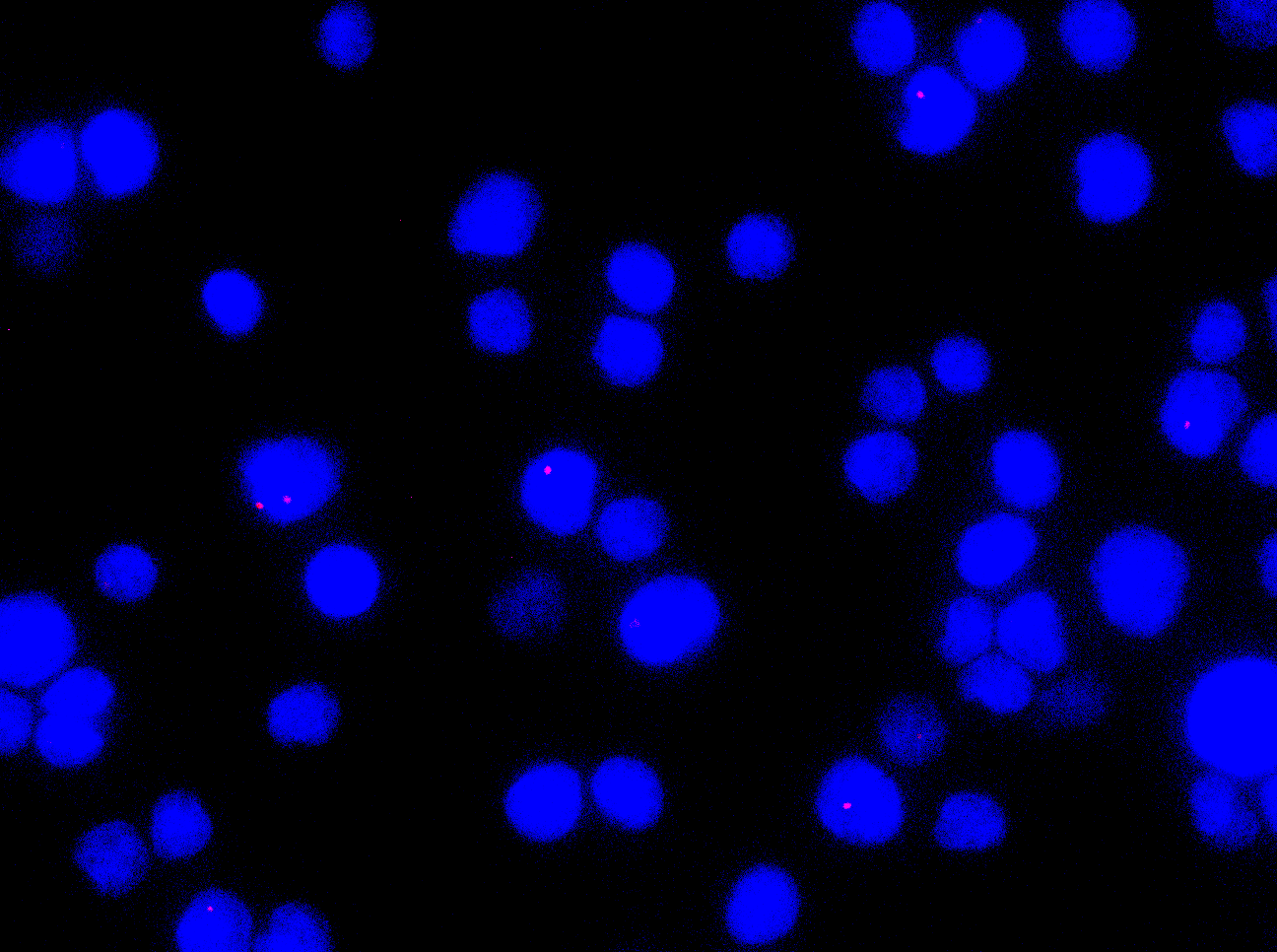

Supplement: Supplementary file 8 — Source data Fig. 4 [file 44318_2026_781_MOESM8_ESM.zip › Figure 4/B/Fig 4 B NC mut paper.png]

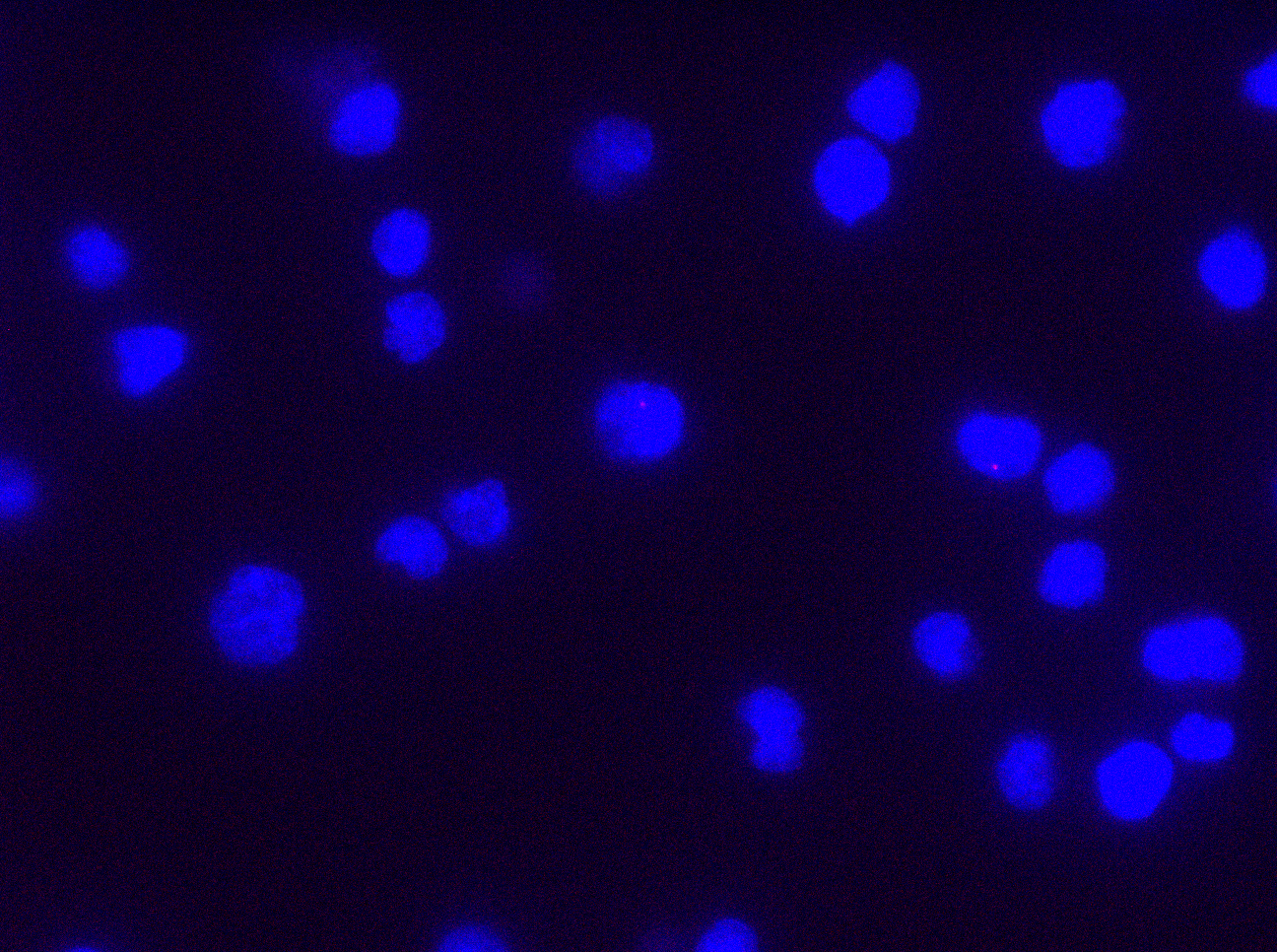

Supplement: Supplementary file 8 — Source data Fig. 4 [file 44318_2026_781_MOESM8_ESM.zip › Figure 4/B/Fig 4 B Cmut paper.png]

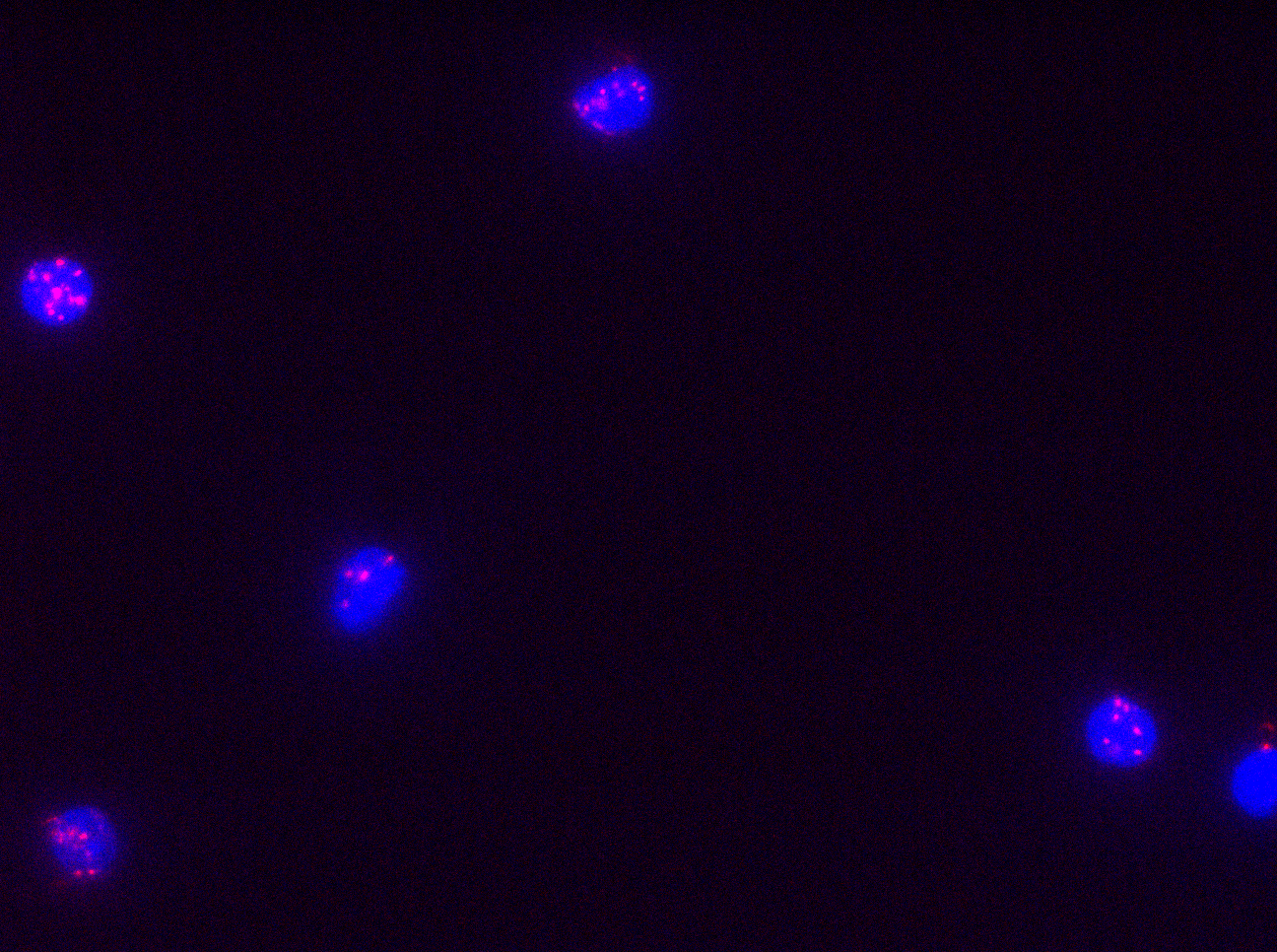

Supplement: Supplementary file 8 — Source data Fig. 4 [file 44318_2026_781_MOESM8_ESM.zip › Figure 4/B/Fig 4 B WT paper.png]

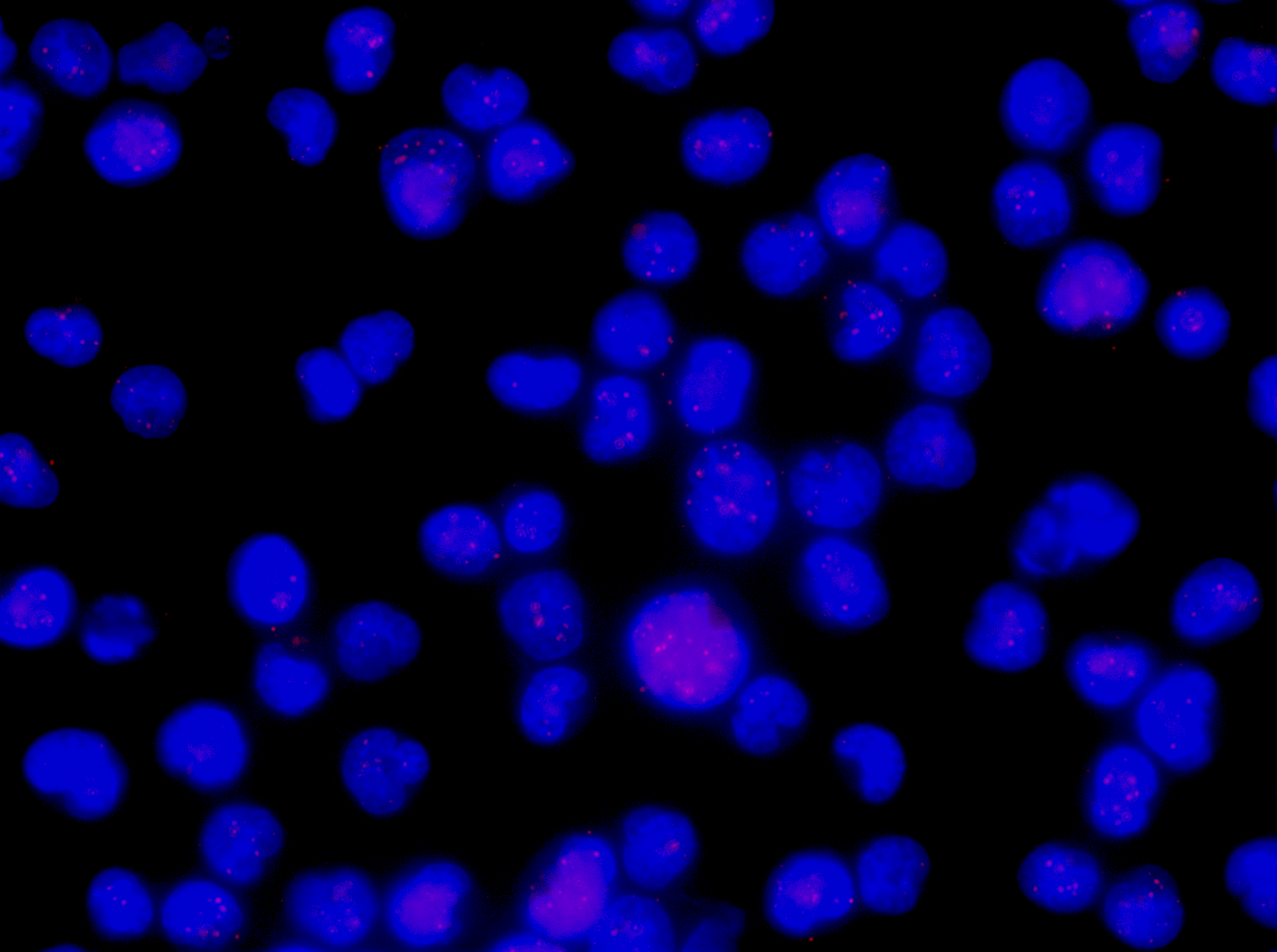

Supplement: Supplementary file 9 — Source data Fig. 5 [file 44318_2026_781_MOESM9_ESM.zip › Figure 5/G/Fig 5G CD32b.png]

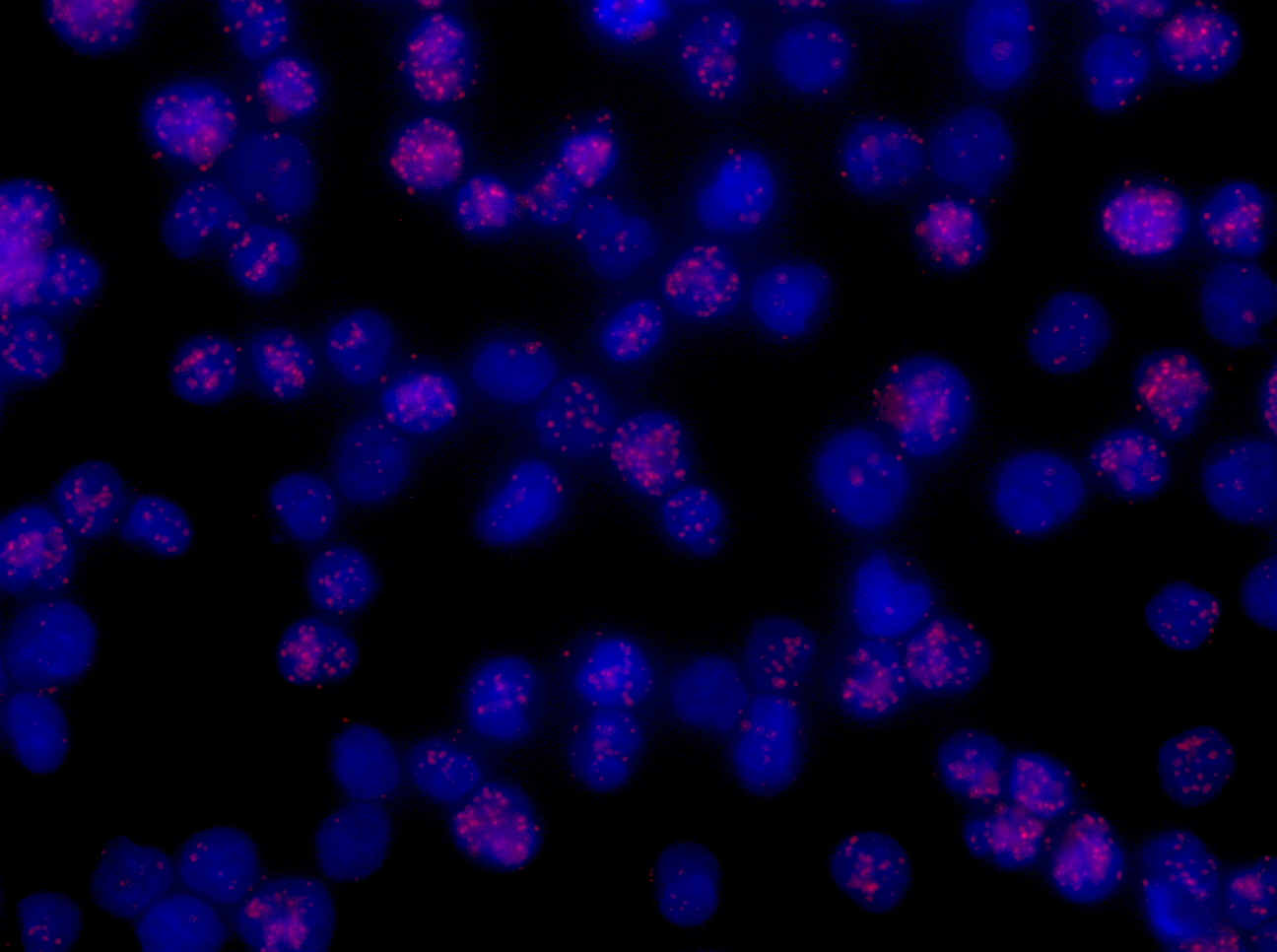

Supplement: Supplementary file 9 — Source data Fig. 5 [file 44318_2026_781_MOESM9_ESM.zip › Figure 5/G/Fig 5G WT RTX.png]

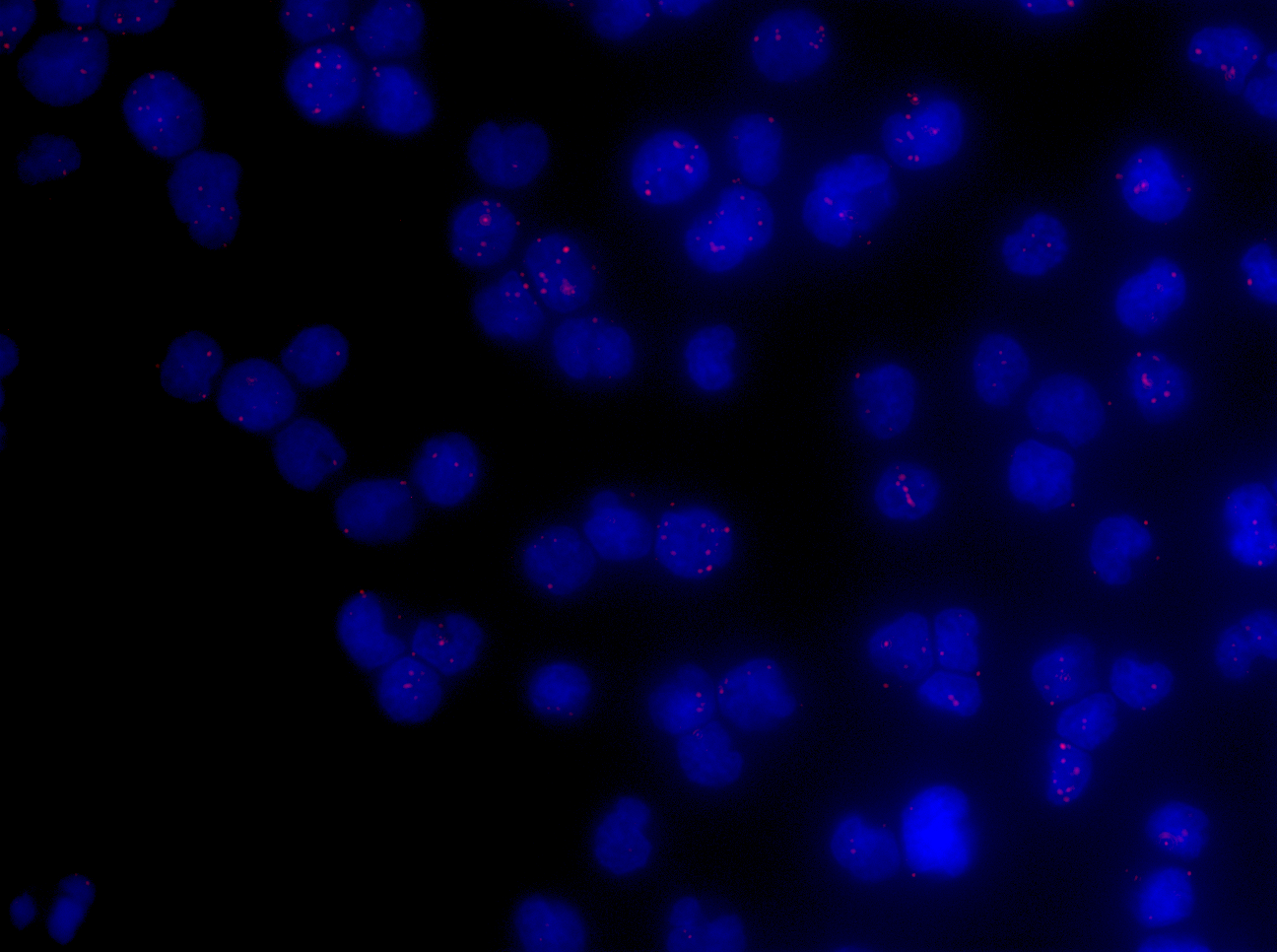

Supplement: Supplementary file 9 — Source data Fig. 5 [file 44318_2026_781_MOESM9_ESM.zip › Figure 5/G/Fig 5G 2 WT.png]

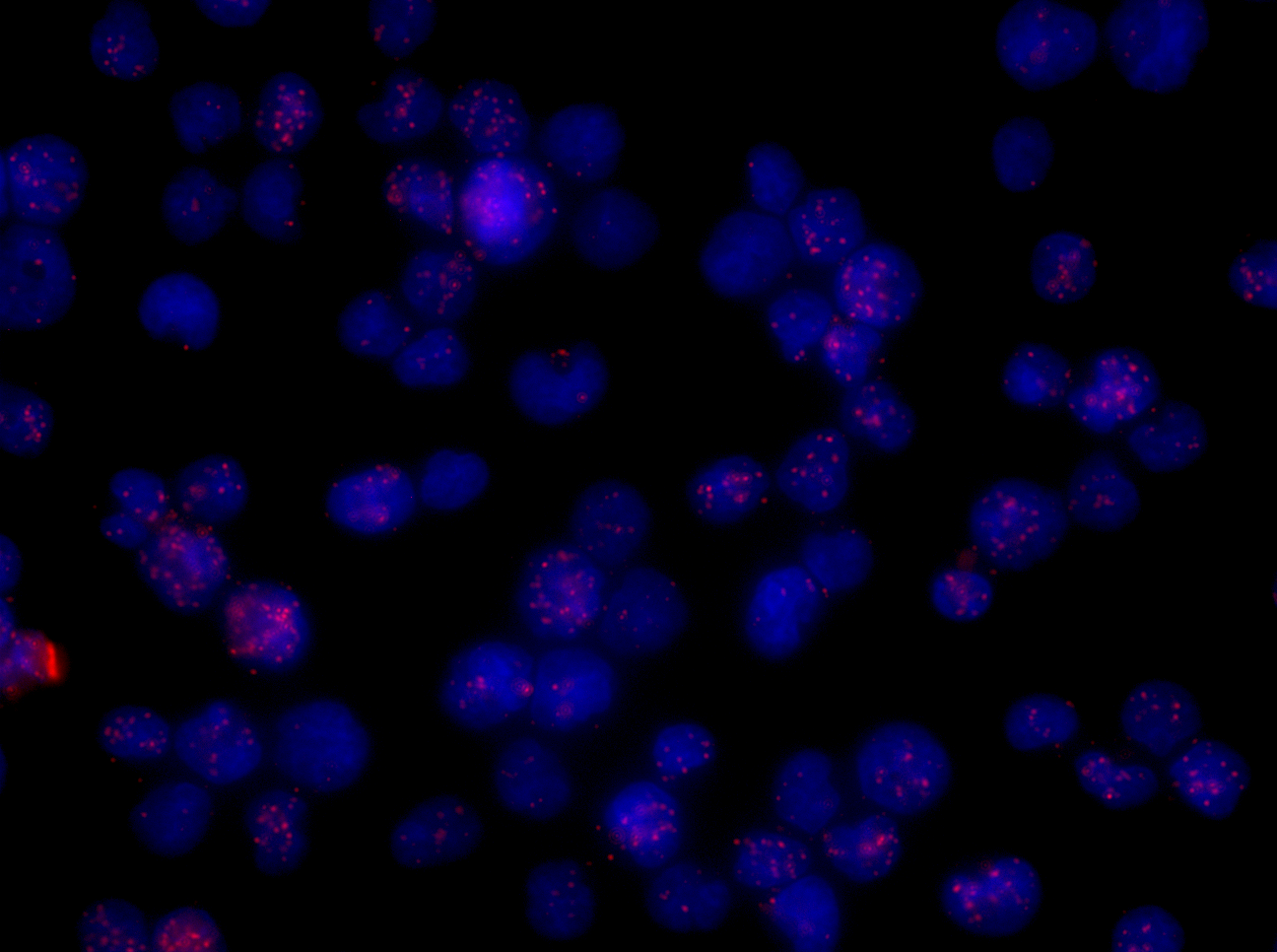

Supplement: Supplementary file 9 — Source data Fig. 5 [file 44318_2026_781_MOESM9_ESM.zip › Figure 5/G/Fig 5F CD32b RTX.png]

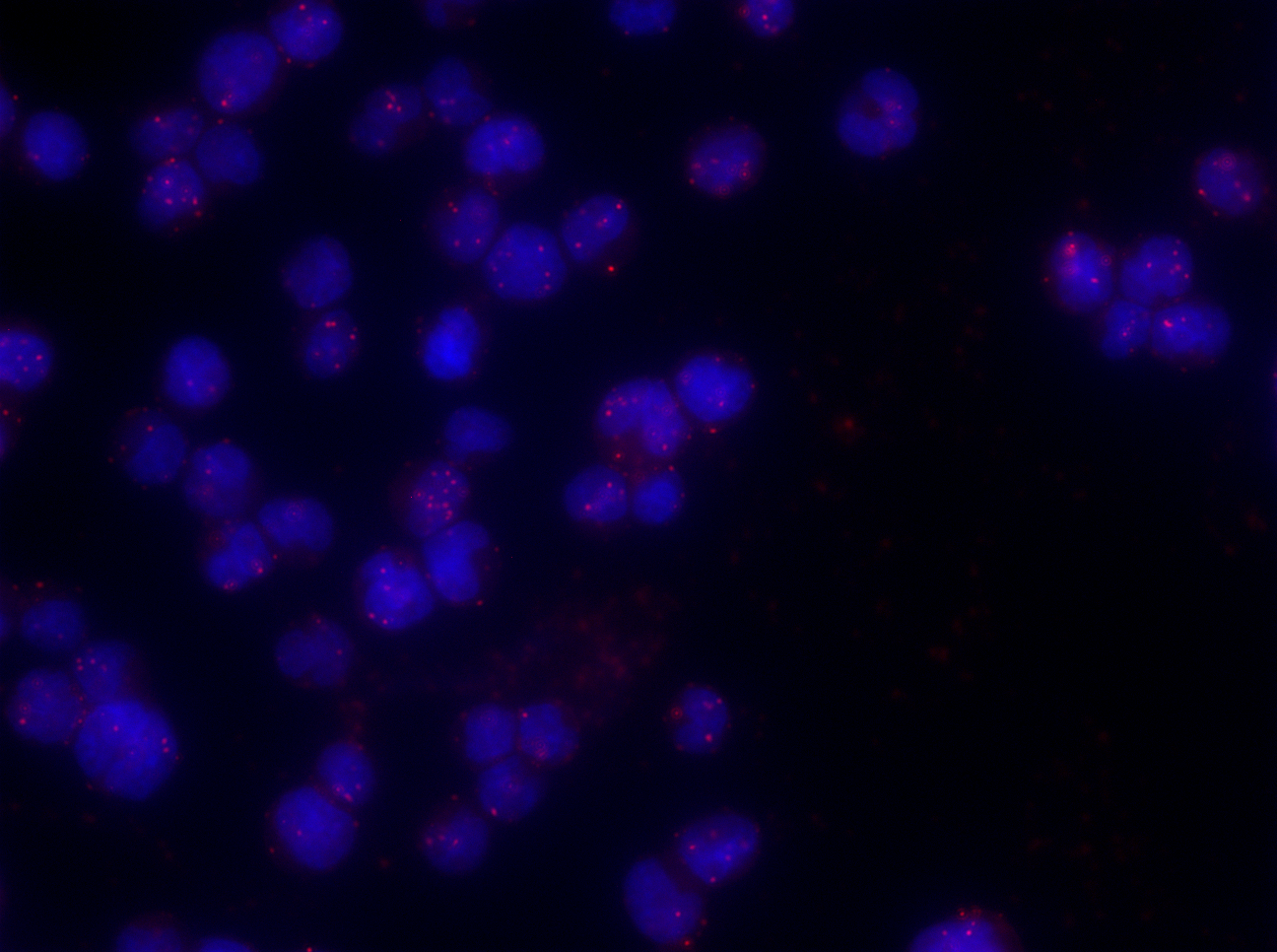

Supplement: Supplementary file 9 — Source data Fig. 5 [file 44318_2026_781_MOESM9_ESM.zip › Figure 5/G/Fig 5G NCmut RTX.png]

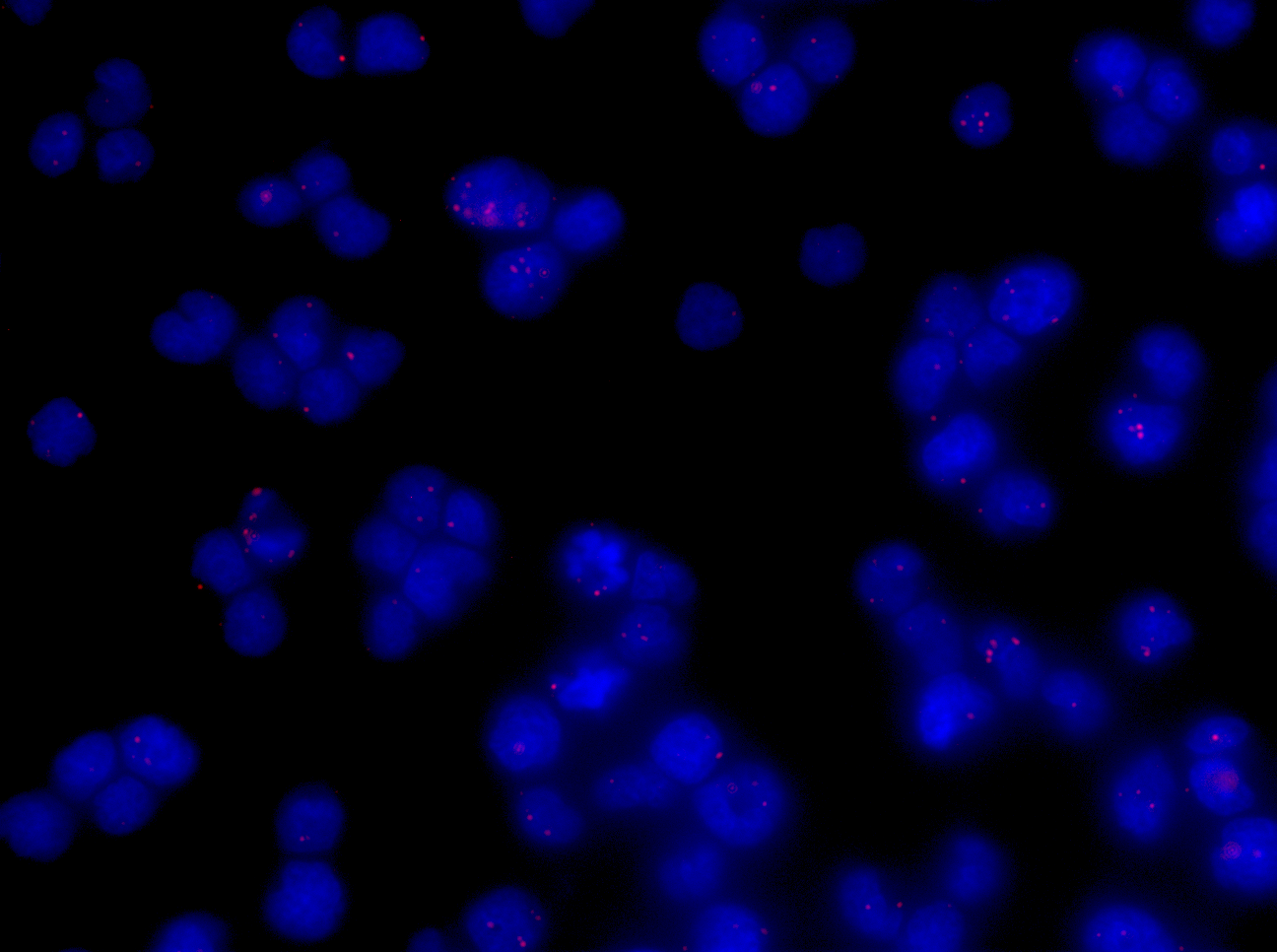

Supplement: Supplementary file 9 — Source data Fig. 5 [file 44318_2026_781_MOESM9_ESM.zip › Figure 5/G/Fig 5G NCmut.png]

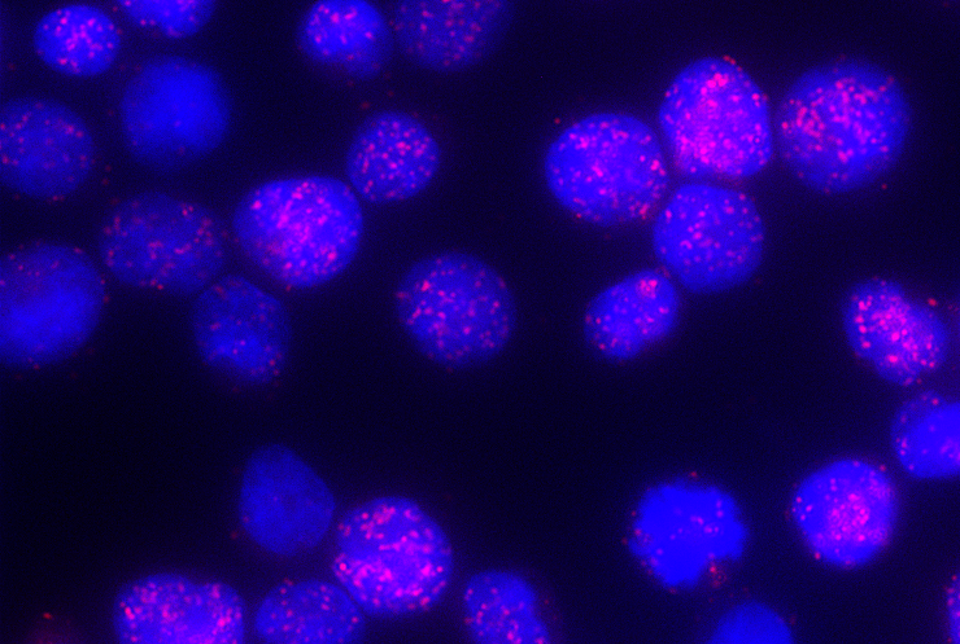

Supplement: Supplementary file 9 — Source data Fig. 5 [file 44318_2026_781_MOESM9_ESM.zip › Figure 5/A/Fig 5A CD32B.png]

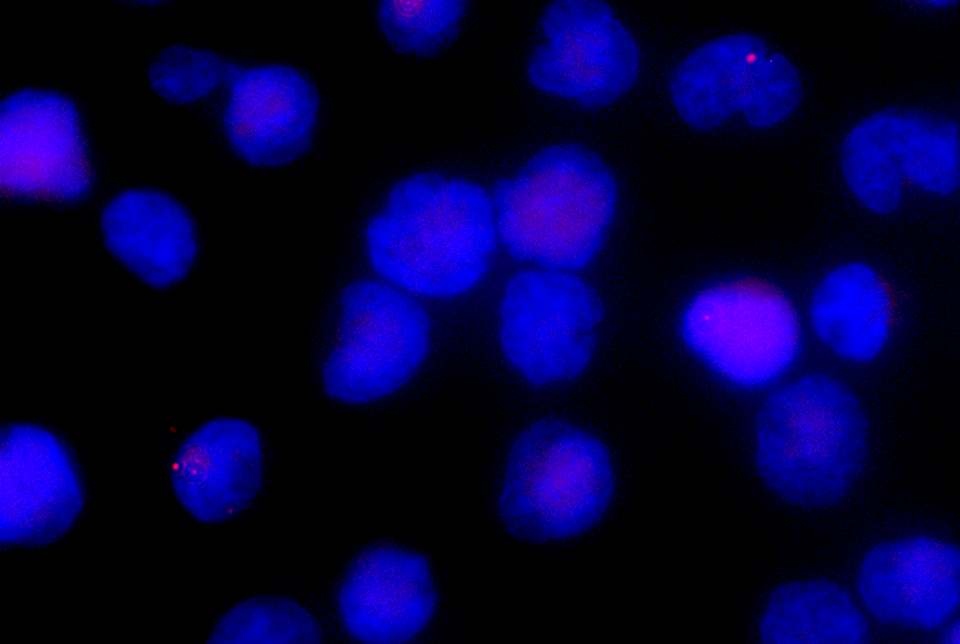

Supplement: Supplementary file 9 — Source data Fig. 5 [file 44318_2026_781_MOESM9_ESM.zip › Figure 5/A/Fig 5A NCmut RTX.png]

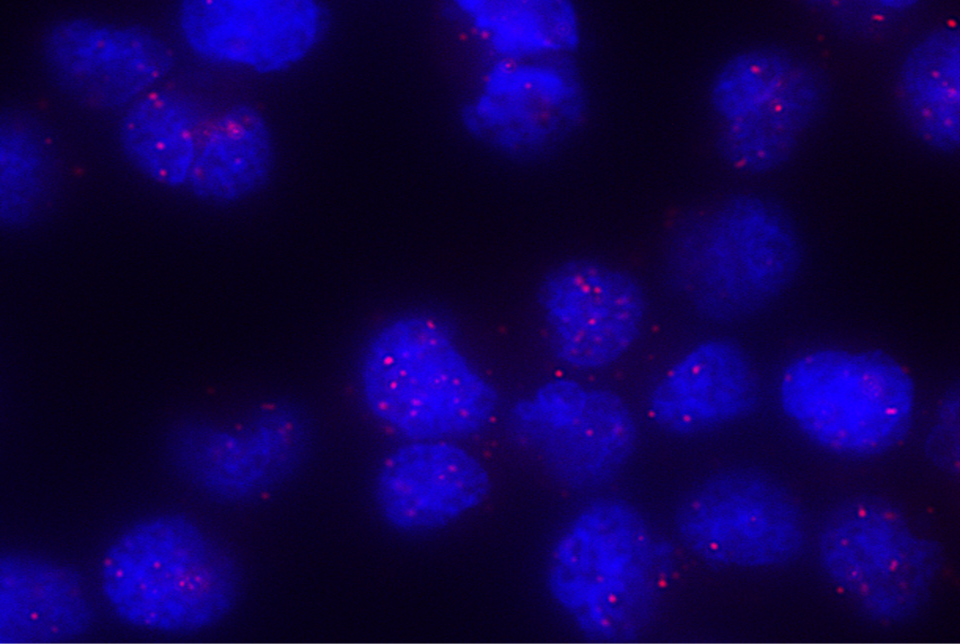

Supplement: Supplementary file 9 — Source data Fig. 5 [file 44318_2026_781_MOESM9_ESM.zip › Figure 5/A/Fig 5 A CD32B RTX.png]

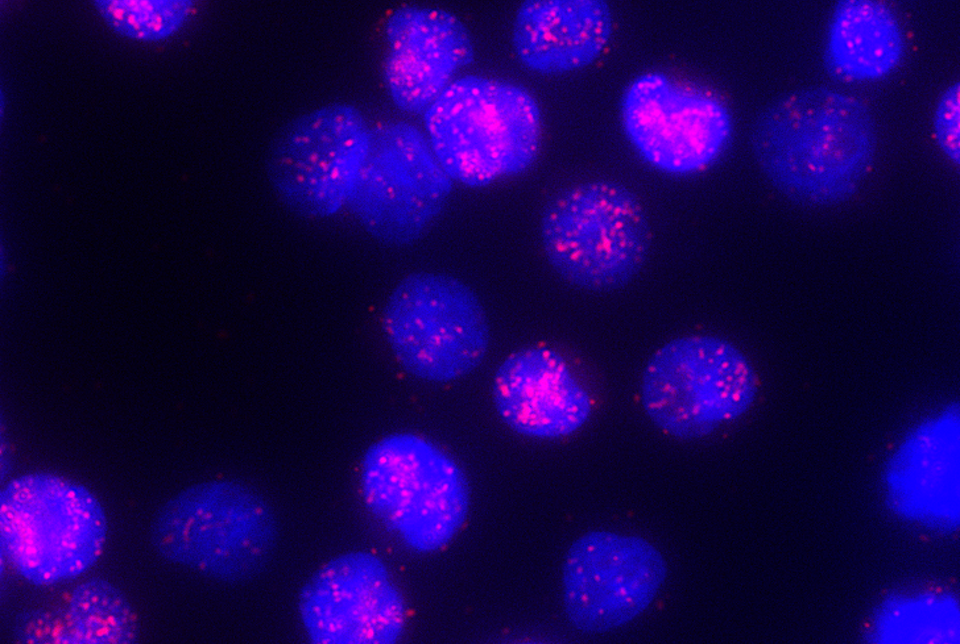

Supplement: Supplementary file 9 — Source data Fig. 5 [file 44318_2026_781_MOESM9_ESM.zip › Figure 5/A/Fig 5A WT.png]

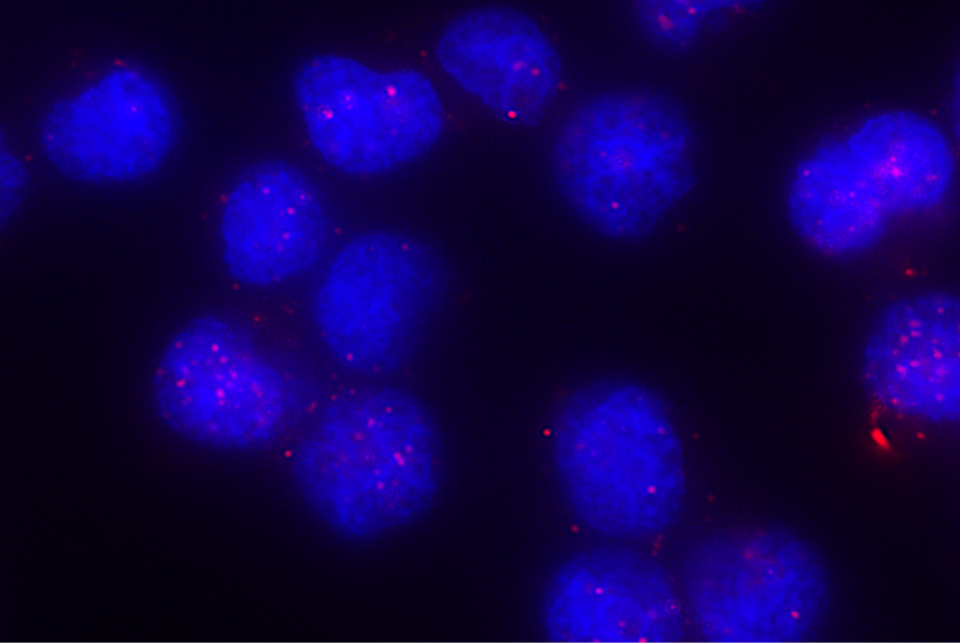

Supplement: Supplementary file 9 — Source data Fig. 5 [file 44318_2026_781_MOESM9_ESM.zip › Figure 5/A/Fig 5 A WT RTX.png]

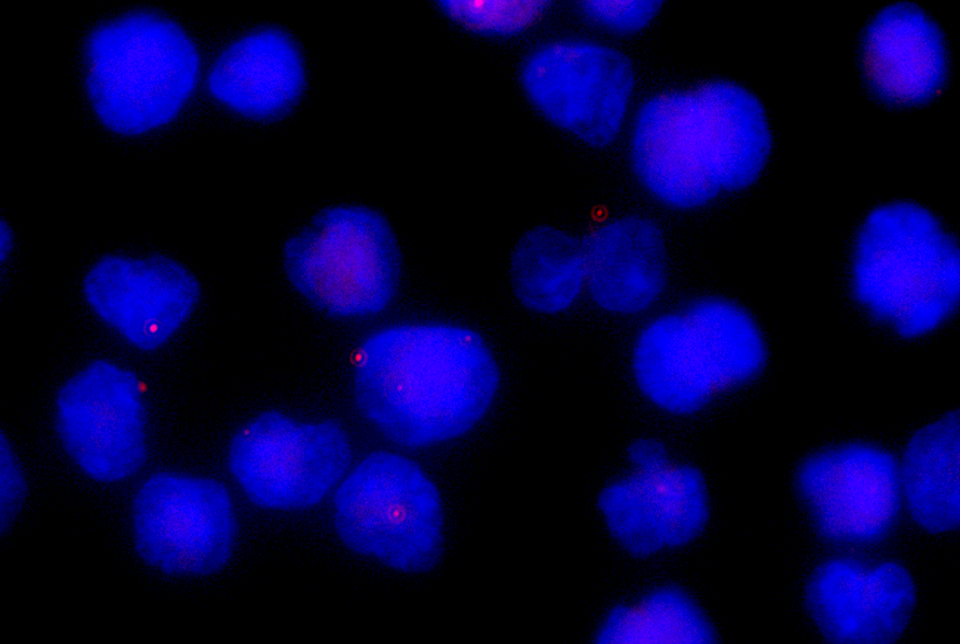

Supplement: Supplementary file 9 — Source data Fig. 5 [file 44318_2026_781_MOESM9_ESM.zip › Figure 5/A/Fig 5A NCmut.png]

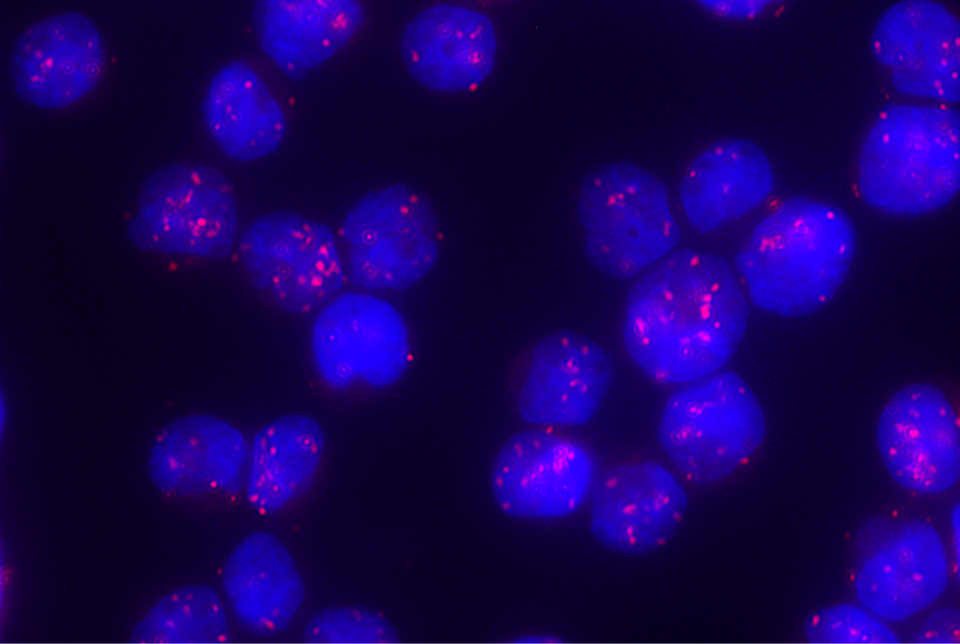

Supplement: Supplementary file 9 — Source data Fig. 5 [file 44318_2026_781_MOESM9_ESM.zip › Figure 5/C/Fig 5C WT.png]

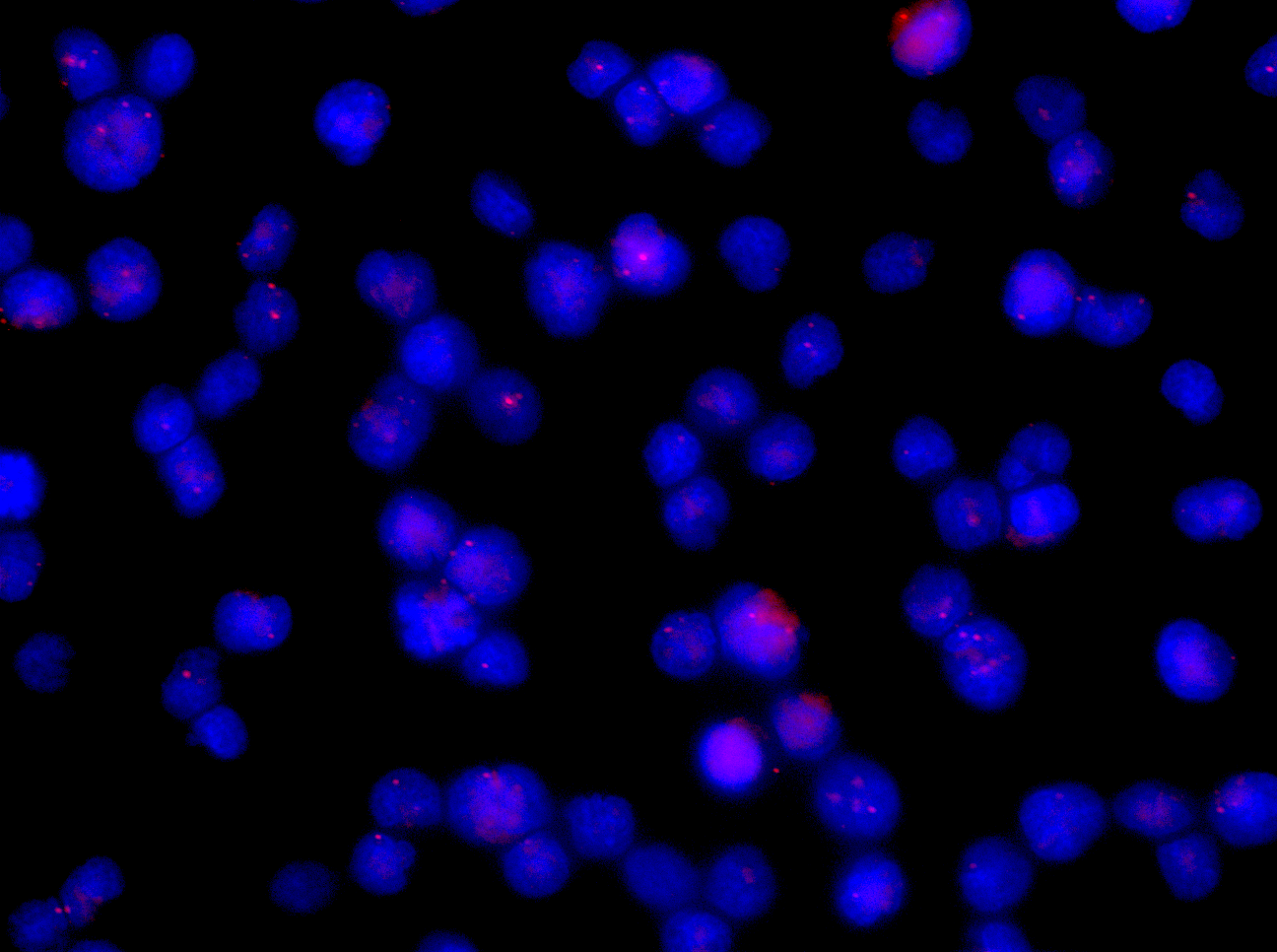

Supplement: Supplementary file 9 — Source data Fig. 5 [file 44318_2026_781_MOESM9_ESM.zip › Figure 5/C/Fig 5C NCmut.png]

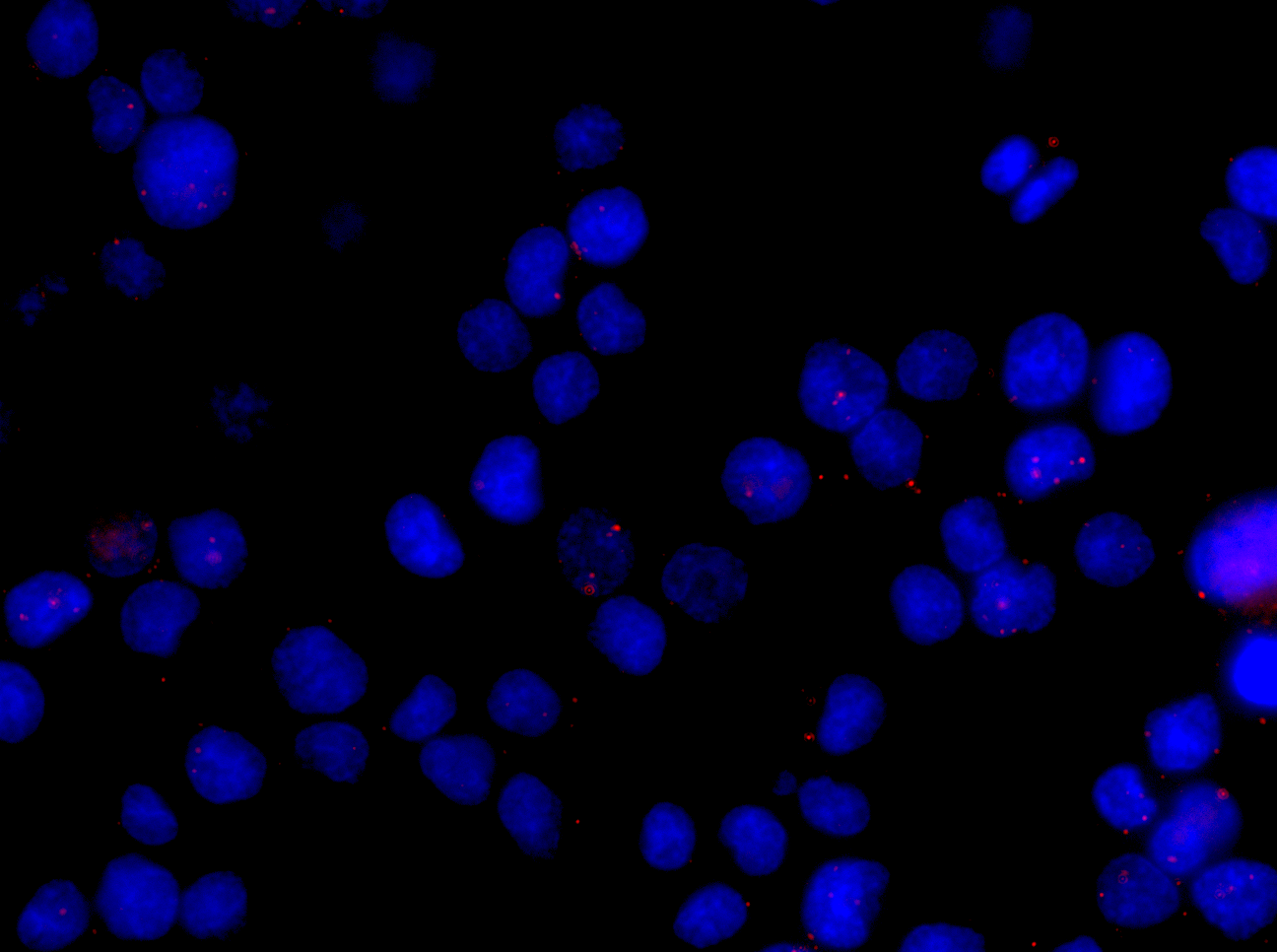

Supplement: Supplementary file 9 — Source data Fig. 5 [file 44318_2026_781_MOESM9_ESM.zip › Figure 5/C/Fig 5C CD32b RTX.png]

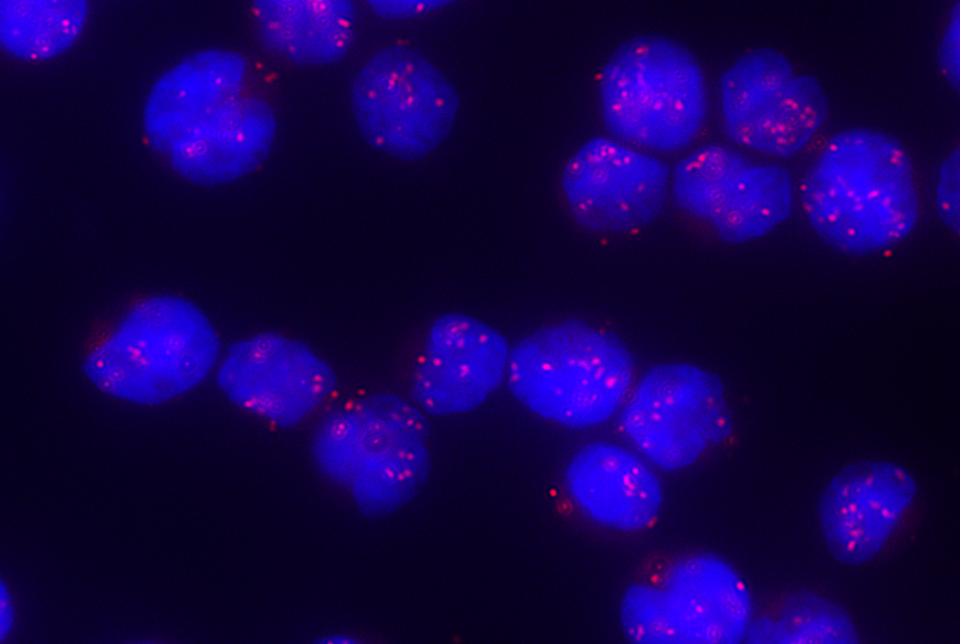

Supplement: Supplementary file 9 — Source data Fig. 5 [file 44318_2026_781_MOESM9_ESM.zip › Figure 5/C/Fig 5C CD32b.png]

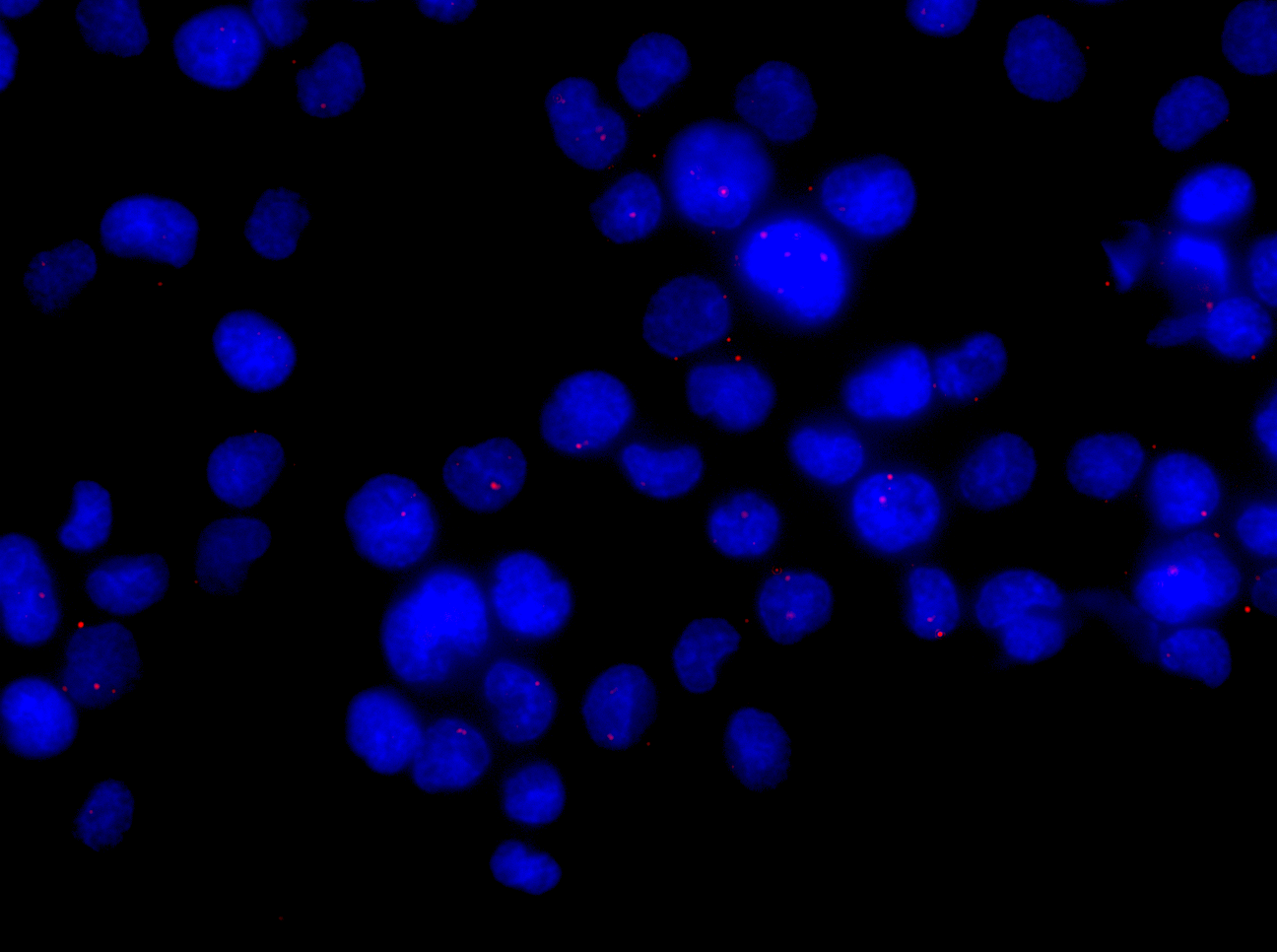

Supplement: Supplementary file 9 — Source data Fig. 5 [file 44318_2026_781_MOESM9_ESM.zip › Figure 5/C/Fig 5C WT RTX.png]

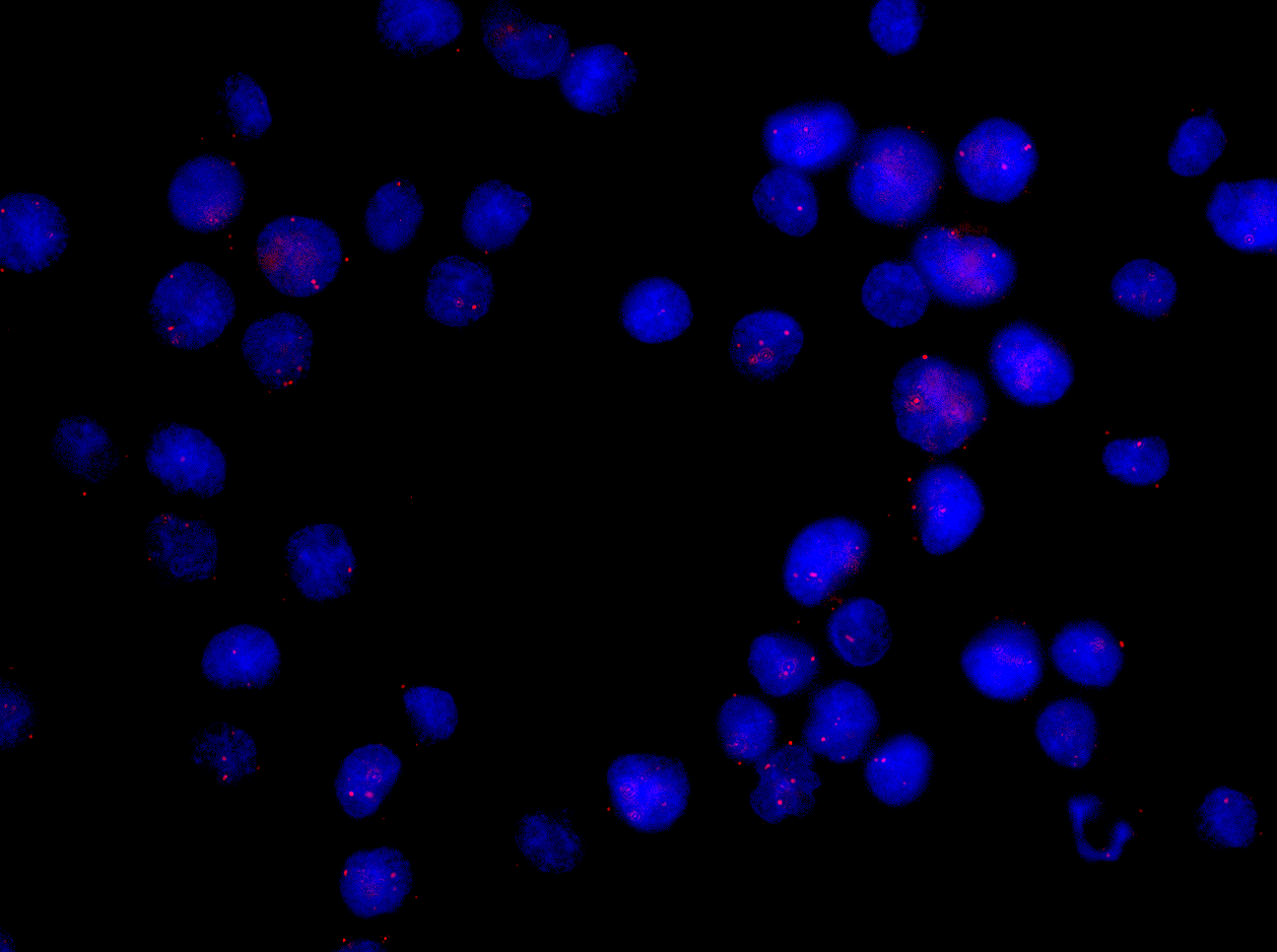

Supplement: Supplementary file 9 — Source data Fig. 5 [file 44318_2026_781_MOESM9_ESM.zip › Figure 5/C/Fig 5C NCmut RTX.png]

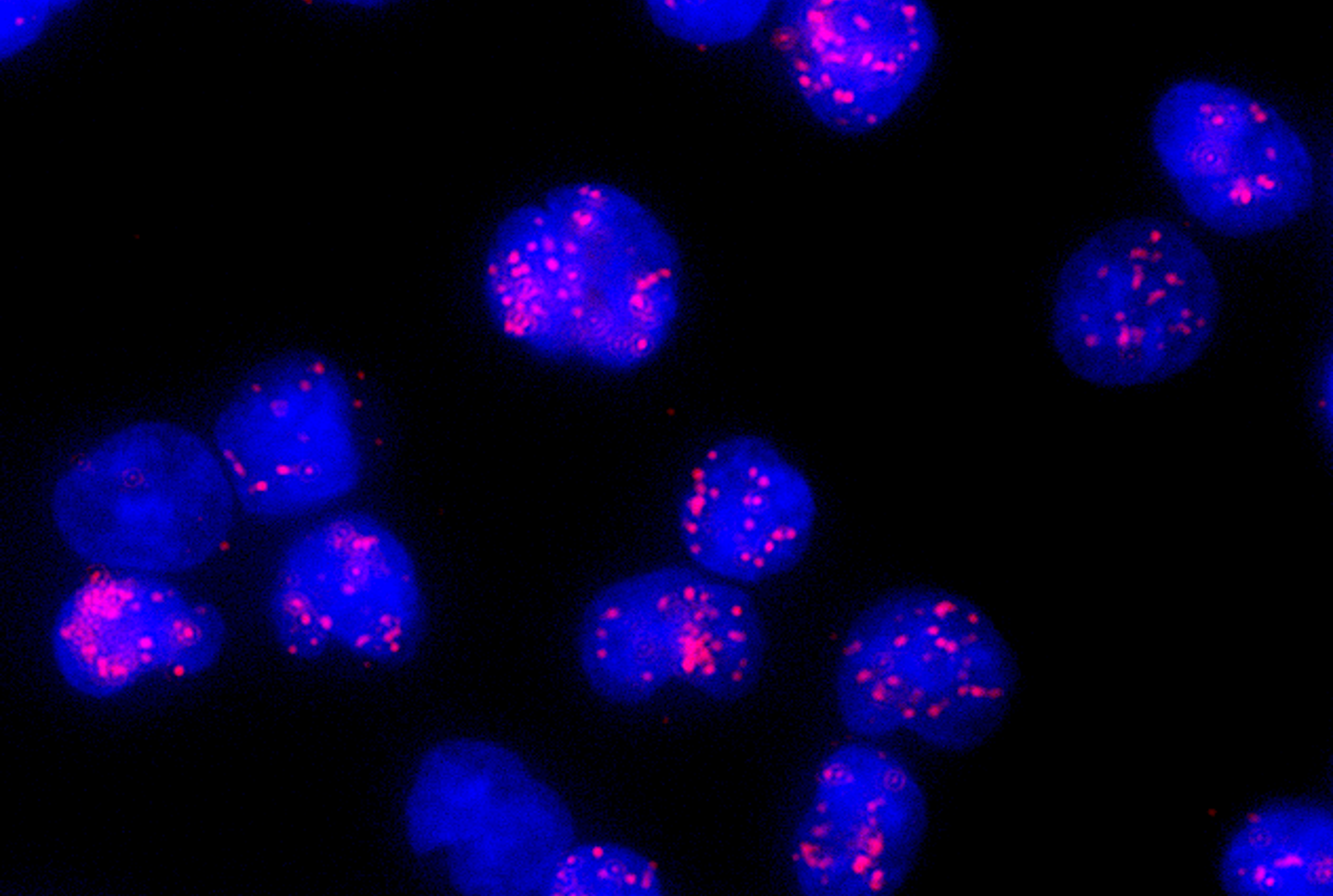

Supplement: Supplementary file 9 — Source data Fig. 5 [file 44318_2026_781_MOESM9_ESM.zip › Figure 5/E/Fig 5E WT RTX.png]

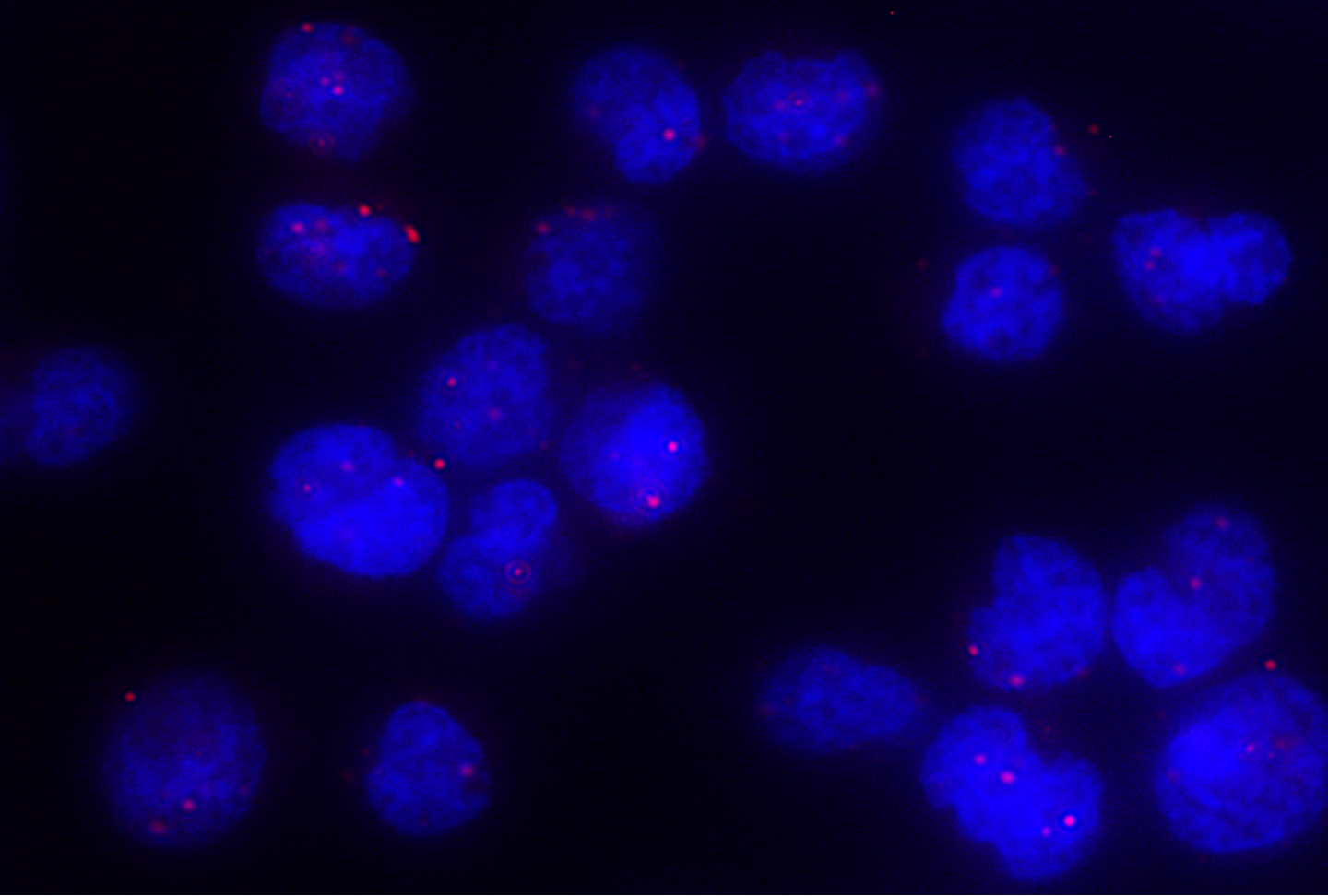

Supplement: Supplementary file 9 — Source data Fig. 5 [file 44318_2026_781_MOESM9_ESM.zip › Figure 5/E/Fig 5E WT.png]

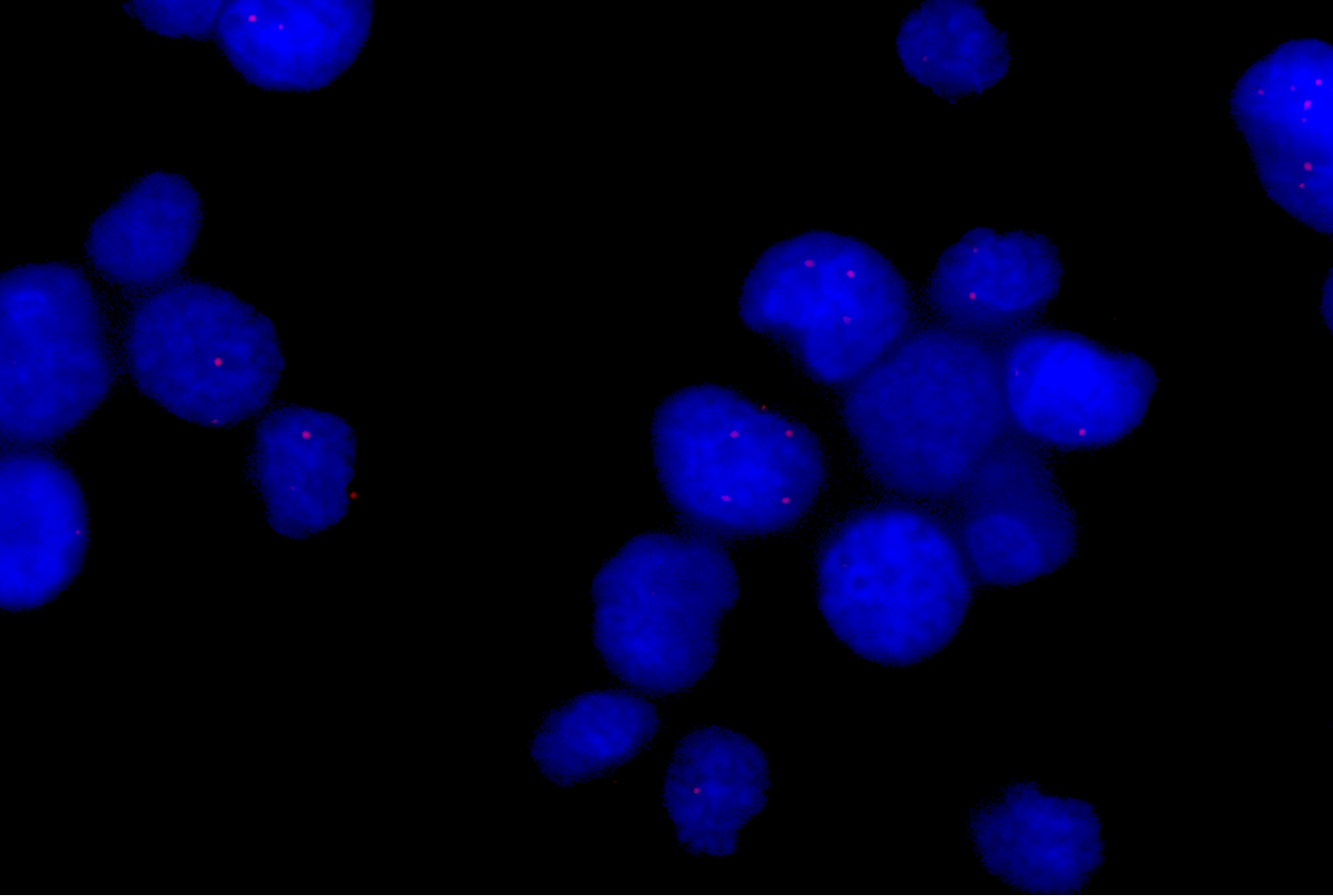

Supplement: Supplementary file 9 — Source data Fig. 5 [file 44318_2026_781_MOESM9_ESM.zip › Figure 5/E/Fig 5E NCmut RTX.png]

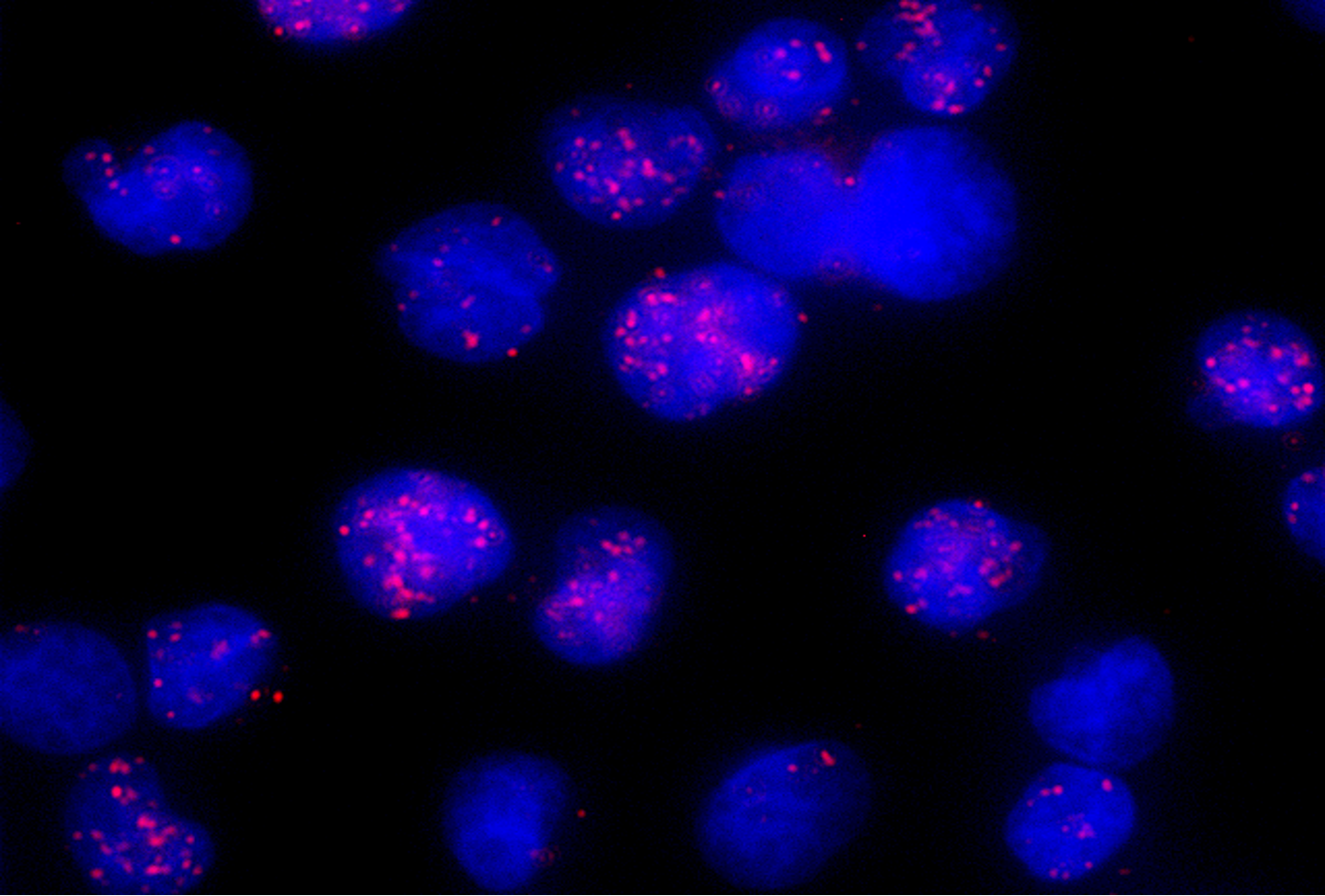

Supplement: Supplementary file 9 — Source data Fig. 5 [file 44318_2026_781_MOESM9_ESM.zip › Figure 5/E/Fig 5E CD32b RTX.png]

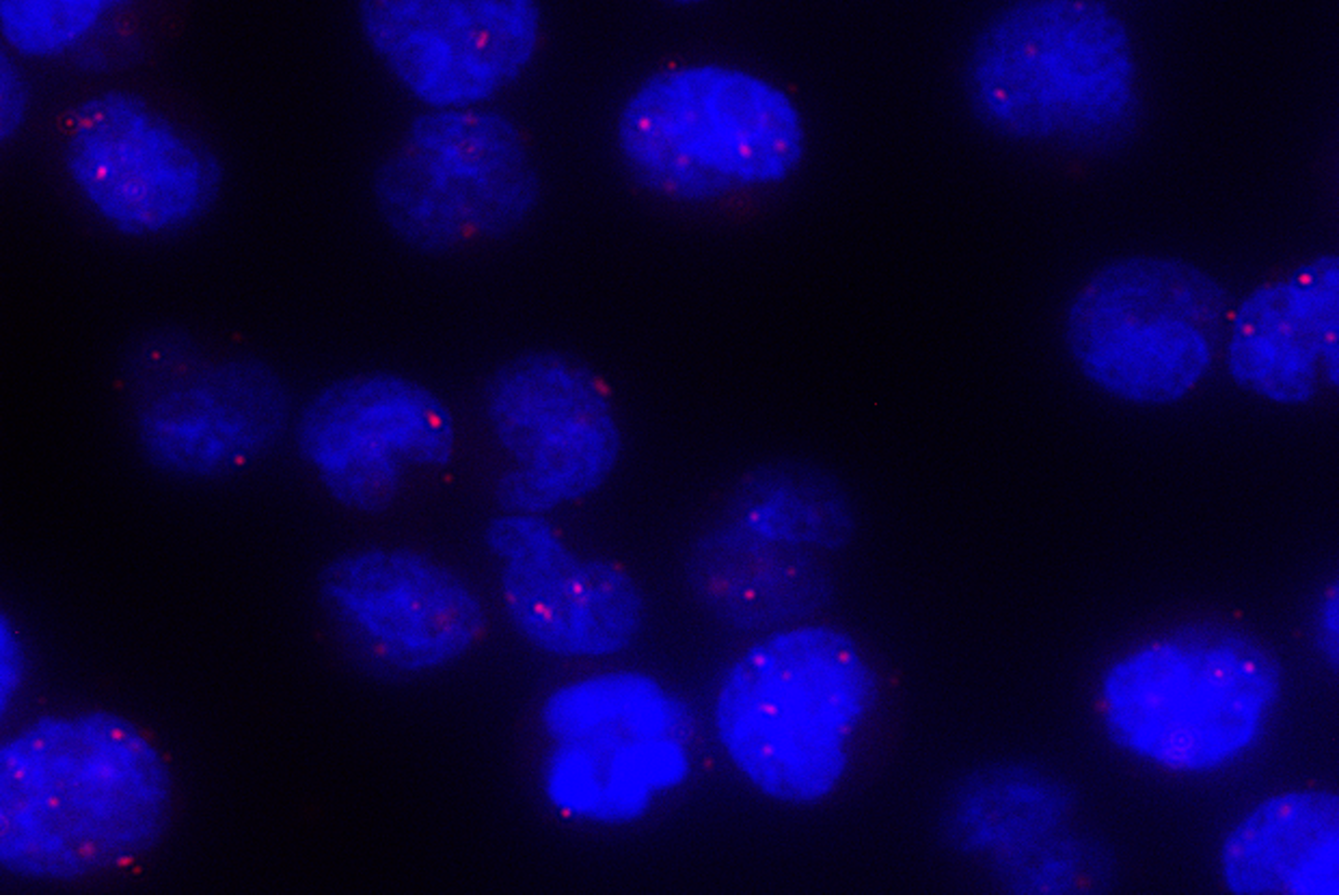

Supplement: Supplementary file 9 — Source data Fig. 5 [file 44318_2026_781_MOESM9_ESM.zip › Figure 5/E/Fig 5E NCmut.png]

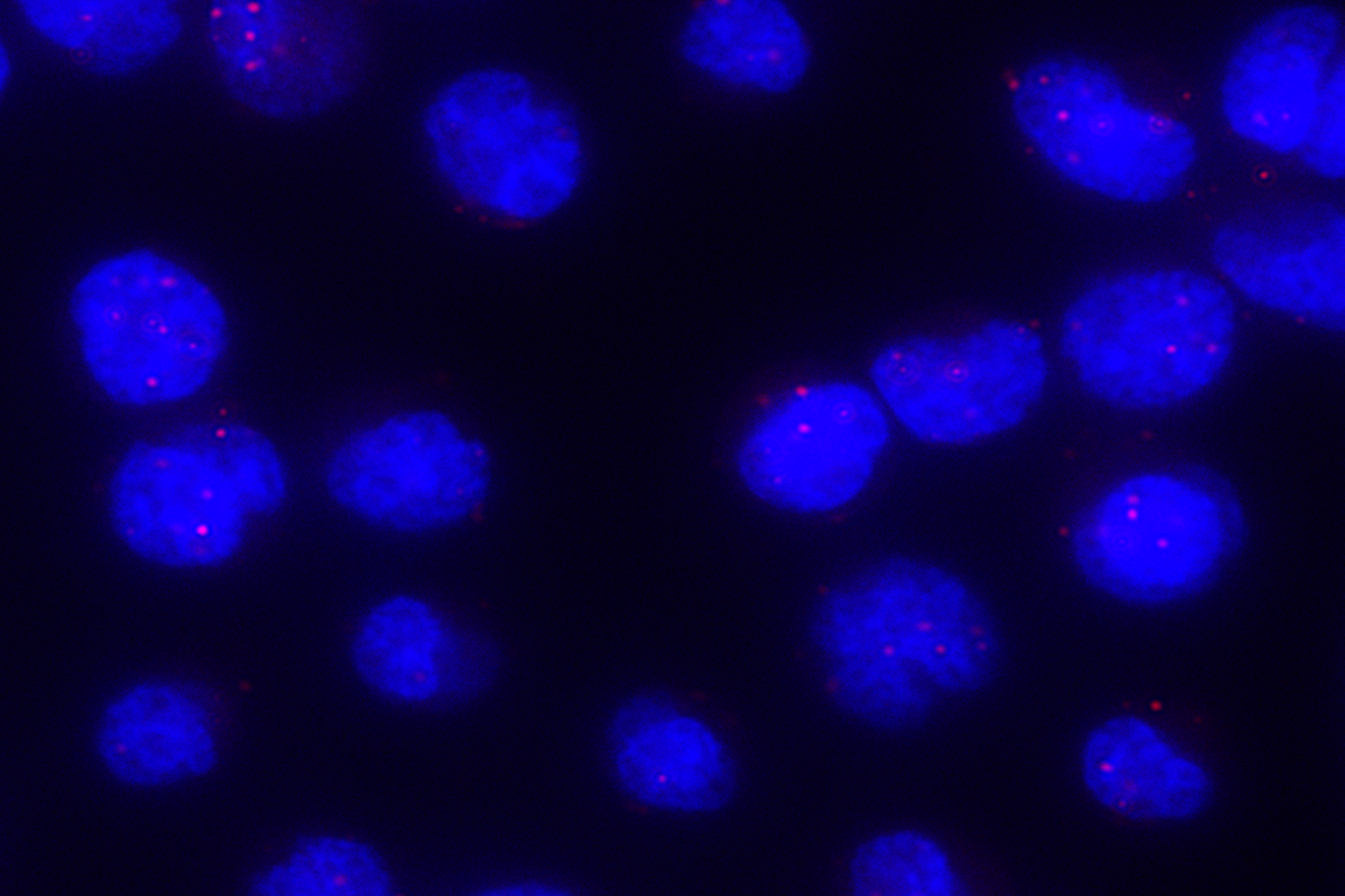

Supplement: Supplementary file 9 — Source data Fig. 5 [file 44318_2026_781_MOESM9_ESM.zip › Figure 5/E/Fig 5E CD32b.png]

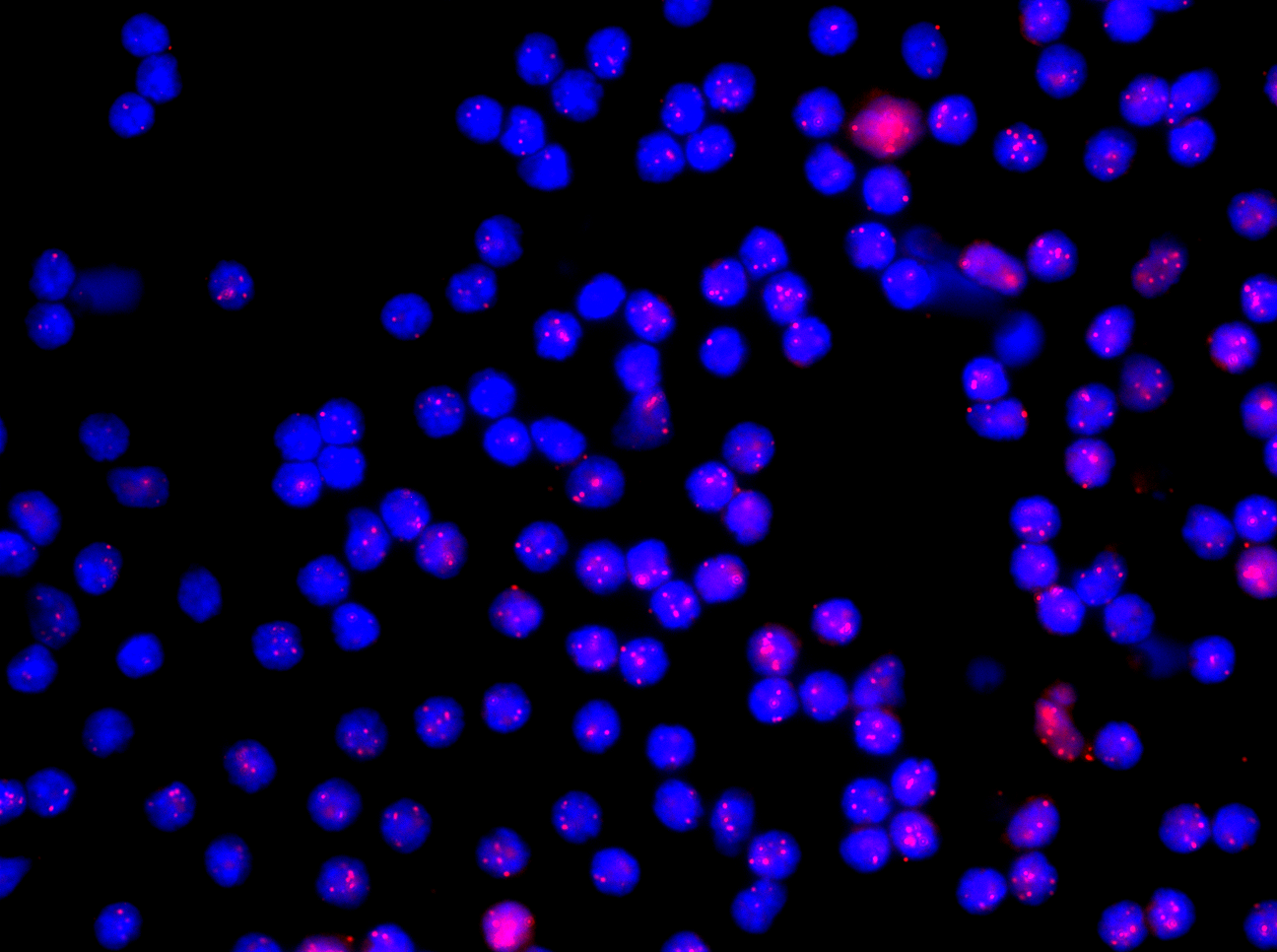

Supplement: Supplementary file 10 — Source data Fig. 6 [file 44318_2026_781_MOESM10_ESM.zip › Figure 6/G/Fig 6G NOC.png]

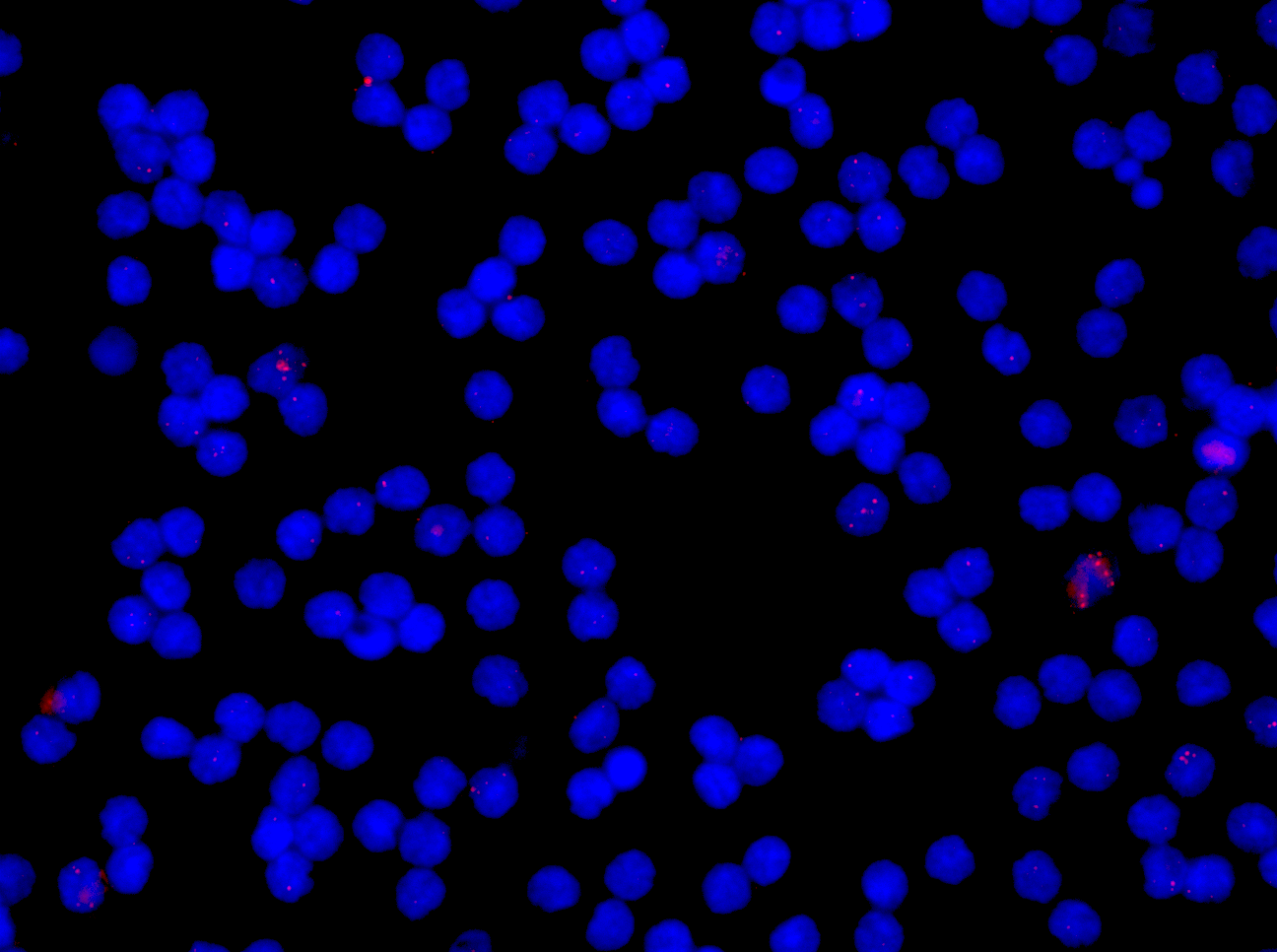

Supplement: Supplementary file 10 — Source data Fig. 6 [file 44318_2026_781_MOESM10_ESM.zip › Figure 6/G/Fig 6G -.png]

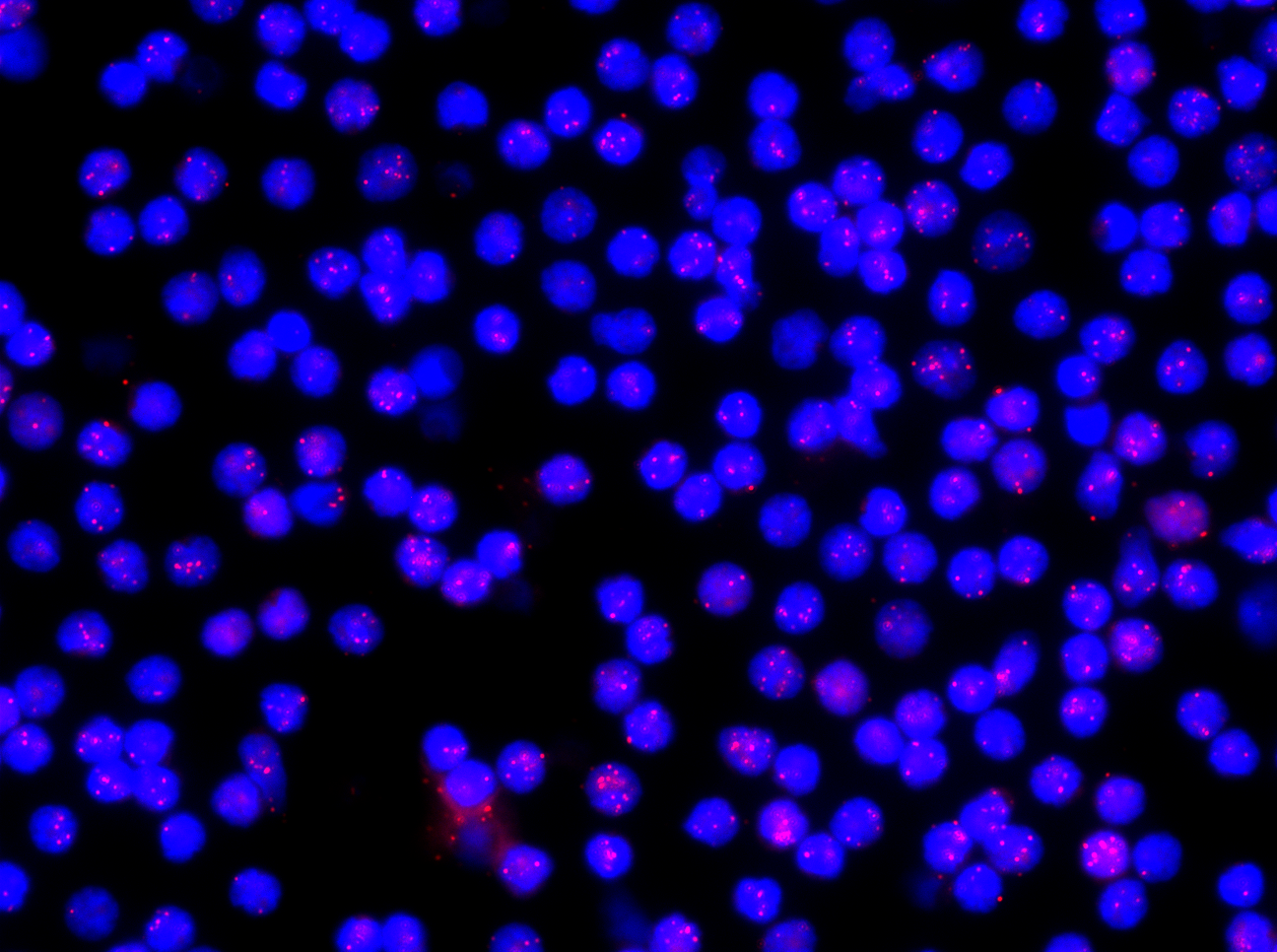

Supplement: Supplementary file 10 — Source data Fig. 6 [file 44318_2026_781_MOESM10_ESM.zip › Figure 6/G/Fig 6G RTX.png]

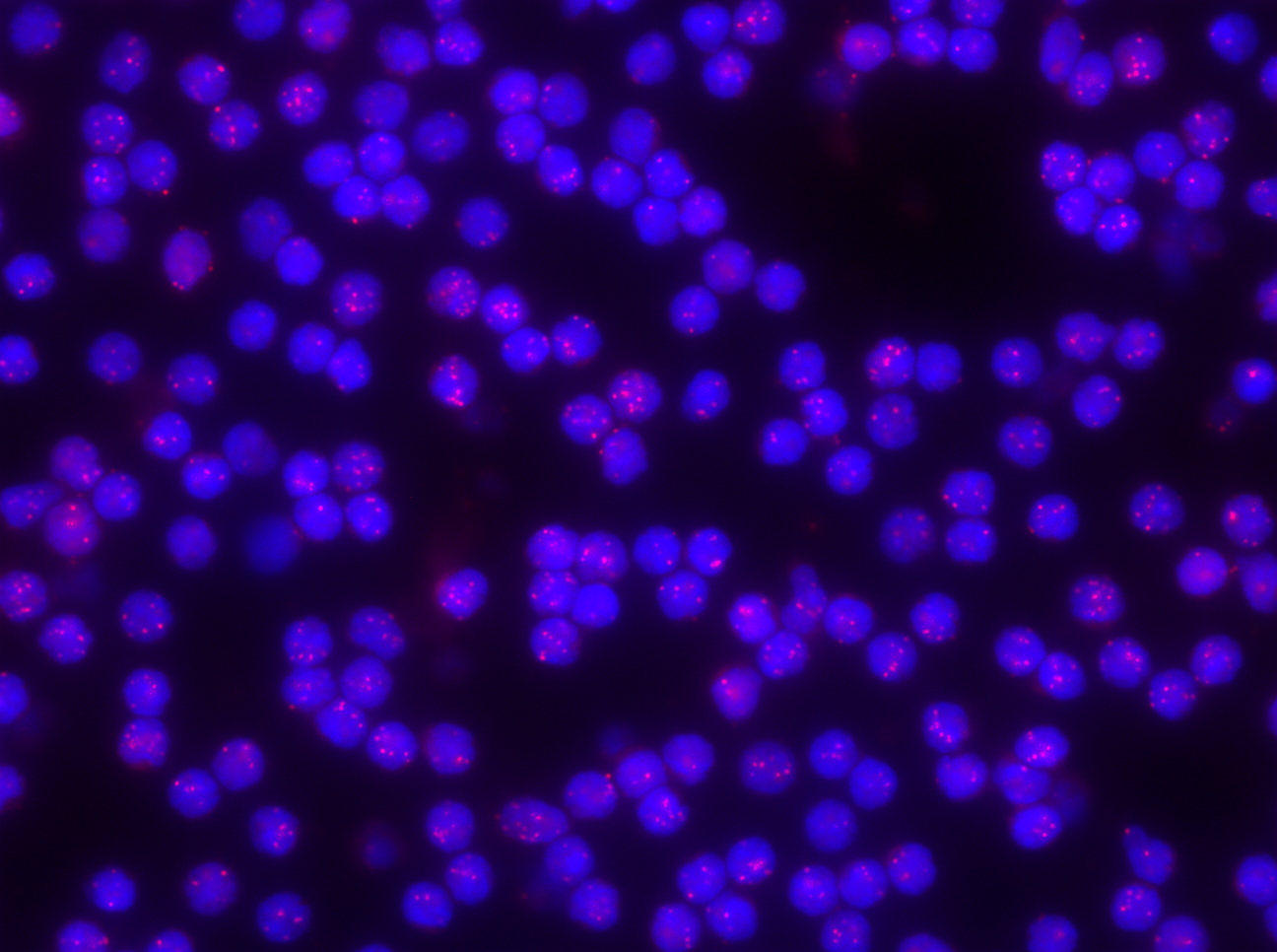

Supplement: Supplementary file 10 — Source data Fig. 6 [file 44318_2026_781_MOESM10_ESM.zip › Figure 6/A/Fig 6A -.png]

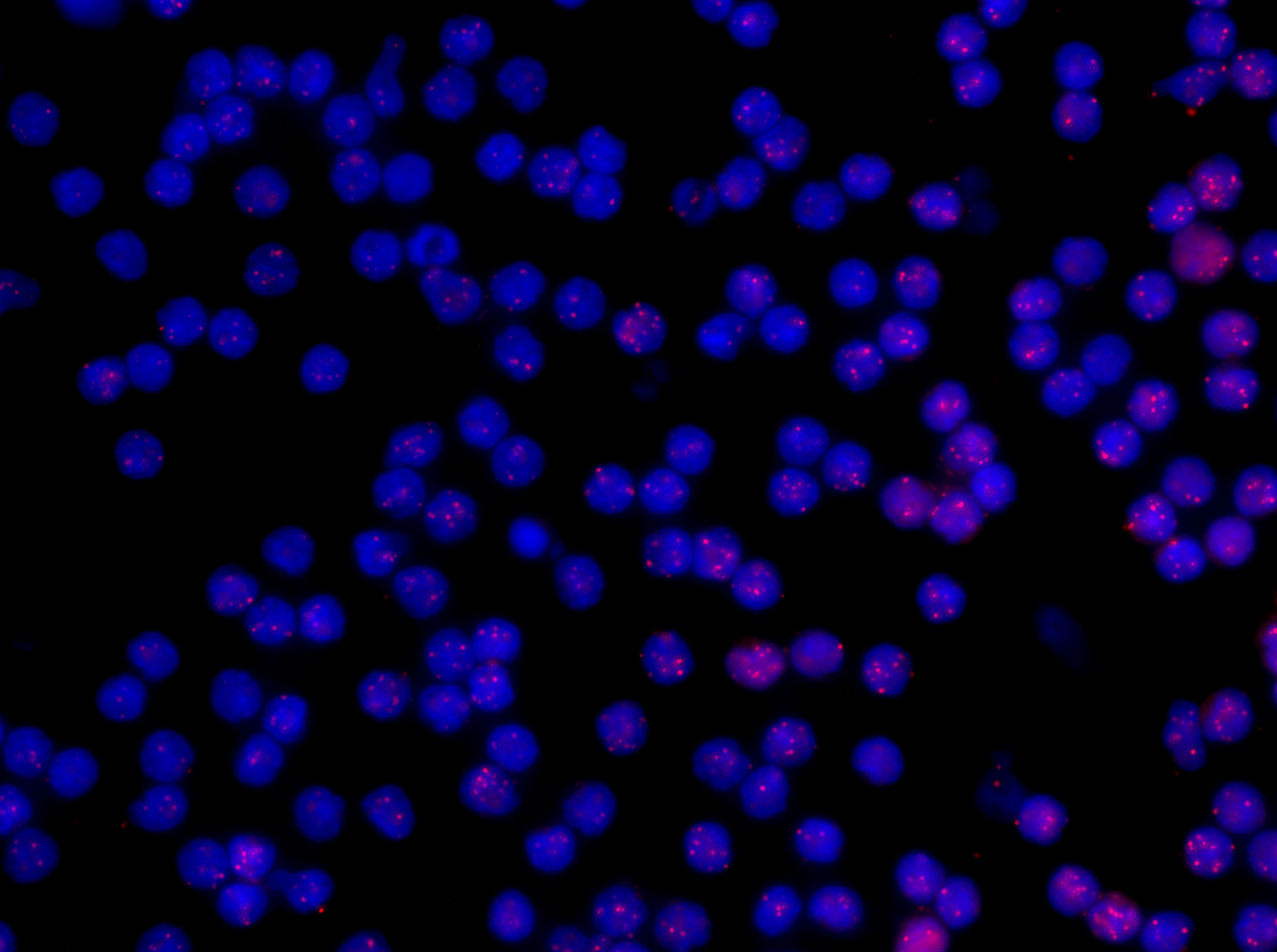

Supplement: Supplementary file 10 — Source data Fig. 6 [file 44318_2026_781_MOESM10_ESM.zip › Figure 6/A/Fig 6A NOC.png]

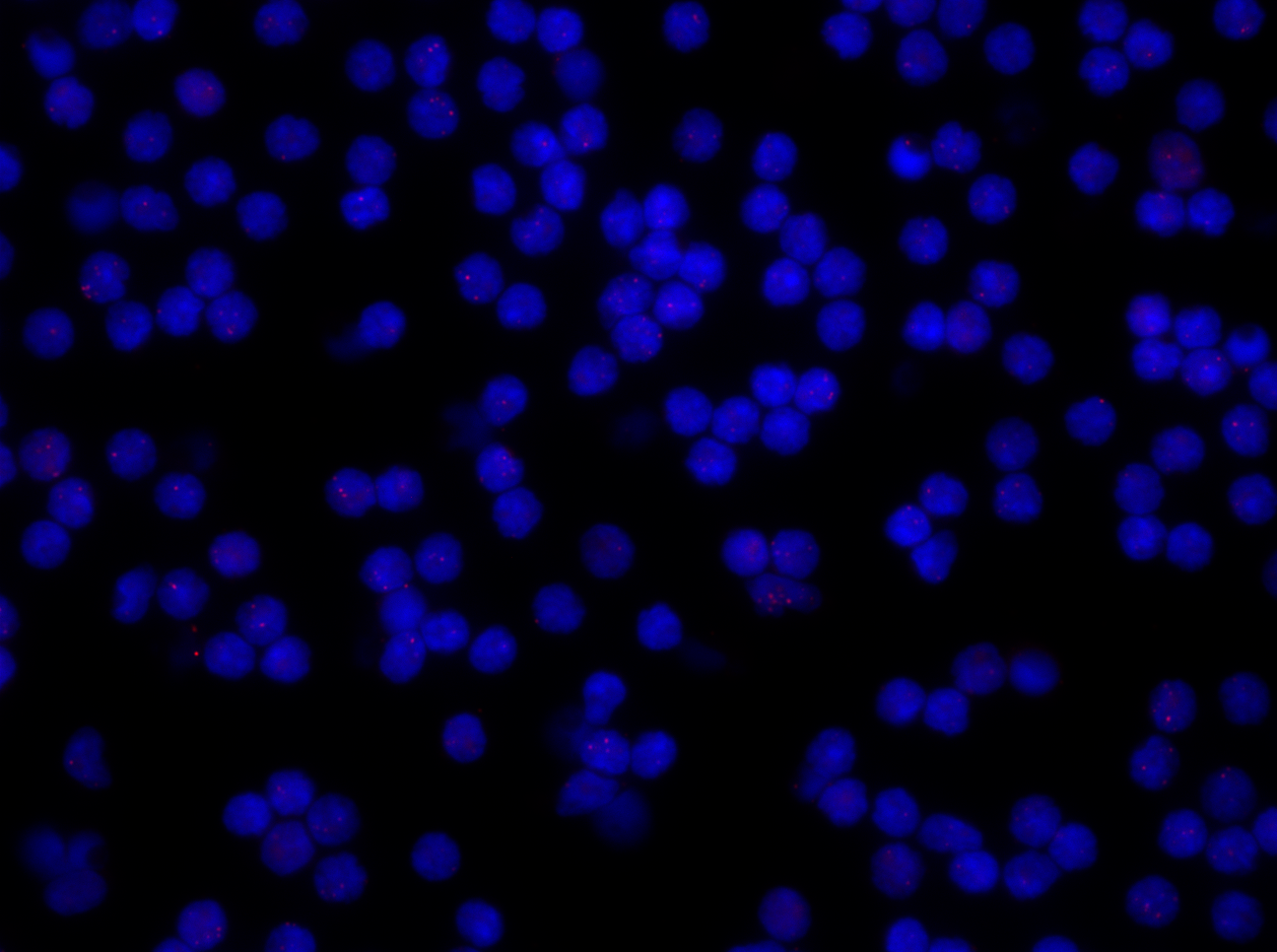

Supplement: Supplementary file 10 — Source data Fig. 6 [file 44318_2026_781_MOESM10_ESM.zip › Figure 6/A/Fig 6A RTX.png]

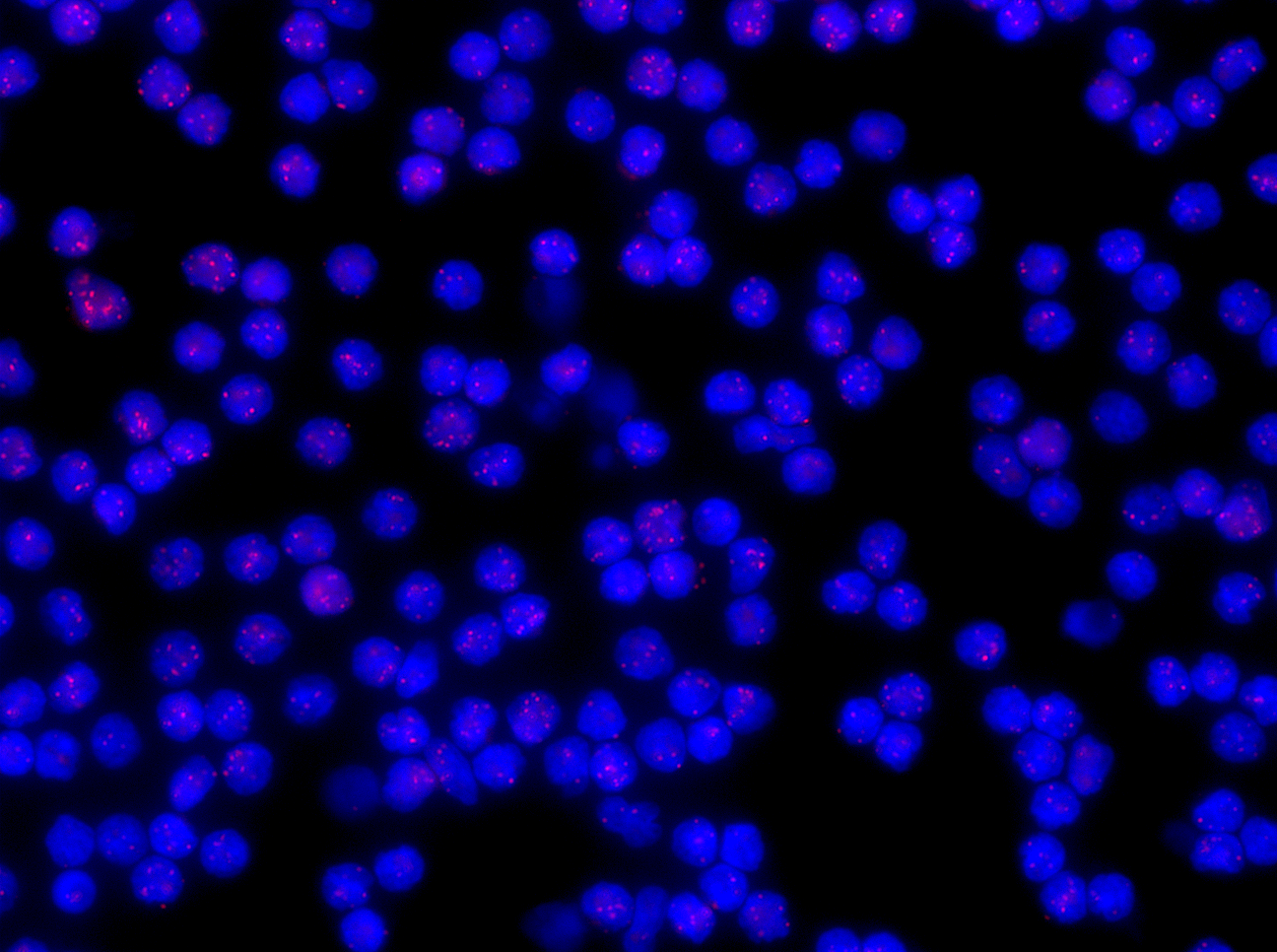

Supplement: Supplementary file 10 — Source data Fig. 6 [file 44318_2026_781_MOESM10_ESM.zip › Figure 6/C/Fig 6C -.png]

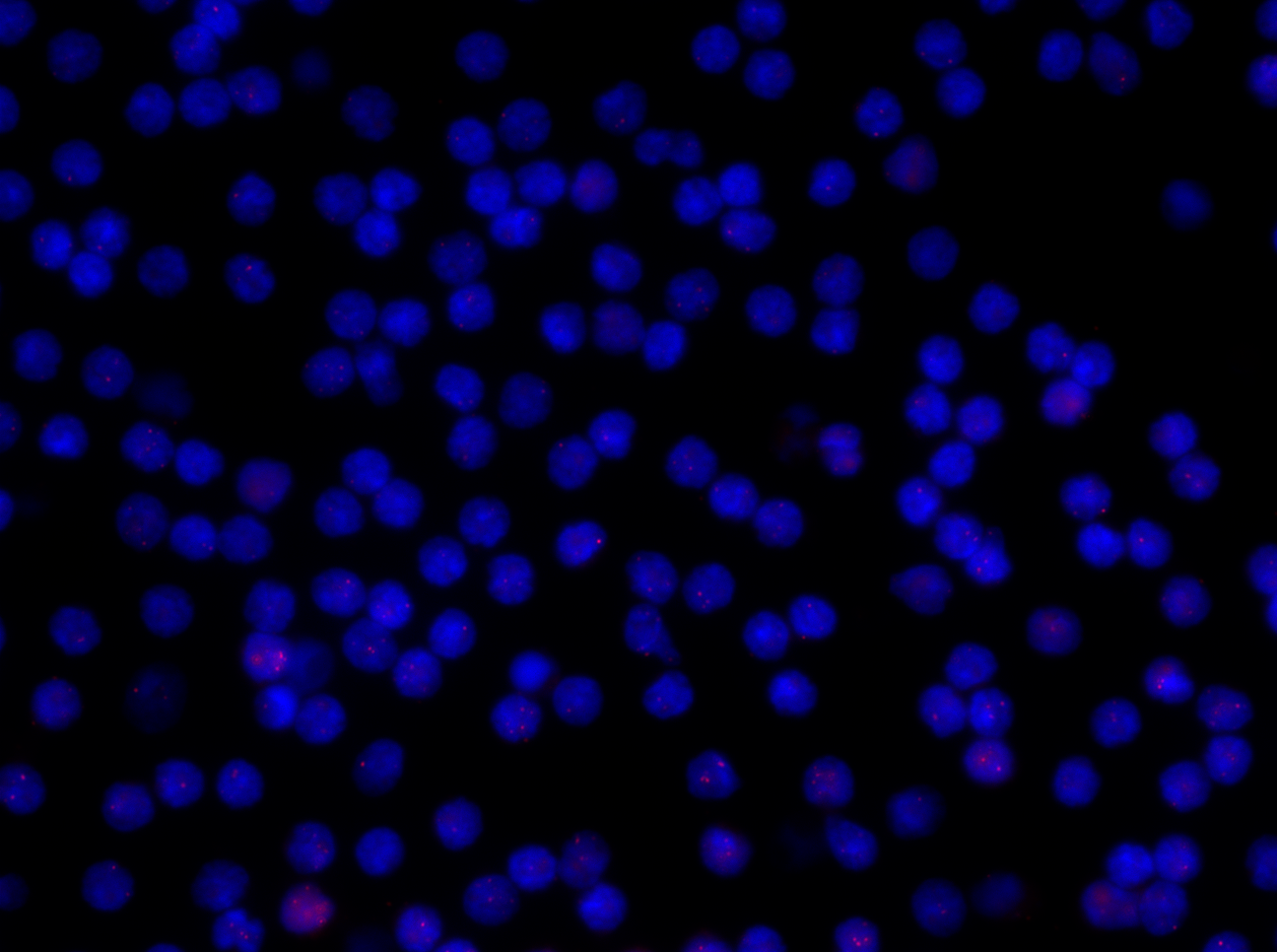

Supplement: Supplementary file 10 — Source data Fig. 6 [file 44318_2026_781_MOESM10_ESM.zip › Figure 6/C/Fig 6C RTX.png]

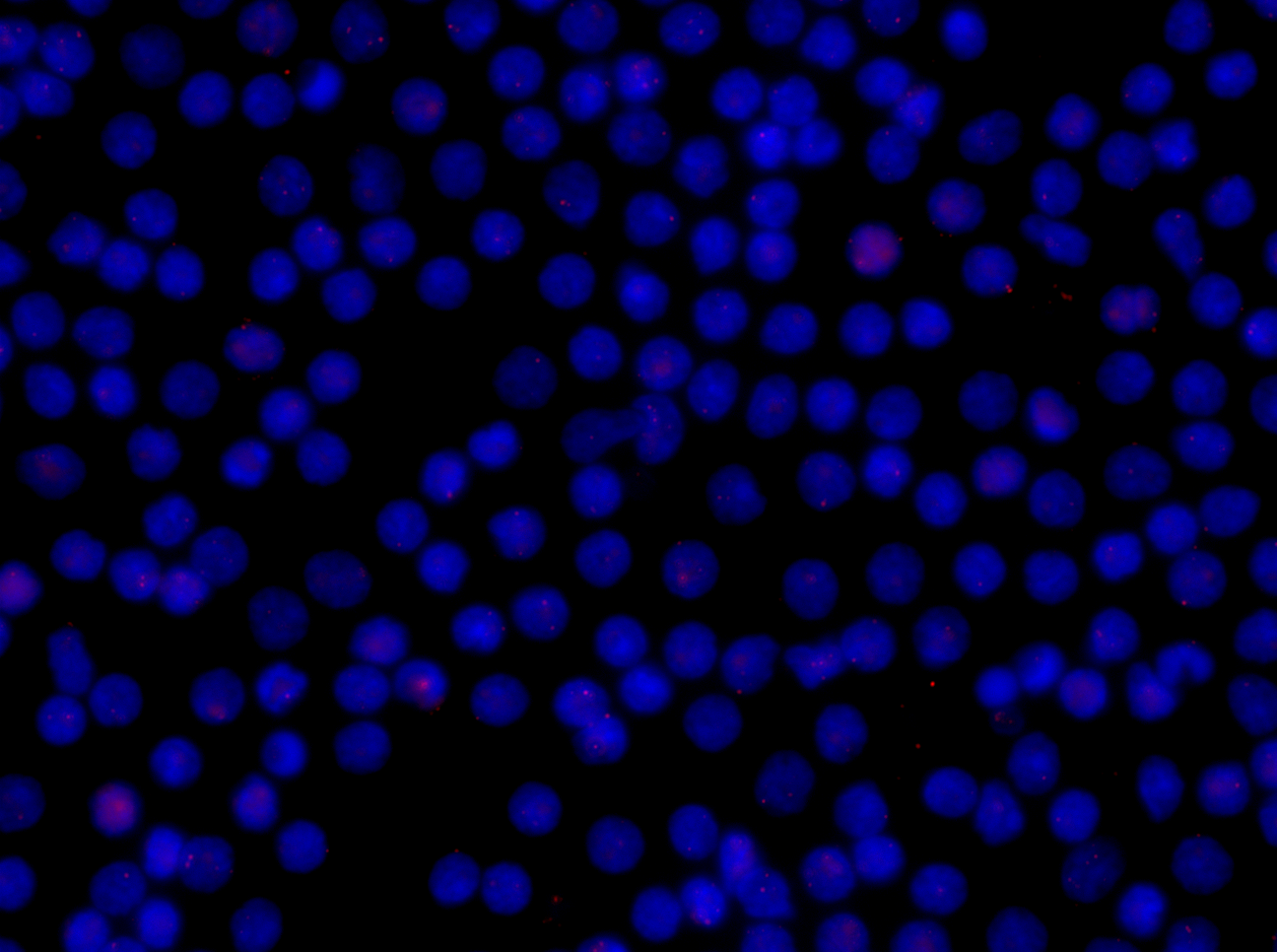

Supplement: Supplementary file 10 — Source data Fig. 6 [file 44318_2026_781_MOESM10_ESM.zip › Figure 6/C/Fig 6C NOC.png]

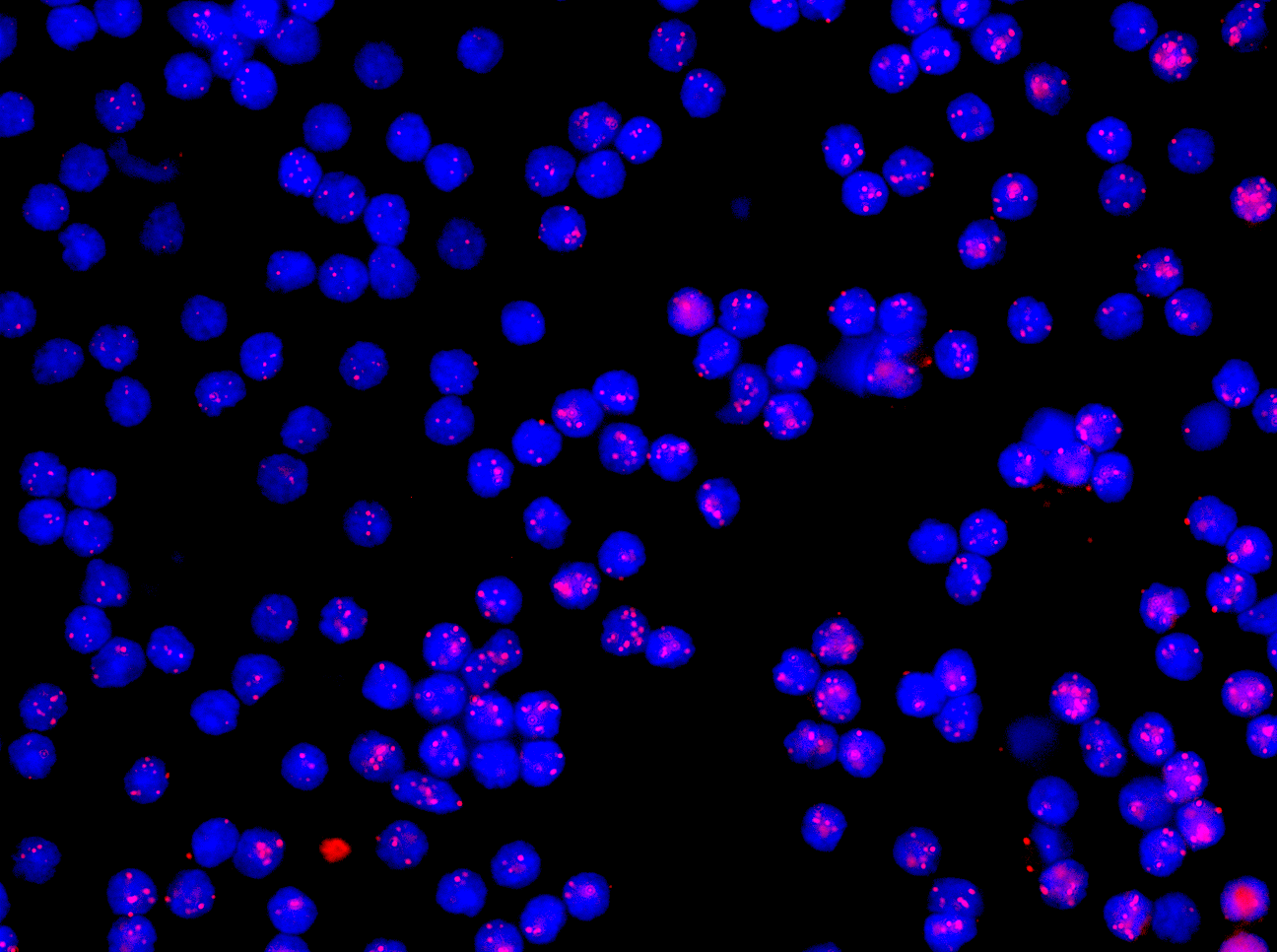

Supplement: Supplementary file 10 — Source data Fig. 6 [file 44318_2026_781_MOESM10_ESM.zip › Figure 6/E/Fig 6E RTX.png]

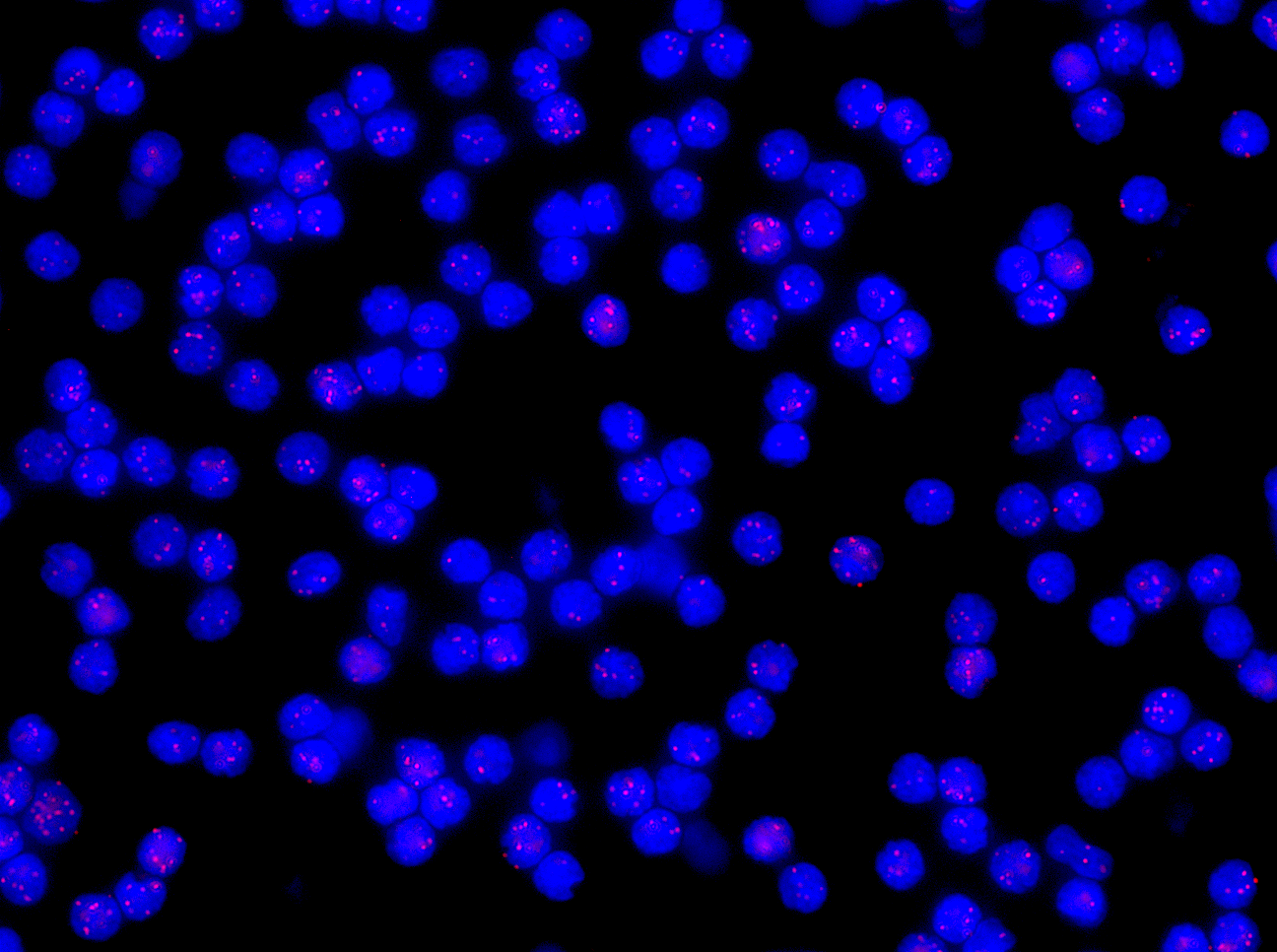

Supplement: Supplementary file 10 — Source data Fig. 6 [file 44318_2026_781_MOESM10_ESM.zip › Figure 6/E/Fig 6E NOC.png]

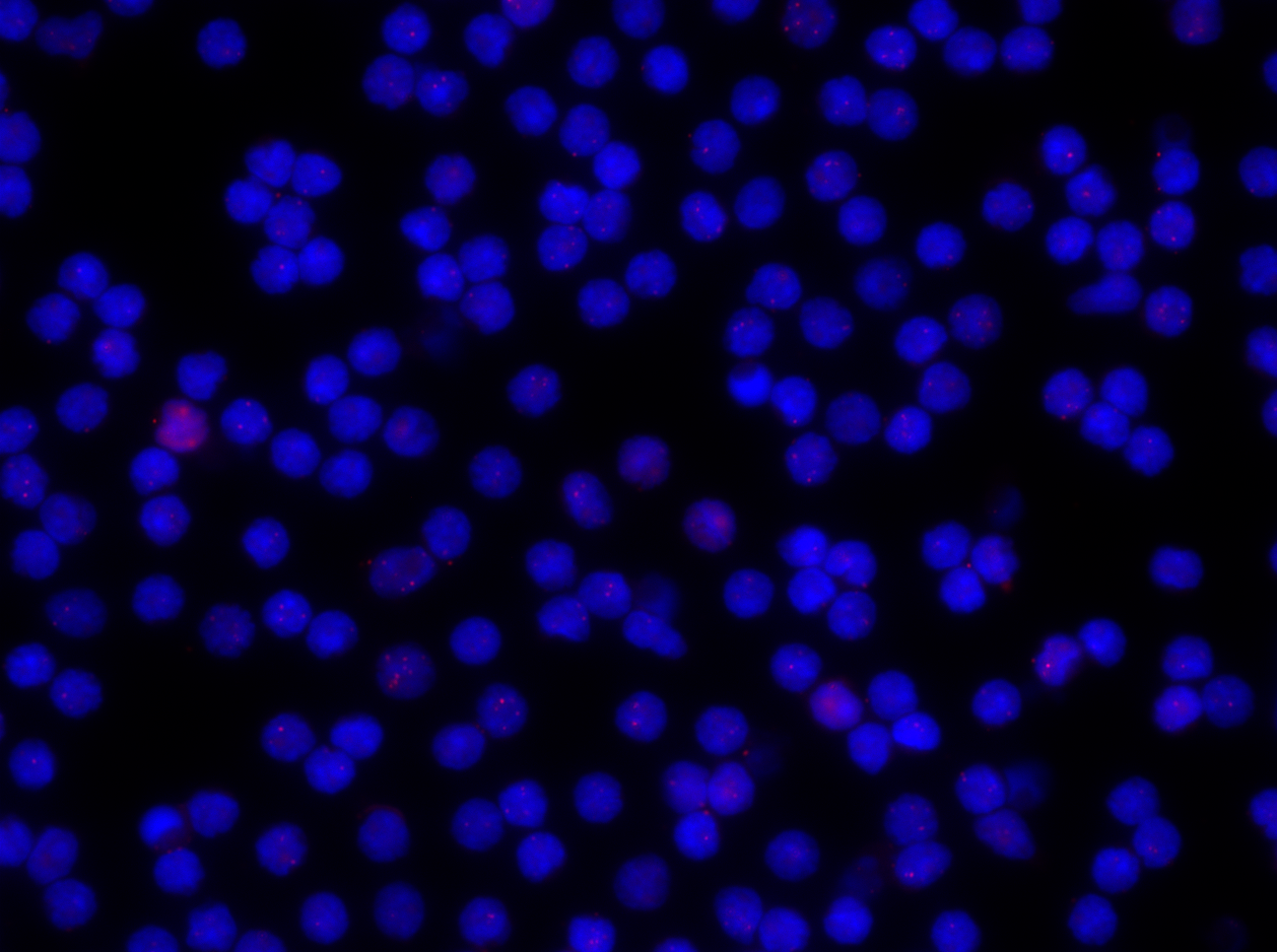

Supplement: Supplementary file 10 — Source data Fig. 6 [file 44318_2026_781_MOESM10_ESM.zip › Figure 6/E/Fig 6E 1 -.png]

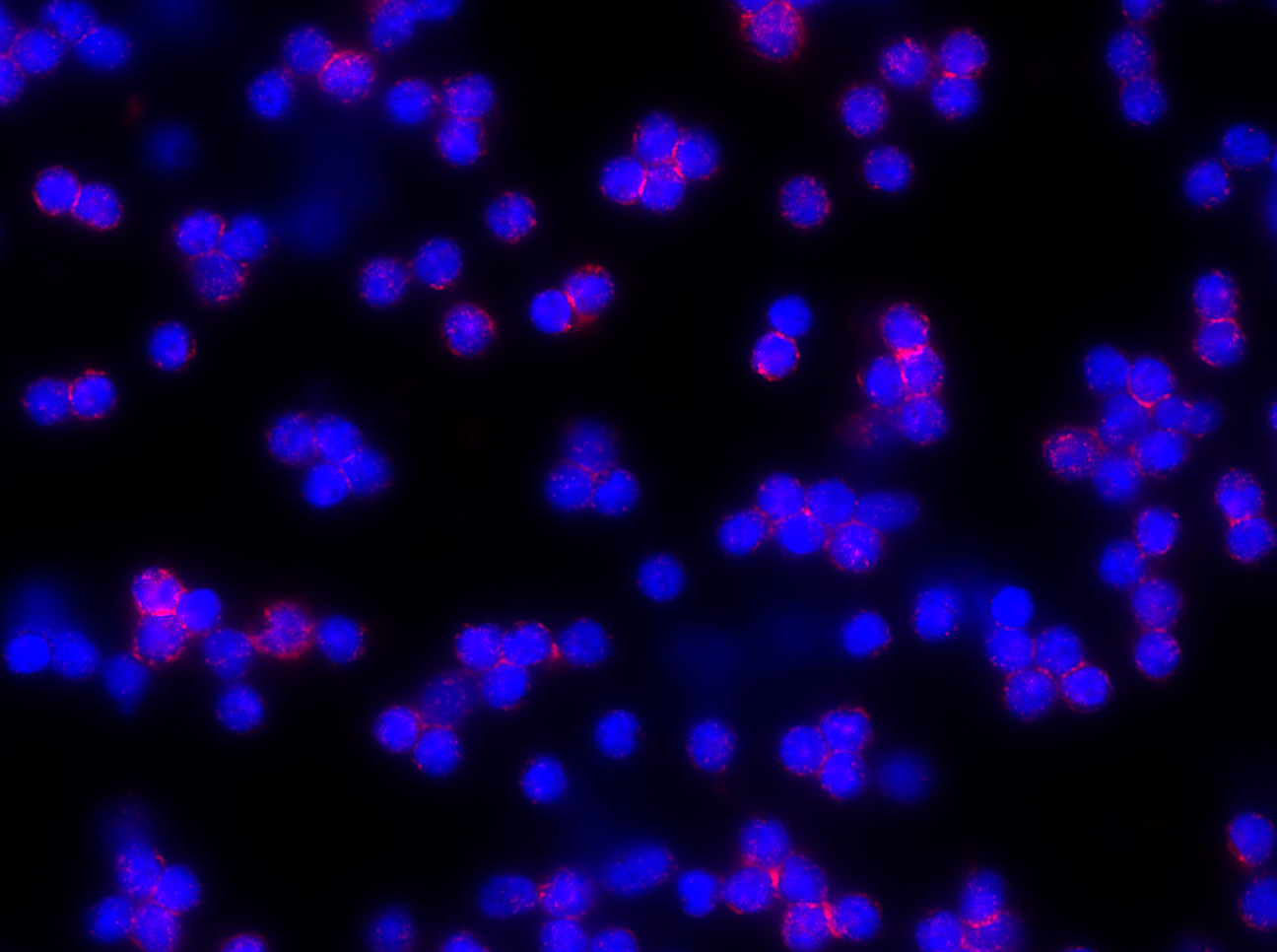

Supplement: Supplementary file 11 — Source data Fig. 7 [file 44318_2026_781_MOESM11_ESM.zip › Figure 7/C/Fig 7C Tax+RTX.png]

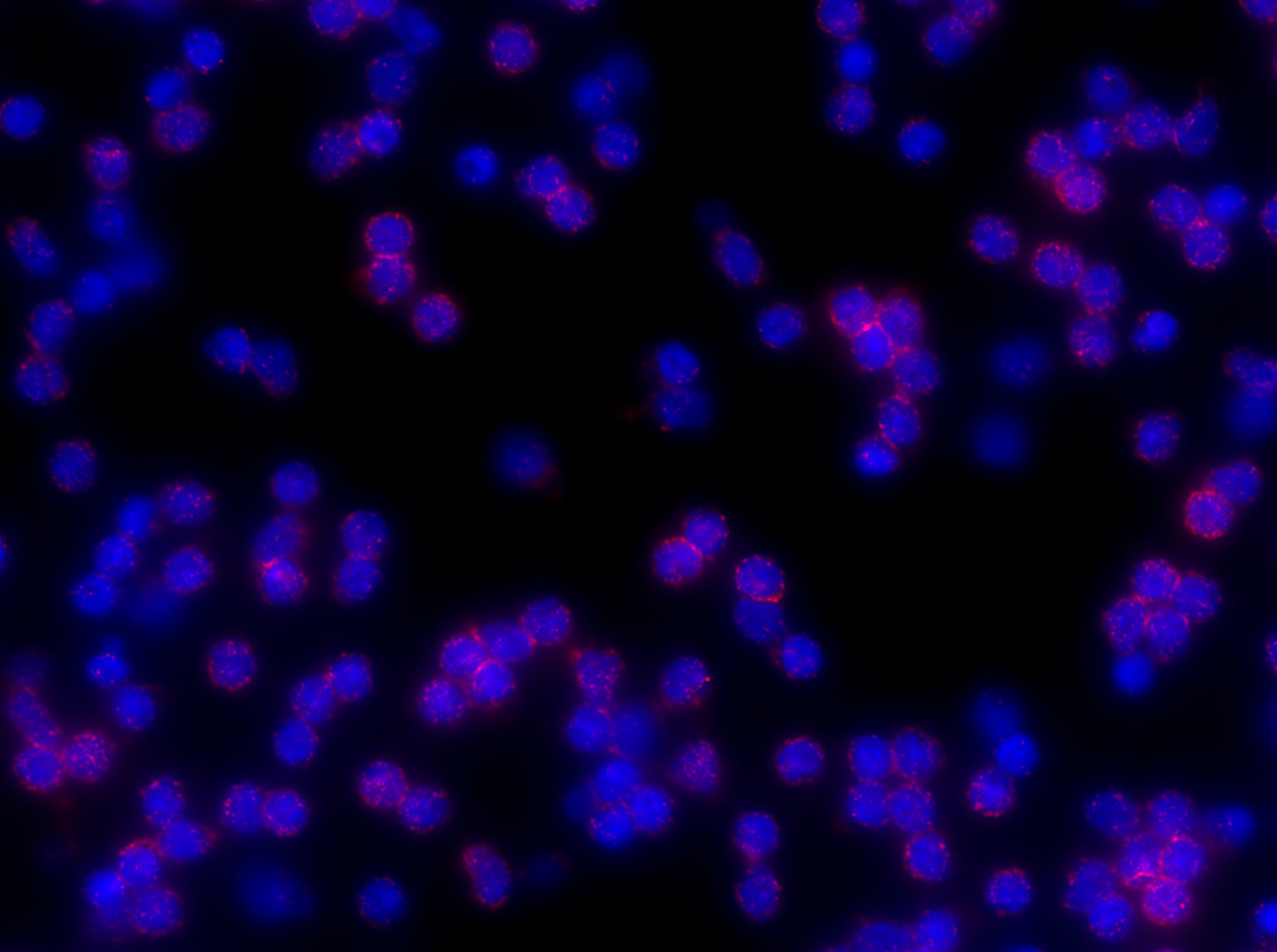

Supplement: Supplementary file 11 — Source data Fig. 7 [file 44318_2026_781_MOESM11_ESM.zip › Figure 7/C/Fig 7C WT.png]

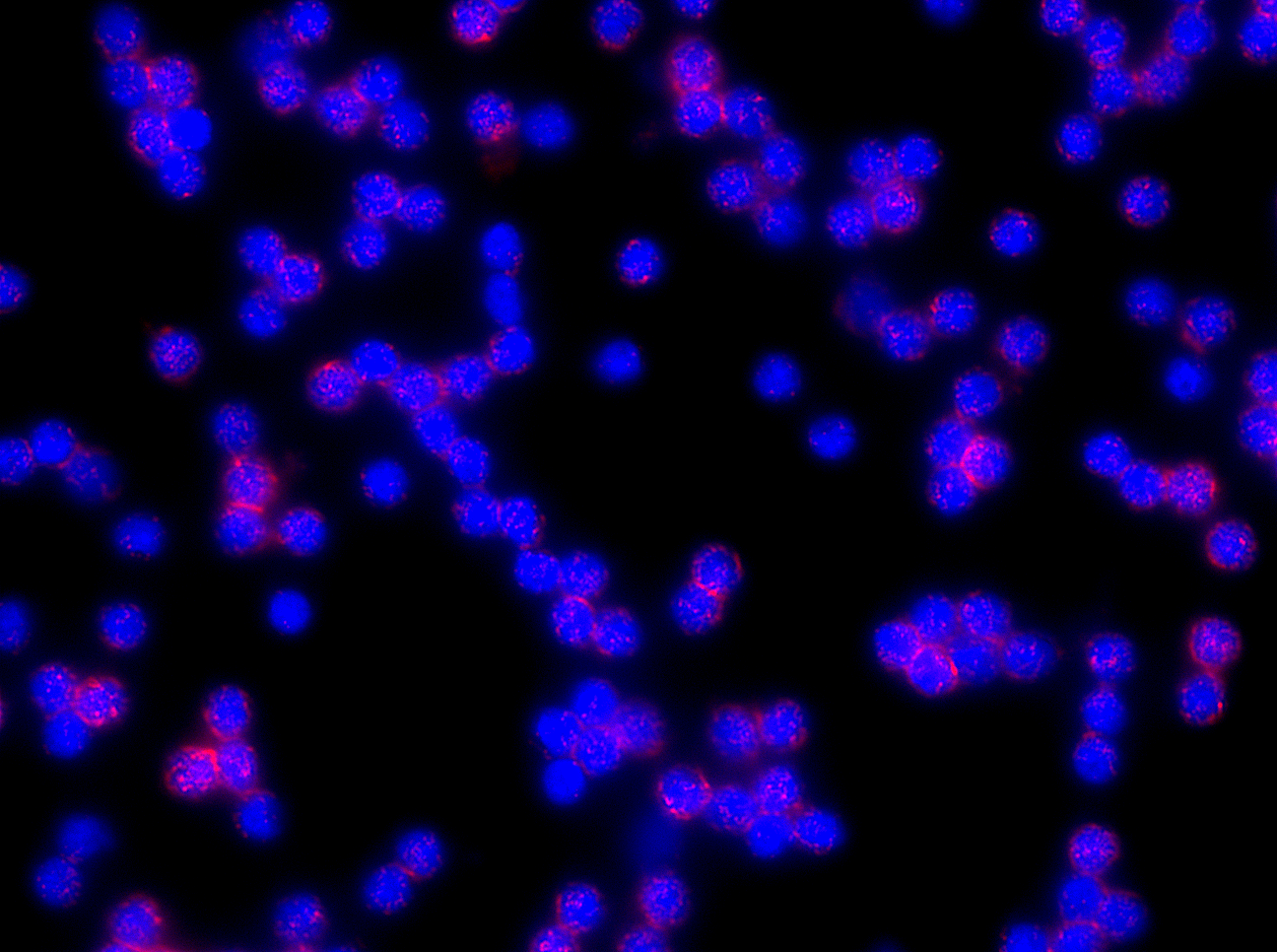

Supplement: Supplementary file 11 — Source data Fig. 7 [file 44318_2026_781_MOESM11_ESM.zip › Figure 7/C/Fig 7C Tax.png]

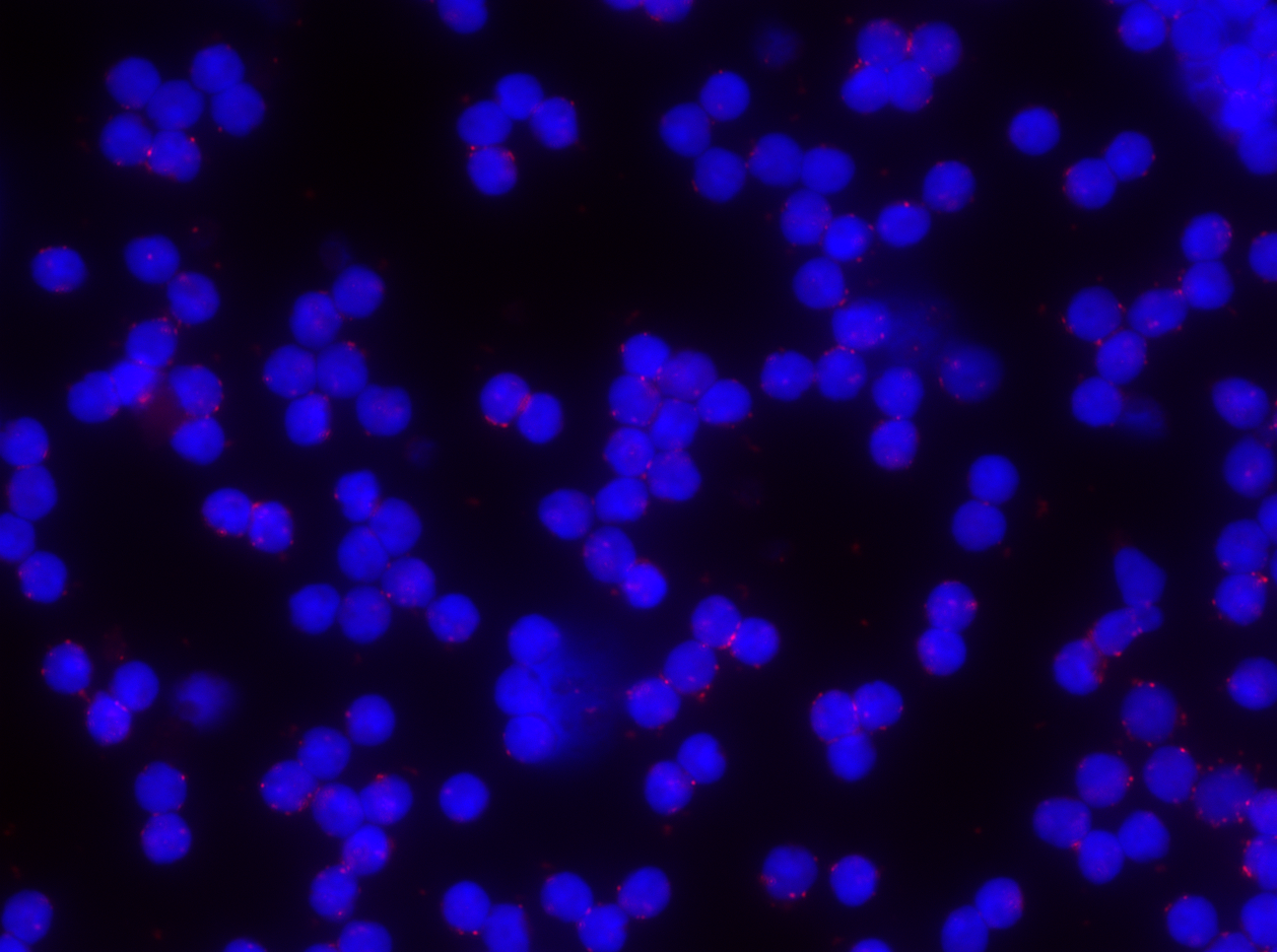

Supplement: Supplementary file 11 — Source data Fig. 7 [file 44318_2026_781_MOESM11_ESM.zip › Figure 7/C/Fig 7C RTX.png]

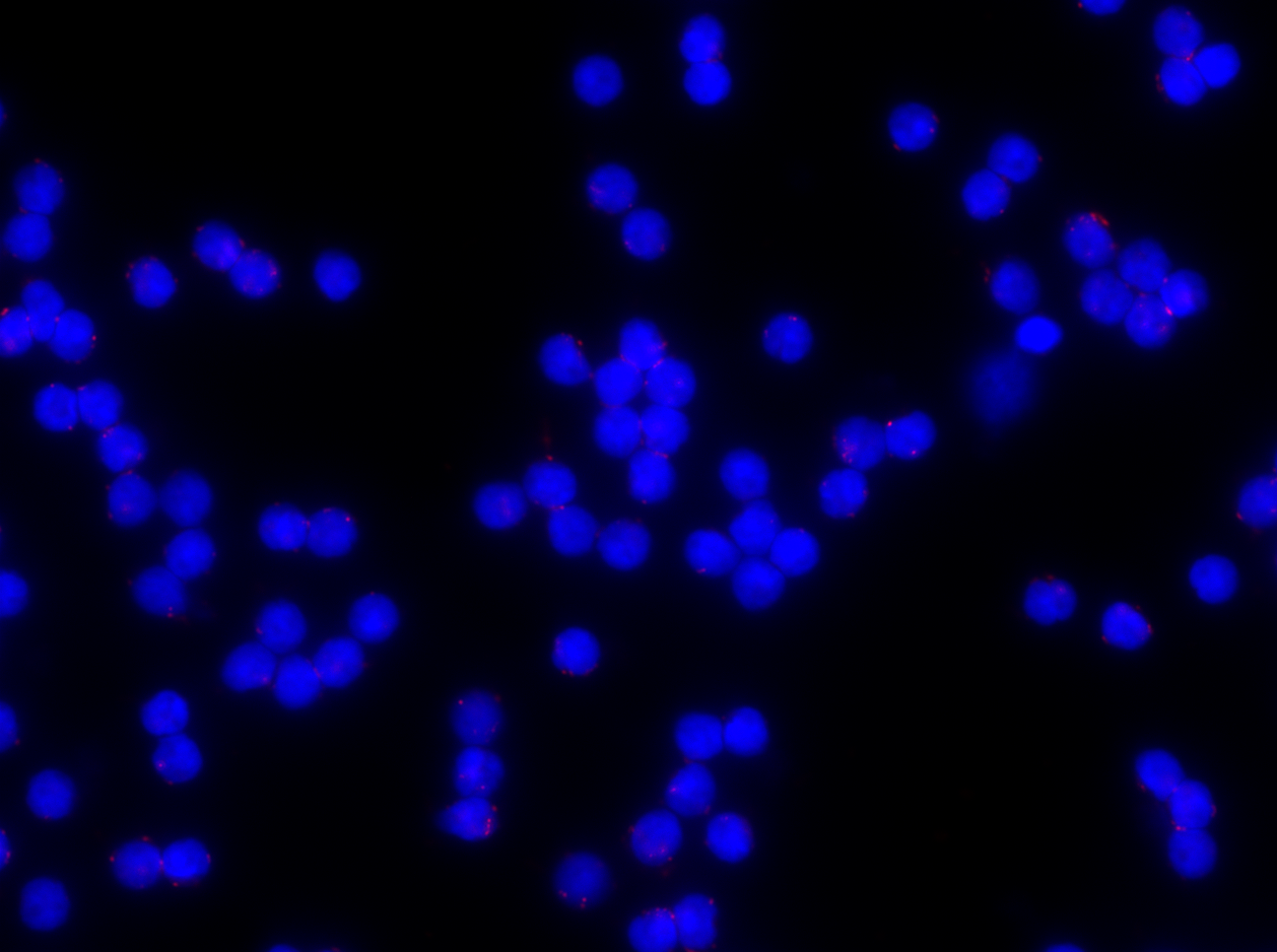

Supplement: Supplementary file 11 — Source data Fig. 7 [file 44318_2026_781_MOESM11_ESM.zip › Figure 7/C/Fig 7C Noc+RTX.png]

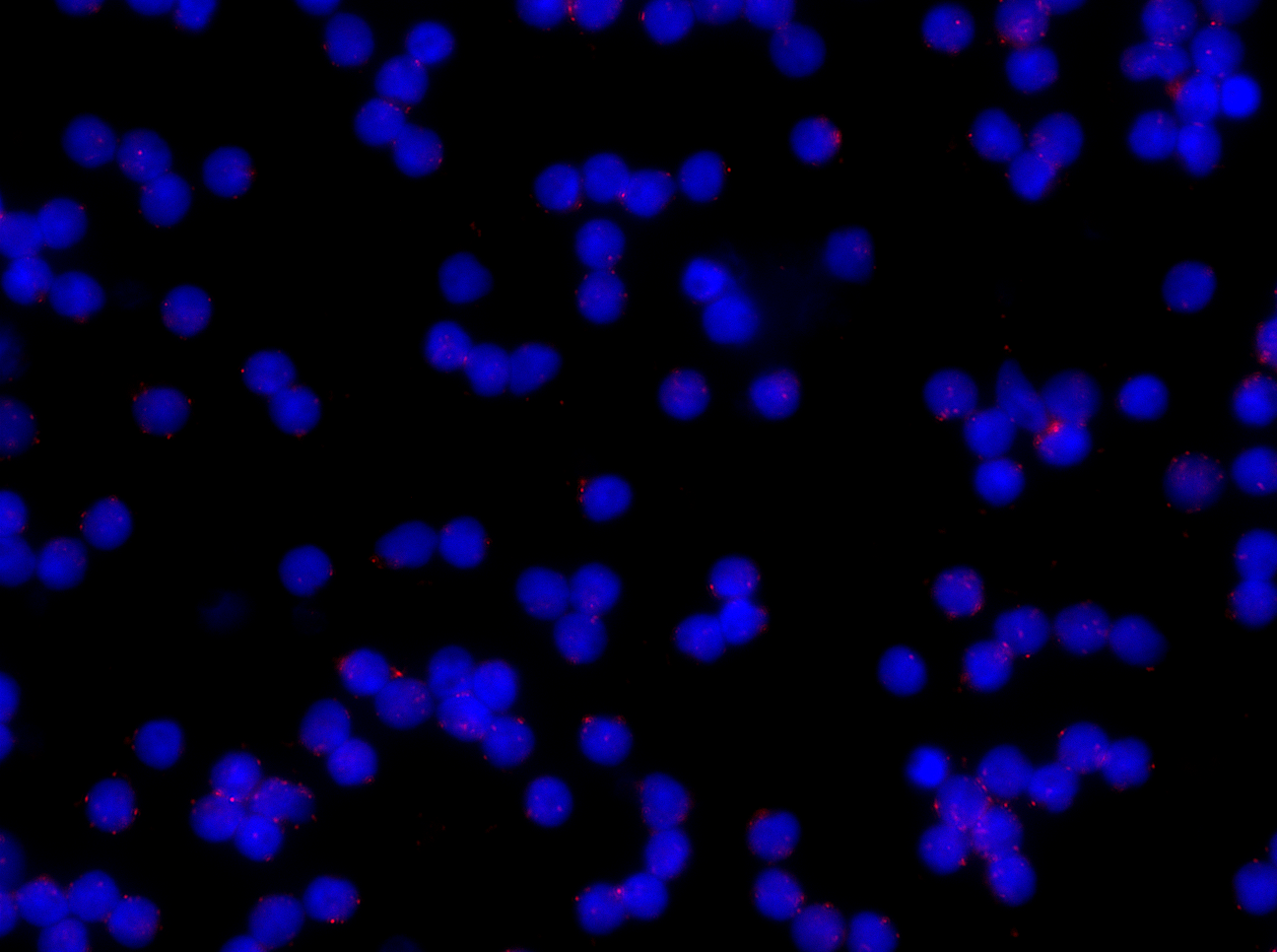

Supplement: Supplementary file 11 — Source data Fig. 7 [file 44318_2026_781_MOESM11_ESM.zip › Figure 7/C/Fig 7C NOC.png]

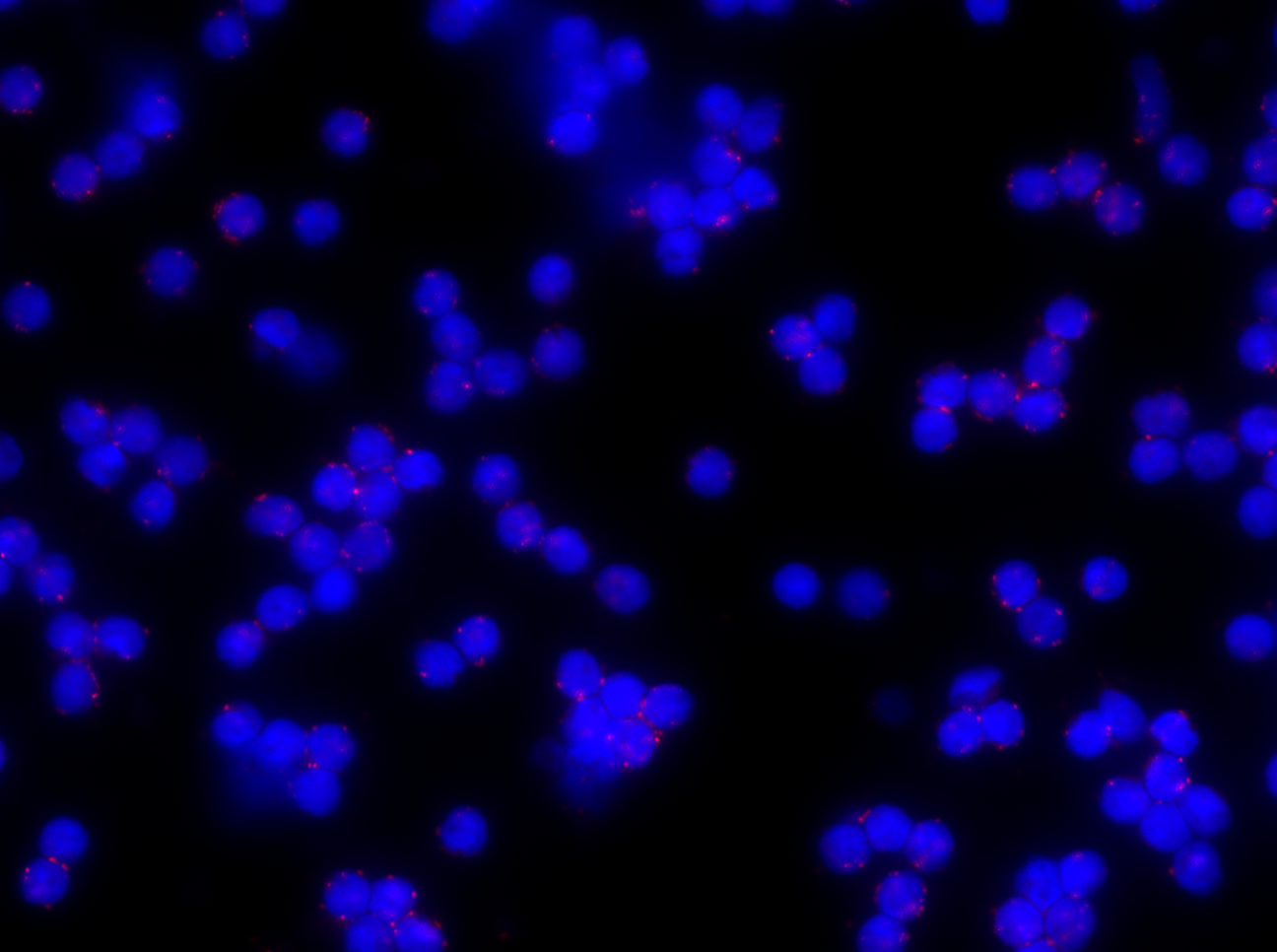

Supplement: Supplementary file 11 — Source data Fig. 7 [file 44318_2026_781_MOESM11_ESM.zip › Figure 7/D/Fig 7D Tax.png]

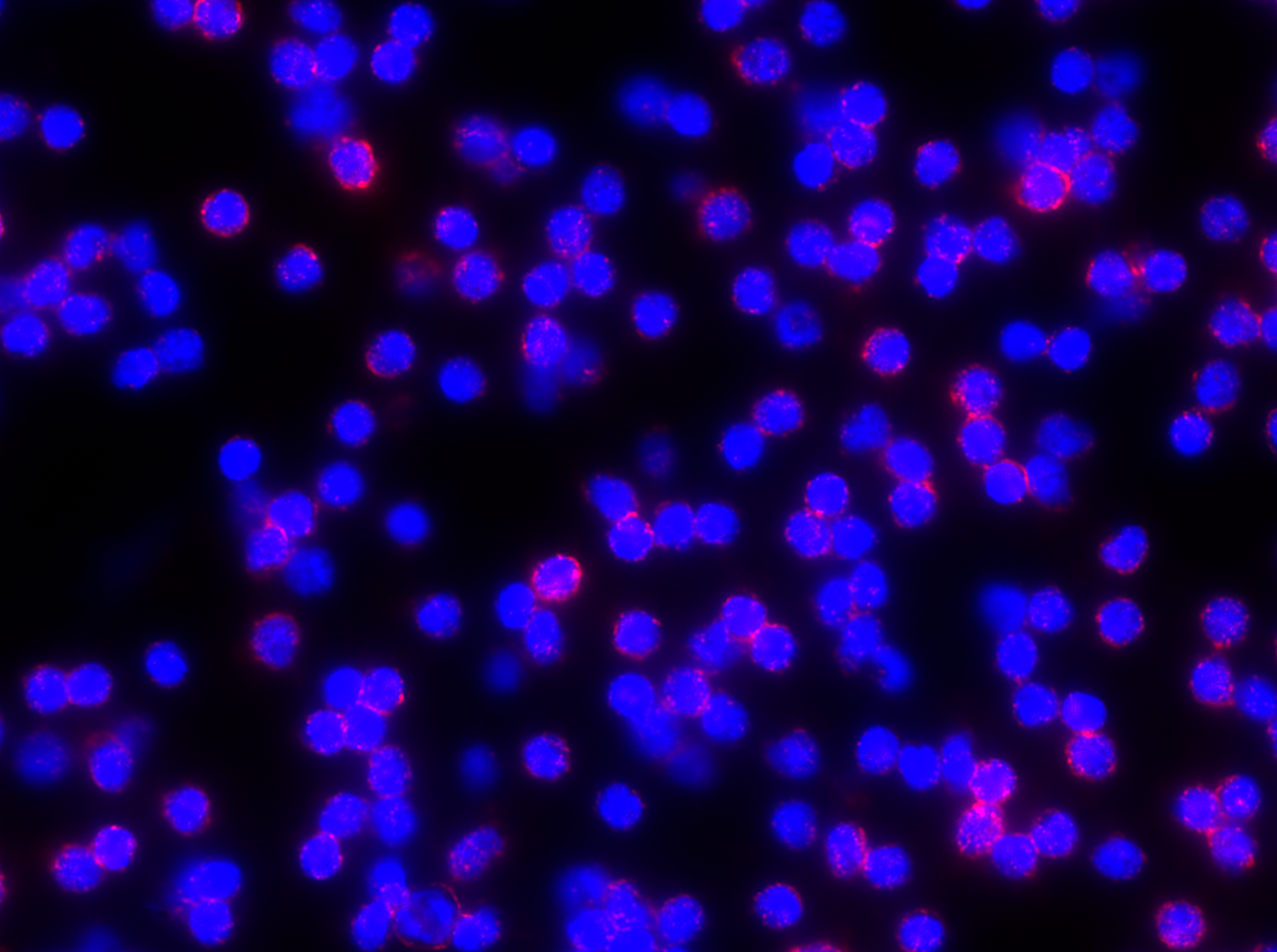

Supplement: Supplementary file 11 — Source data Fig. 7 [file 44318_2026_781_MOESM11_ESM.zip › Figure 7/D/Fig 7D RTX.png]

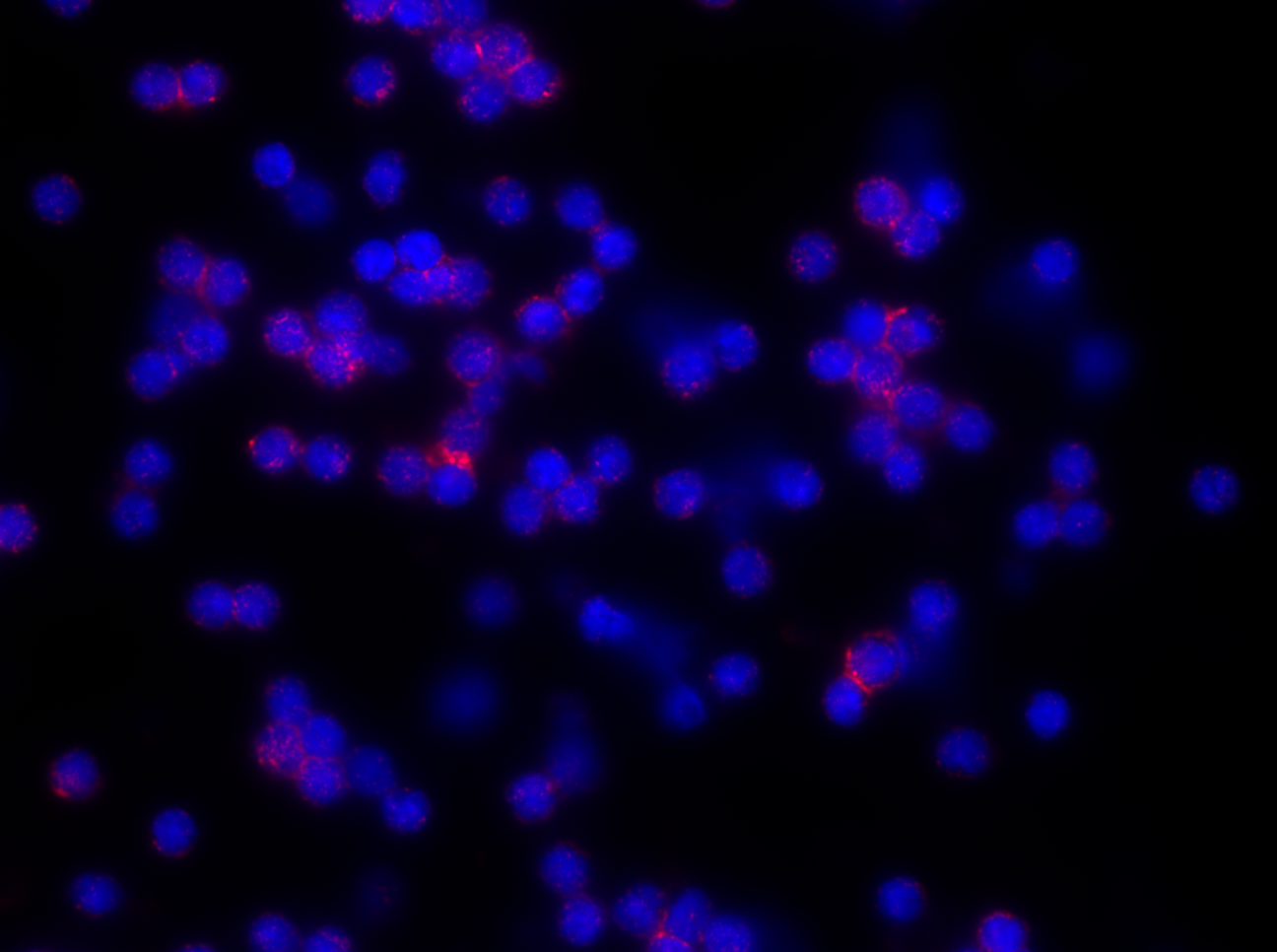

Supplement: Supplementary file 11 — Source data Fig. 7 [file 44318_2026_781_MOESM11_ESM.zip › Figure 7/D/Fig 7D NOC+RTX.png]

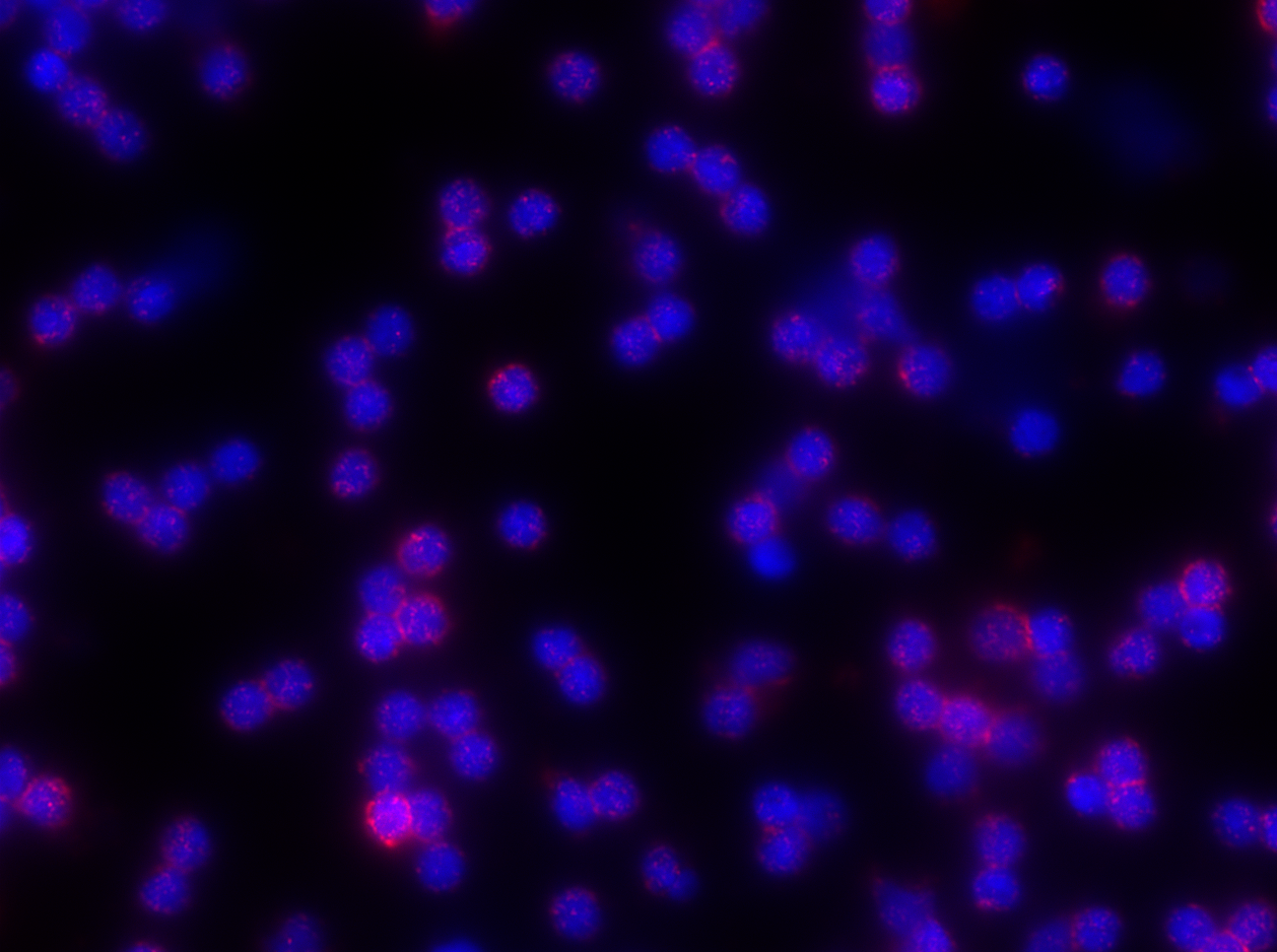

Supplement: Supplementary file 11 — Source data Fig. 7 [file 44318_2026_781_MOESM11_ESM.zip › Figure 7/D/Fig 7D Noc.png]

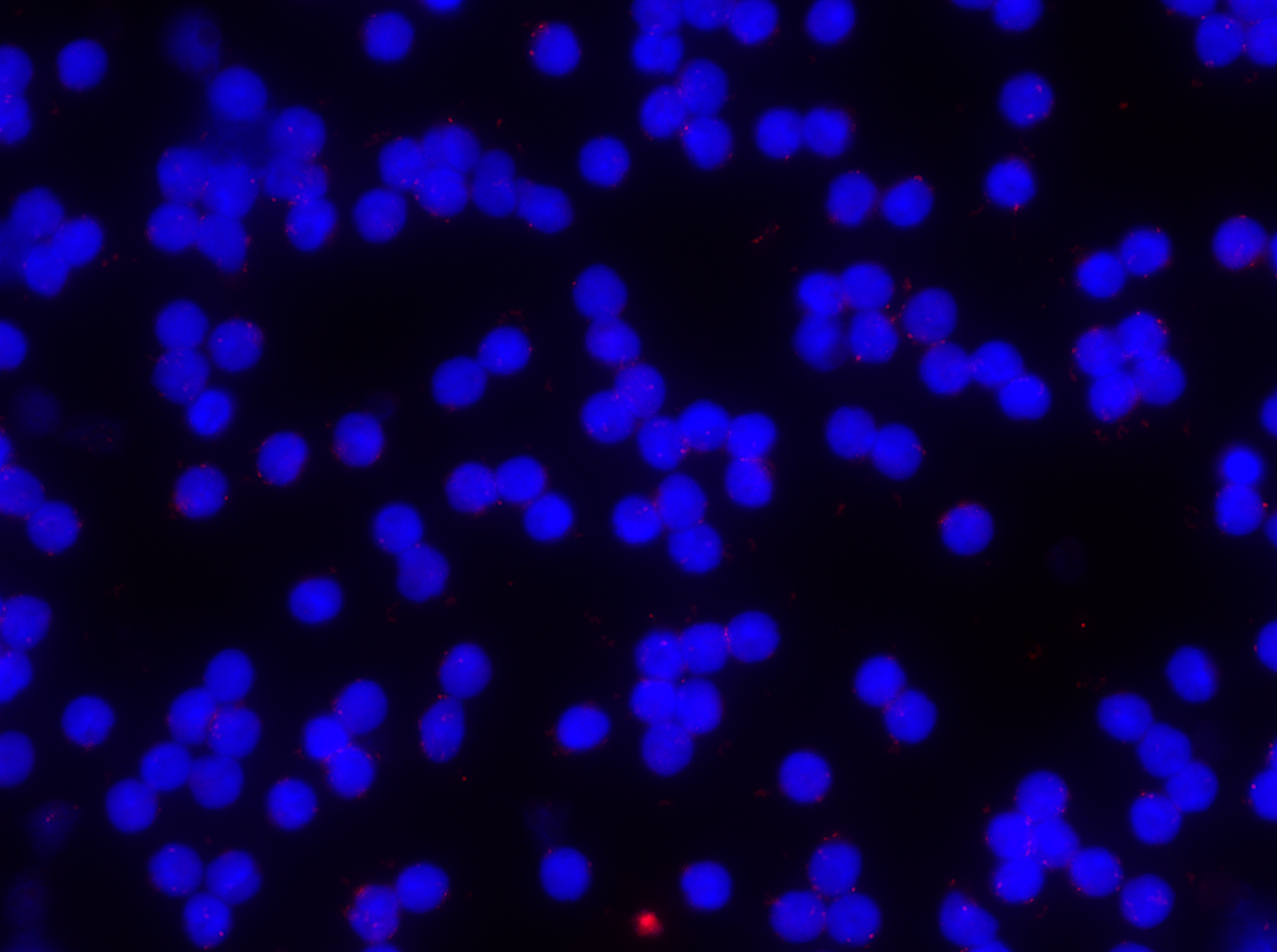

Supplement: Supplementary file 11 — Source data Fig. 7 [file 44318_2026_781_MOESM11_ESM.zip › Figure 7/D/Fig 7D WT.png]

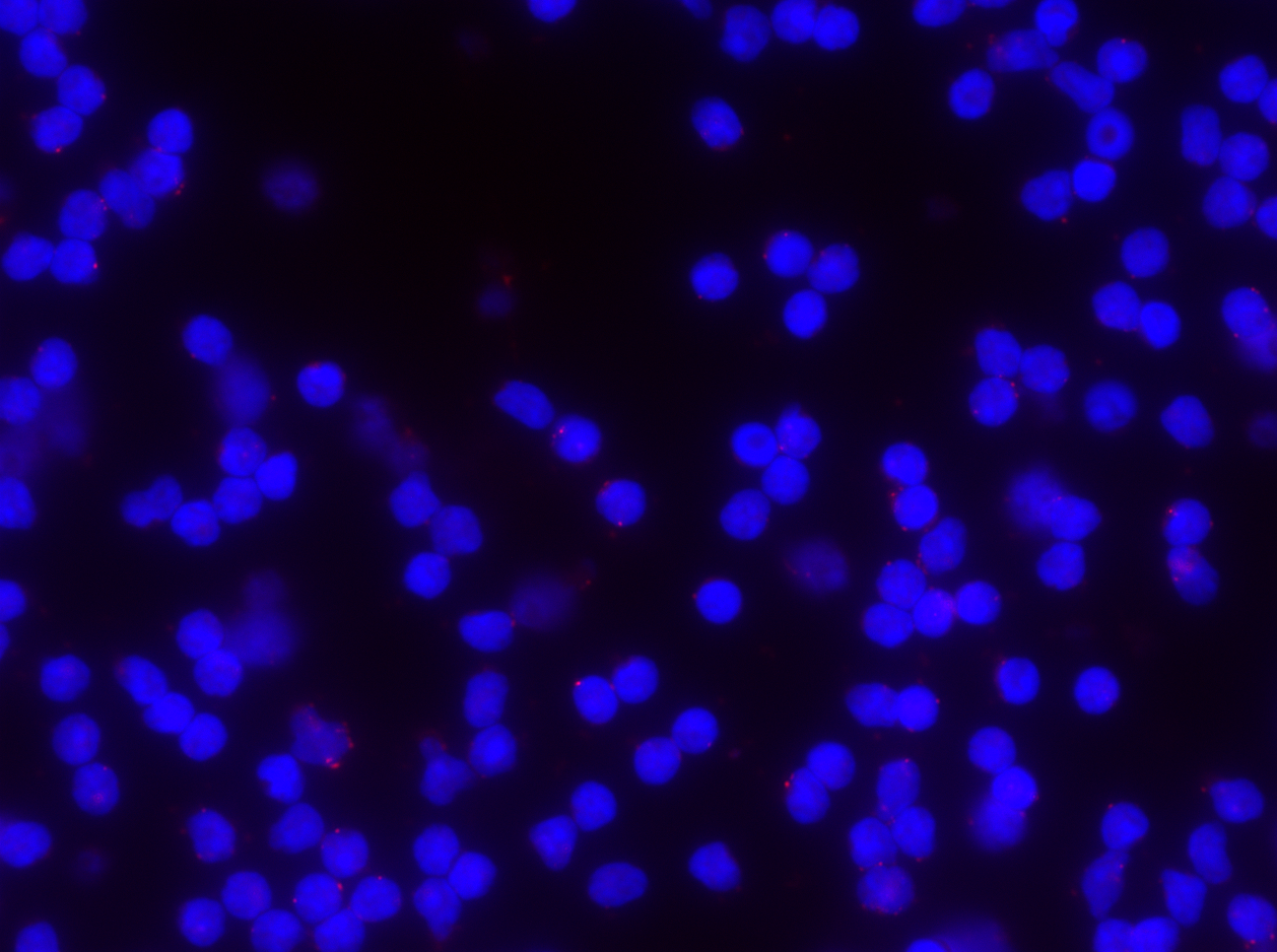

Supplement: Supplementary file 11 — Source data Fig. 7 [file 44318_2026_781_MOESM11_ESM.zip › Figure 7/D/Fig 7D Tax+RTX.png]
