# Supplementary material for: CD20 tails interact with the 14-3-3/GEF-H1 complex and microtubule network upon PKCδ phosphorylation
Source: EMBO J. 2026 Apr 17;45(11):3859–79. doi: 10.1038/s44318-026-00781-5 (PMC13226681; doi:10.1038/s44318-026-00781-5)
Supplement: Supplementary file 7 — Source data Fig. 3 [file 44318_2026_781_MOESM7_ESM.zip › Figure 3/A/Fig 3 A CD20 human Uniprot.docx]

CD20 human Uniprot

<https://www.uniprot.org/uniprotkb/P11836/entry>

P11836 · CD20_HUMAN
