# Supplementary material for: CD20 tails interact with the 14-3-3/GEF-H1 complex and microtubule network upon PKCδ phosphorylation
Source: EMBO J. 2026 Apr 17;45(11):3859–79. doi: 10.1038/s44318-026-00781-5 (PMC13226681; doi:10.1038/s44318-026-00781-5)
Supplement: Supplementary file 7 — Source data Fig. 3 [file 44318_2026_781_MOESM7_ESM.zip › Figure 3/B/Protter- interactive protein feature visualization and integration with experimental proteomic data.pdf]

BIOINFORMATICS APPLICATIONS NOTE
Vol. 30 no. 6 2014, pages 884–886
doi:10.1093/bioinformatics/btt607

Sequence analysis
Advance Access publication October 24, 2013

Protter: interactive protein feature visualization and integration with experimental proteomic data

Ulrich Omasits[1,2], Christian H. Ahrens[2], Sebastian Müller[1] and Bernd Wollscheid[1,*]
[1]Department of Biology, Institute of Molecular Systems Biology, ETH Zürich, 8093 Zürich and [2]Institute of Molecular Life Sciences, University of Zürich, 8057 Zürich, Switzerland
Associate Editor: Mario Albrecht

ABSTRACT

Summary: The ability to integrate and visualize experimental proteomic evidence in the context of rich protein feature annotations represents an unmet need of the proteomics community. Here we present Protter, a web-based tool that supports interactive protein data analysis and hypothesis generation by visualizing both annotated sequence features and experimental proteomic data in the context of protein topology. Protter supports numerous proteomic file formats and automatically integrates a variety of reference protein annotation sources, which can be readily extended via modular plug-ins. A built-in export function produces publication-quality customized protein illustrations, also for large datasets. Visualizations of surfaceome datasets show the specific utility of Protter for the integrated visual analysis of membrane proteins and peptide selection for targeted proteomics.

Availability and implementation: The Protter web application is available at http://wlab.ethz.ch/protter. Source code and installation instructions are available at http://ulo.github.io/Protter/.

Contact: wbernd@ethz.ch

Supplementary Information: Supplementary data are available at *Bioinformatics* online.

Received on February 1, 2013; revised on October 5, 2013; accepted on October 21, 2013

1 INTRODUCTION

Visualization of features within biological sequences is essential for integrated analysis and interpretation of experimental sequencing data. Software solutions to visualize nucleic acid sequences such as genomic DNA in the context of rich feature annotations (e.g. genes, promoters) and experimental evidence (e.g. RNA-Seq reads) have become indispensable research tools for interactive data analysis and hypothesis generation. Prominent examples include the UCSC genome browser (Meyer *et al*., 2012) and IGV (Robinson *et al*., 2011). In contrast, only few tools support the visualization of protein features such as post-translational modifications (PTMs) or protein domains within the context of a protein's topology and—like the examples above—down to single residue resolution. These include RbDe (Skrabanek *et al*., 2003), TMRPres2D (Spyropoulos *et al*., 2004) and TOPO2 (http://www.sacs.ucsf.edu/TOPO2). Importantly, none of these tools integrates experimental proteomic data in an automated fashion (see Supplementary Table S1 and Fig. S1 for a detailed comparison).

To exploit the unique insights into protein structure and function that experimental proteomic data provide, tools are needed that would allow for their integrated visualization, interactive analysis and hypothesis generation (Gehlenborg *et al*., 2010). For membrane proteins, which carry out many essential cellular functions and represent more than half of all current drug targets (von Heijne, 2007), taking into account the topology is particularly important.

Protter is an interactive and customizable web-based application that enables the integration and visualization of both annotated and predicted protein sequence features together with experimental proteomic evidence for peptides and PTMs, onto the transmembrane topology of a protein. It allows users to choose from numerous annotation sources, integrate own proteomics data files, select best-suited peptides for targeted quantitative proteomics applications and export publication-quality illustrations.

2 METHODS

Protter was designed as a web-based software using a client-server architecture (Fig. 1A). The Protter server application is written in Java and communicates with clients using HTTP: parameters (Supplementary Table S3) are specified in a GET request and the protein visualization is returned. Clients can be desktop or web applications (e.g. the Protter web application), scripts (e.g. for advanced batch plot generation) or any other tool capable of loading images from the web using HTTP. Thus, Protter could also be integrated with popular web services like STRING (Szklarczyk *et al*., 2011), UniProt (Magrane *et al*., 2011) or neXtProt (Lane *et al*., 2011). For each request, the Protter server application gathers (i) protein topology information from UniProt or Phobius (Käll *et al*., 2007), (ii) detailed protein feature annotation from UniProt, (iii) proteolytic peptides from PeptideCutter and (iv) experimental proteomic data from a user's file or from repositories like PeptideAtlas (Deutsch, 2010). The list of available annotation sources (Supplementary Table S2) can be extended using Java plug-ins. Internally, Protter uses the LaTeX typesetting system with the TeXtopo (Beitz, 2000) macro package to generate the topological layout of the protein plot, which is processed and returned to the client. A multi-level cache strategy is used to minimize response time.

The Protter web application (Fig. 1B) offers an intuitive way to specify all parameters for plotting using a web browser. It communicates to the Protter server via AJAX and presents an interactive inline display of the resulting protein plot. Proteomic result files are loaded and analyzed on the client side, allowing for responsive browsing through proteins and peptides.

*To whom correspondence should be addressed.

© The Author 2013. Published by Oxford University Press. All rights reserved. For Permissions, please e-mail: journals.permissions@oup.com

Downloaded from https://academic.oup.com/bioinformatics/article/30/6/884/285666 by Albert-Ludwigs-Universitaet Freiburg user on 28 October 2025

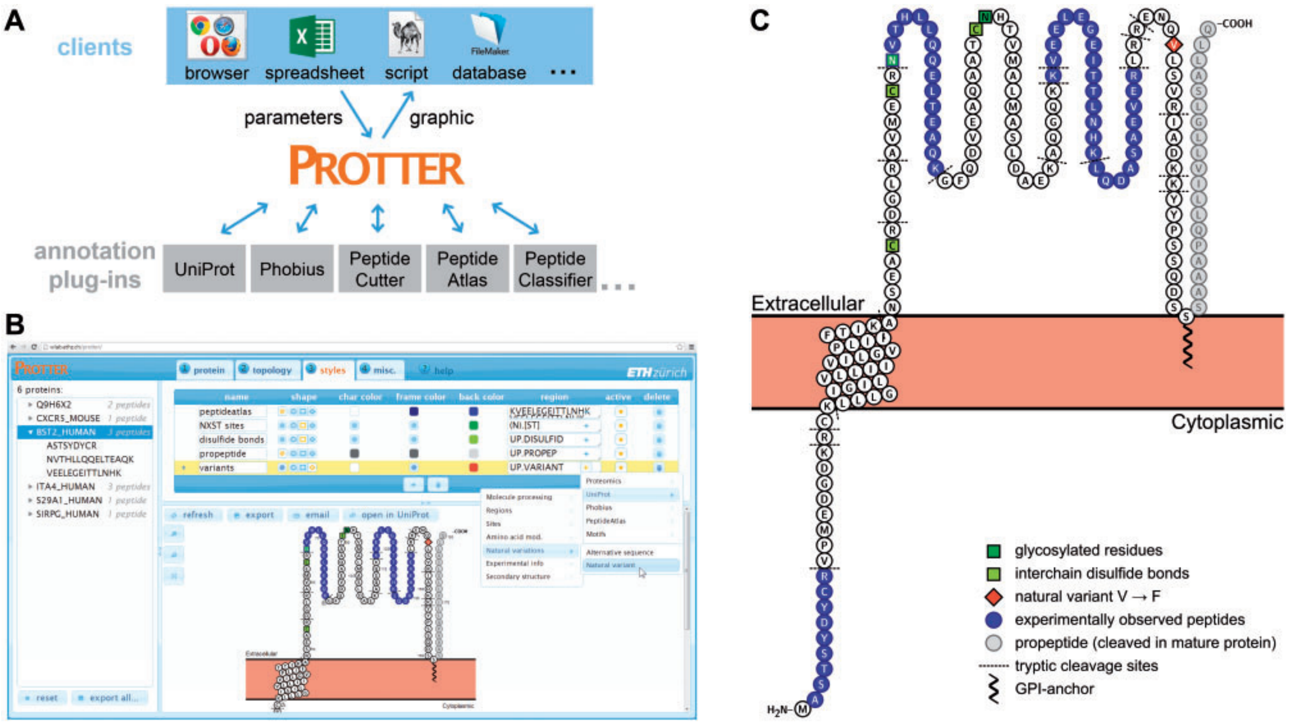

Fig. 1. (**A**) Client-server architecture of Protter: the server acts as a RESTful web service and gathers protein features from various annotation sources. (**B**) Screenshot of the Protter web application, which supports interactive annotation of protein sequence features. (**C**) Protter illustration of a human protein (CD317) with annotation of various UniProt features, experimentally observed peptides from PeptideAtlas and tryptic cleavage sites from PeptideCutter

3 RESULTS

The Protter web application enables users to generate custom protein feature visualizations right in their browser by following four easy steps: (i) A UniProt identifier or a protein sequence is specified. (ii) The protein's transmembrane topology is defined. (iii) Various protein features (e.g. PTMs, secondary structure, sequence motifs, custom regions of interest, etc.; see Supplementary Table S2 for supported annotation sources) are highlighted. (iv) Additional options for the visualization are set. Selected features are highlighted in the residue-based protein diagram by varying four parameters: the color of the amino acid letter, the shape of the amino acid symbol, the symbol's frame color and the symbol's background color. As Protter chooses appropriate default settings automatically, all but the first step are optional. The interactive visualization of the chosen protein is displayed freely scalable in the browser (Fig. 1C). An online help guides the user through all steps and provides documentation on underlying annotation sources and technologies used.

A distinctive key feature of Protter is the ability to overlay experimental proteomic data either from individual experiments [result files of PeptideProphet (Keller *et al.*, 2002), ProteinProphet (Nesvizhskii *et al.*, 2003), Mascot (Perkins *et al.*, 1999), MaxQuant (Cox and Mann, 2008) and Skyline (MacLean *et al.*, 2010) are supported] or large data collections like PeptideAtlas. Identified proteins, peptides and PTMs can be inspected individually in the browser and a combined visualization of all proteins can be downloaded as one PDF document or as ZIP archive of separate files.

Protter supports several common usage scenarios, such as the visual inspection of a protein's transmembrane topology, the integrated assessment of both predicted and experimental peptide and PTM evidence, the generation of publication-quality figures and a wide range of specific applications in proteomics research:

- Protter supports targeted proteomic approaches like selected reaction monitoring (SRM) (Lange *et al.*, 2008), which are based on selection of peptides that unambiguously identify and reproducibly quantify a specific protein of interest. Through integrated visualization of protease cleavage sites, peptide evidence classes (Qeli and Ahrens, 2010), previously experimentally identified peptides [e.g. from PeptideAtlas, UniPep (Zhang *et al.*, 2006), N-glycoprotein SRMAtlas (Hüttenhain *et al.*, 2013)], transmembrane regions and any known PTM or sequence variant, suitable peptide candidates can be visualized and selected intuitively before the actual and after the proteomic experiment (see Supplementary Fig. S2). To facilitate this process, Protter is supported via a plug-in from the leading targeted proteomics software environment Skyline (see Supplementary Fig. S3).
- Proteomic evidence from surfaceome research (Wollscheid *et al.*, 2009; Bock *et al.*, 2012) generates glycopeptide-derived topological constraints (Tsirigos *et al.*, 2012), where visualization using Protter aids in the better prediction and validation of the actual transmembrane topology of cell surface proteins (see Supplementary Fig. S4).

Downloaded from https://academic.oup.com/bioinformatics/article/30/6/884/285666 by Albert-Ludwigs-Universitaet Freiburg user on 28 October 2025

- Ligand-based receptor-capture (LRC) technology (Frei *et al.*, 2012) allows MS-based identification of receptor proteins to orphan ligands. Potential receptors need to have cell surface–exposed N-glycosylation sites within MS-compatible tryptic peptides to be identifiable. Using a Protter visualization, one can judge at a glance whether a potential receptor will be amenable to LRC technology.
- Phosphorylation site identifications from phosphoproteomic screens can be visualized in the context of annotated phosphorylation sites as well as known protein kinase sequence motifs, giving a direct hint on a protein's potential role in the cell signaling network.
- Experimentally identified peptides from large datasets can be easily visualized via the web interface, as demonstrated in a combined PDF (Supplementary Fig. S5) of 267 plots visualizing proteins with predicted transmembrane domains from the first complete membrane proteome described (Omasits *et al.*, 2013). This big dataset was visualized in only a couple of minutes—less than it takes to create a single protein plot using any of the other tools.

All of Protter's functionalities described above can be accessed through the user-friendly web interface, without writing a single line of code—another feature unique to Protter. However, for more refined automation purposes, the Protter server API is simple to use from any scripting language, as exemplified in Supplementary Listing S1 and Figure S6 for the visualization of sequence conservation, or Supplementary Listing S2 for the usage of TMHMM instead of Phobius for transmembrane topology prediction.

In summary, the open source application Protter supports the customizable visualization of protein sequence features in the context of protein topology and experimental proteomic evidence in publication quality, for single proteins as well as for large-scale proteomic datasets. This, in turn, enables direct visual insights pre- and post-publication of discovery-driven and targeted proteomic datasets.

ACKNOWLEDGEMENTS

The authors thank Ruedi Aebersold, Christian von Mering, Amos Bairoch, Daniel Broudy, Brendan MacLean, Alexey Nesvizhskii, Rebekah Gundry and Eric Deutsch for help and feedback on the software and manuscript.

Funding: Swiss National Science Foundation (grants 31003A_135805 to B.W. and 31003A_130723 to C.H.A.); and SystemsX.ch (grant InfectX to B.W. and grant IPP 2011/121 to C.H.A.).

Conflict of Interest: none declared.

REFERENCES

Beitz,E. (2000) T(E)Xtopo: shaded membrane protein topology plots in LAT(E)X2epsilon. *Bioinformatics*, **16**, 1050–1051.

Bock,T. *et al.* (2012) CD proteome and beyond - Technologies for targeting the immune cell surfaceome. *Front. Biosci.*, **17**, 1599–1612.

Cox,J. and Mann,M. (2008) MaxQuant enables high peptide identification rates, individualized p.p.b.-range mass accuracies and proteome-wide protein quantification. *Nat. Biotechnol.*, **26**, 1367–1372.

Deutsch,E.W. *et al.* (2010) The PeptideAtlas Project. *Methods Mol. Biol.*, **604**, 285–296.

Frei,A.P. *et al.* (2012) Direct identification of ligand-receptor interactions on living cells and tissues. *Nat. Biotechnol.*, **30**, 997–1001.

Gehlenborg,N. *et al.* (2010) Visualization of omics data for systems biology. *Nat. Methods*, **7**, S56–S68.

von Heijne,G. (2007) The membrane protein universe: what's out there and why bother? *J. Intern. Med.*, **6**, 543–557.

Hüttenhain,R. *et al.* (2013) N-glycoprotein SRMAtlas: a resource of mass spectrometric assays for N-glycosites enabling consistent and multiplexed protein quantification for clinical applications. *Mol. Cell. Proteomics*, **12**, 1005–1016.

Käll,L. *et al.* (2007) Advantages of combined transmembrane topology and signal peptide prediction–the Phobius web server. *Nucleic Acids Res.*, **35**, W429–W432.

Keller,A. *et al.* (2002) Empirical statistical model to estimate the accuracy of peptide identifications made by MS/MS and database search. *Anal. Chem.*, **74**, 5383–5392.

Lane,L. *et al.* (2011) neXtProt: a knowledge platform for human proteins. *Nucleic Acids Res.*, **40**, D76–D83.

Lange,L. *et al.* (2008) Selected reaction monitoring for quantitative proteomics: a tutorial. *Mol. Syst. Biol.*, **4**, 222.

MacLean,B. *et al.* (2010) Skyline: an open source document editor for creating and analyzing targeted proteomics experiments. *Bioinformatics*, **26**, 966–968.

Magrane,M. *et al.* (2011) UniProt Knowledgebase: a hub of integrated protein data. *Database*, **2011**, bar009.

Meyer,L.R. *et al.* (2012) The UCSC Genome Browser database: extensions and updates 2013. *Nucleic Acids Res.*, **41**, D64–D69.

Nesvizhskii,A.I. *et al.* (2003) A statistical model for identifying proteins by tandem mass spectrometry. *Anal. Chem.*, **75**, 4646–4658.

Omasits,U. *et al.* (2013) Directed shotgun proteomics guided by saturated RNA-seq identifies a complete expressed prokaryotic proteome. *Genome Res.*, **23**, 1916–1927.

Perkins,D.N. *et al.* (1999) Probability-based protein identification by searching sequence databases using mass spectrometry data. *Electrophoresis*, **20**, 3551–3567.

Qeli,E. and Ahrens,C.H. (2010) PeptideClassifier for protein inference and targeted quantitative proteomics. *Nat. Biotechnol.*, **28**, 647–650.

Robinson,J.T. *et al.* (2011) Integrative genomics viewer. *Nat. Biotechnol.*, **29**, 24–26.

Skrabanek,L. *et al.* (2003) Building protein diagrams on the web with the residue-based diagram editor RbDe. *Nucleic Acids Res.*, **31**, 3856–3858.

Spyropoulos,I.C. *et al.* (2004) TMRPres2D: high quality visual representation of transmembrane protein models. *Bioinformatics*, **20**, 3258–3260.

Szklarczyk,D. *et al.* (2011) The STRING database in 2011: functional interaction networks of proteins, globally integrated and scored. *Nucleic Acids Res.*, **39**, D561–D568.

Tsirigos,K.D. *et al.* (2012) A guideline to proteome-wide α-helical membrane protein topology predictions. *Proteomics*, **12**, 2282–2294.

Wollscheid,B. *et al.* (2009) Mass-spectrometric identification and relative quantification of N-linked cell surface glycoproteins. *Nat. Biotechnol.*, **27**, 378–386.

Zhang,H. *et al.* (2006) UniPep–a database for human N-linked glycosites: a resource for biomarker discovery. *Genome Biol.*, **7**, R73.

Downloaded from https://academic.oup.com/bioinformatics/article/30/6/884/285666 by Albert-Ludwigs-Universitaet Freiburg user on 28 October 2025
