# Supplementary material for: CD20 tails interact with the 14-3-3/GEF-H1 complex and microtubule network upon PKCδ phosphorylation
Source: EMBO J. 2026 Apr 17;45(11):3859–79. doi: 10.1038/s44318-026-00781-5 (PMC13226681; doi:10.1038/s44318-026-00781-5)
Supplement: Supplementary file 7 — Source data Fig. 3 [file 44318_2026_781_MOESM7_ESM.zip › Figure 3/B/Fig 3 B protter.docx]

<http://wlab.ethz.ch/protter/start/>

P11836 · CD20_HUMAN

http://wlab.ethz.ch/protter/#up=P11836&tm=auto&mc=lightsalmon&lc=blue&tml=numcount&numbers&legend&n:signal%20peptide,fc:red,bc:red=UP.SIGNAL&n:disulfide%20bonds,s:box,fc:greenyellow,bc:greenyellow=UP.DISULFID&n:variants,s:diamond,fc:orange,bc:orange=UP.VARIANT&n:PTMs,s:box,fc:forestgreen,bc:forestgreen=UP.CARBOHYD,UP.MOD_RES&format=svg
