# Supplementary material for: CD20 tails interact with the 14-3-3/GEF-H1 complex and microtubule network upon PKCδ phosphorylation
Source: EMBO J. 2026 Apr 17;45(11):3859–79. doi: 10.1038/s44318-026-00781-5 (PMC13226681; doi:10.1038/s44318-026-00781-5)
Supplement: Supplementary file 8 — Source data Fig. 4 [file 44318_2026_781_MOESM8_ESM.zip › Figure 4/A/p21-activated Kinase 1 Phosphorylates and Regulates 14-3-3 Binding to GEF-H1, a Microtubule-localized Rho Exchange Factor.pdf]

THE JOURNAL OF BIOLOGICAL CHEMISTRY
Vol. 279, No. 18, Issue of April 30, pp. 18392–18400, 2004
Printed in U.S.A.
© 2004 by The American Society for Biochemistry and Molecular Biology, Inc.

p21-activated Kinase 1 Phosphorylates and Regulates 14-3-3 Binding to GEF-H1, a Microtubule-localized Rho Exchange Factor*Ⓢ

Received for publication, January 6, 2004, and in revised form, February 7, 2004
Published, JBC Papers in Press, February 17, 2004, DOI 10.1074/jbc.M400084200

Frank T. Zenke‡§, Mira Krendel‡¶, Celine DerMardirossian, Charles C. King‖, Benjamin P. Bohl, and Gary M. Bokoch**

From the Departments of Immunology and Cell Biology, The Scripps Research Institute, La Jolla, California 92037

GEF-H1 is a guanine nucleotide exchange factor for Rho whose activity is regulated through a cycle of microtubule binding and release. Here we identify a region in the carboxyl terminus of GEF-H1 that is important for suppression of its guanine nucleotide exchange activity by microtubules. This portion of the protein includes a coiled-coil motif, a proline-rich motif that may interact with Src homology 3 domain-containing proteins, and a potential binding site for 14-3-3 proteins. We identify GEF-H1 as a binding target and substrate for p21-activated kinase 1 (PAK1), an effector of Rac and Cdc42 GTPases, using an affinity-based screen and localize a PAK1 phosphorylation site to the inhibitory carboxyl-terminal region of GEF-H1. We show that phosphorylation of GEF-H1 at Ser^{885} by PAK1 induces 14-3-3 binding to the exchange factor and relocation of 14-3-3 to microtubules. Phosphorylation of GEF-H1 by PAK may be involved in regulation of GEF-H1 activity and may serve to coordinate Rho-, Rac-, and Cdc42-mediated signaling pathways.

Rho GTPases play an important role in regulation of many cellular processes, including cytoskeletal dynamics and cell motility; gene transcription; and cell growth, differentiation, and death. Their activity is controlled by regulatory proteins such as guanine nucleotide exchange factors (GEFs)[1] and GTPase-activating proteins. GEFs catalyze the exchange of GTP for GDP, thereby switching the GTPase to an active conformation. GTPase-activating proteins enhance the intrinsic GTPase activity of Rho GTPases, leading to GTP hydrolysis and inactivation of the GTPase.

Cell migration is critical to development, wound healing, and the immune response. It is increasingly evident that coordinated action of the actin and microtubular cytoskeletons is required for persistent and directed cell motility. Protrusion of the leading edge depends upon actin polymerization into a dense cross-linked meshwork of actin fibers, while retraction of the rear involves contractile actin/myosin bundles in the cell body (1, 2). The dynamics of these processes are controlled by the action of Rho GTPases (3). Rac and Cdc42 are required for lamellipodial actin polymerization and filopodia formation, respectively, while RhoA regulates actin bundling into contractile stress fibers and modulates myosin-dependent tail retraction (4). Similarly, an intact microtubule system is required to maintain polarity and directed movement of tissue cells (5, 6). Microtubule growth at the leading edge promotes Rac activation (7), which in turn acts through p21-activated kinase 1 (PAK1) and Op18 to enhance microtubule growth in a positive feedback cycle (8). Physiological or drug-induced microtubule depolymerization in the cell body and tail leads to activation of RhoA (9, 10). At least part of this microtubule-regulated RhoA activation is mediated through the Rho exchange factor GEF-H1.

GEF-H1 was originally identified as a microtubule-associated GEF with activity toward Rho (11). We have characterized cellular regulation of GEF-H1 and determined that the guanine nucleotide exchange activity of full-length GEF-H1 is inhibited by interaction with microtubules (12). Release from microtubules results in increased GEF activity toward RhoA, and a dominant inhibitory version of GEF-H1 blocks nocodazole-induced RhoA activation. The amino- and carboxyl-terminal regions of GEF-H1 are involved in co-localization of GEF-H1 with microtubules (12). Deletion and/or mutation of these regions results in the loss of microtubule co-localization and up-regulation of GEF-H1 Rho exchange activity. To further dissect the role of microtubules in regulation of the activity of GEF-H1, we produced fusion proteins consisting of the microtubule-binding region of MAP2c (13) and GEF-H1 mutants deficient in microtubule binding. The use of the MAP2c fragment enabled us to restore microtubule targeting of GEF-H1 mutants and to investigate the effect of such targeting on GEF-H1 activity. This approach led to identification of the carboxyl terminus of GEF-H1 as a region crucial for regulation of the activity of the microtubule-bound protein.

PAKs are serine/threonine kinases whose activity is regulated by the binding of activated Rac or Cdc42 GTPases (14). PAKs have been implicated in the regulation of multiple cellular activities, including dynamics of the actin and microtubular cytoskeletons (8, 15). In an independent biochemical screen for novel substrates of PAK1, we identified GEF-H1 as a phosphorylation target of PAK1. A PAK1 phosphorylation site in GEF-H1 was mapped to the carboxyl-terminal portion of the

* The costs of publication of this article were defrayed in part by the payment of page charges. This article must therefore be hereby marked "*advertisement*" in accordance with 18 U.S.C. Section 1734 solely to indicate this fact.

Ⓢ The on-line version of this article (available at http://www.jbc.org) contains Supplemental Fig. 1.

‡ Both authors contributed equally to this work.

§ Present address: Merck KgaA, Oncology Research Darmstadt, Frankfurter Strasse 250, 64293 Darmstadt, Germany.

¶ Present address: Dept. of Molecular, Cellular, and Developmental Biology, KBT-342, Yale University, 266 Whitney Ave., New Haven, CT 06520.

‖ Present address: Dept. of Pharmacology, University of California, 9500 Gilman Dr., San Diego, CA 92093.

** To whom correspondence should be addressed: Depts. of Immunology and Cell Biology, The Scripps Research Inst., IMM14, 10550 North Torrey Pines Rd., La Jolla, CA 92037. Tel.: 858-874-8217; Fax: 858-784-8218; E-mail: bokoch@scripps.edu.

[1] The abbreviations used are: GEF, guanine nucleotide exchange factor; PAK, p21-activated kinase; SH3, Src homology 3; HA, hemagglutinin; EGFP, enhanced green fluorescent protein; GST, glutathione *S*-transferase; MALDI, matrix-assisted laser desorption ionization; TOF, time-of-flight; GTPγS, guanosine 5′-3-*O*-(thio)triphosphate; SRE, serum response element.

This paper is available on line at http://www.jbc.org

This is an Open Access article under the CC BY license.

protein that is involved in regulation of its activity when bound to microtubules. The identified PAK phosphorylation site is embedded in a predicted 14-3-3 binding motif, and we demonstrate that phosphorylation of GEF-H1 at Ser^{885} by PAK1 regulates the docking of 14-3-3 to GEF-H1 and its recruitment to microtubules. These observations suggest that PAK and 14-3-3 are involved in regulation of GEF-H1 activity and that phosphorylation of GEF-H1 by PAK may act to coordinate Rac/Cdc42- and Rho-dependent signaling pathways.

EXPERIMENTAL PROCEDURES

Plasmids—GEF-H1 and KIAA-0651 constructs in the mammalian expression vectors pCMV5-HA_3 and pCMV5-EGFP have been described previously (12). These vectors are derivatives of pCMV5 that contain a triple hemagglutinin (HA) epitope or EGFP inserted between EcoRI and KpnI sites for amino-terminal fusion. GEF-H1 or 0651 subregions (indicated by the first and last amino acid position) were inserted into pCMV5-HA_3 or -EGFP between the KpnI and ClaI sites or into the bacterial expression vector pGEX-KG between EcoRI and HindIII. Rat MAP2c fragment (amino acids 231–467), generously provided by Dr. Shelley Halpain, was subcloned into pCMV5-EGFP vector either using the KpnI site upstream of the GEF-H1 insert or using ClaI/HindIII sites downstream of GEF-H1. An inactive mutant of GST-PAK1 (D389A) was constructed using site-directed mutagenesis. This mutant was inactive in *in vitro* kinase assay and did not bind GEF-H1 (data not shown). Plasmids encoding HA-tagged 14-3-3 ϵ and ζ were a generous gift of Dr. Michael Yaffe.

Cell Culture and Transfections—Jurkat T lymphoblast cells were grown in RPMI 1640 medium containing 10% fetal bovine serum, 10 mM glutamine, 10 mM HEPES, and antibiotics (penicillin and streptomycin). COS-1 and HeLa cells were grown in Dulbecco's modified Eagle's medium with the same additives as for Jurkat cells. COS-1 and HeLa cells were transfected using LipofectAMINE (Invitrogen) according to the manufacturer's protocol. 5 μg of expression plasmid and 15 μl of LipofectAMINE were generally used per 10-cm dish. For immunofluorescence labeling, HeLa cells were plated onto coverslips in 35-mm dishes and transfected using 5 μl of LipofectAMINE and 0.75 μg of each plasmid. Cell lysates were prepared 48 h post-transfection by lysis in 25 mM Tris/HCl, pH 7.5, 150 mM NaCl, 5 mM $MgCl_2$, 1 mM EDTA, 0.1 mM EGTA, 1 mM dithiothreitol, 1% Nonidet P-40, and 10% glycerol with protease inhibitors for 5 min on ice followed by centrifugation for 5 min at 10,000 × *g*. The supernatants (=lysate) were used for immunoprecipitations, binding reactions, or Western blot analysis.

Immunofluorescence Staining and Image Analysis—Immunofluorescence staining and cell imaging were performed as described previously (12). The degree of cytoplasmic/cytoskeletal localization of EGFP-tagged GEF-H1 constructs was measured using quantitative image analysis as described by Ozer and Halpain (16). Briefly standard deviation of fluorescence intensity was measured for all pixels within each cell expressing EGFP-tagged protein. Pixels within cells expressing microtubule-bound forms of GEF-H1 exhibited high variability in fluorescence intensity, while non-microtubule-bound constructs were more evenly distributed in the cytoplasm and exhibited lower variability in fluorescence intensity. The standard deviation value for each cell was divided by the average fluorescence intensity within the cell to eliminate differences due to the amount of protein expressed. Normalized fluorescence intensity variation values for each construct were averaged and compared using a *t* test. At least 40 cells were scored for each construct.

Purification of PAK Substrates—5–20 μg of GST-PAK1-(233–544) attached to glutathione-Sepharose beads were incubated with 1–5 mg of total Jurkat cell lysate for 30 min–1 h in a volume up to 1.5 ml and subsequently washed four times with lysis buffer. The binding reaction was separated on 10% SDS-polyacrylamide gels and stained with Coomassie Brilliant Blue. The 110-kDa PAK1 target protein was excised with a razor blade and washed three times with water and two times with 25 mM NH_4HCO_3. The gel pieces were gently crushed with a pestle, and 100 μl of 25 mM NH_4HCO_3 and 200–250 ng of trypsin per sample were added, and then the samples were incubated overnight at 37 °C. The supernatant was discarded, and the gel pieces were vortexed in 50 μl of 50% acetonitrile for 2 min and centrifuged briefly at full speed, and the supernatant was transferred into a new vial. The extraction procedure was repeated once. Acetonitrile was removed under bubbling nitrogen, and the cleaved peptides were dried under vacuum. Samples were resuspended in 10 μl of 5% acetonitrile, 0.1% trifluoroacetic acid and bound to ZipTips (Millipore, C_{18} resin material). The resin was washed with 0.1% trifluoroacetic acid and eluted in 2 μl of 50% acetonitrile. 1 μl of the eluted peptides was mixed with 1 μl of saturated 2,5-dihydroxybenzoic acid (Sigma) in 50% acetonitrile, 0.1% trifluoroacetic acid, and 1 μl of the peptide/2,5-dihydroxybenzoic acid mixture was spotted onto a MALDI grid and co-crystallized by drying (dried droplet procedure). Bovine serum albumin as a control protein was purified and processed accordingly. A Dynamo MALDI-TOF mass spectrometer was used for peptide mass determination. For mass calibration we used control peptides (angiotensin I, 1296.685 Da; renin tetradecapeptide, 1759.853 Da; insulin, 5434.59 Da). The peptide masses determined were used to search a protein data base using the ProFound software (17).

For *in vitro* phosphorylation by PAK, equivalent amounts of the binding reactions were incubated for 30 min at 30 °C in the presence of 25 μM ATP and 10 μCi of radiolabeled ATP/reaction (specific activity, ~10,000 dpm/pmol). $[^{32}P]$GEF-H1 was quantified using a PhosphorImager (Amersham Biosciences).

Phosphopeptide Mapping by Two-dimensional Chromatography—Phosphopeptide mapping was performed as described previously (18) using recombinant GST-GEF-H1 (the full length or amino acids 571–985) phosphorylated with GST-PAK1-(233–544) *in vitro* or with full-length $PAK1^{T423E,L107F}$ *in vivo*. For the latter, HeLa cells were metabolically labeled with 0.4 mCi of $[\gamma\text{-}^{32}P]$orthophosphoric acid for 6 h at 37 °C and washed twice, and GEF-H1 was immunoprecipitated from cell lysates with anti-EGFP antibody. The $\gamma\text{-}^{32}P$-phosphorylated GEF-H1 was detected by autoradiography, excised from nitrocellulose, and digested with 1.5 μg of trypsin for 14 h at 37 °C. The digest (8000 cpm) was resolved in two dimensions on 100-μm × 20-cm × 20-cm thin layer cellulose plates (EM Science, Gibbstown, NJ) by electrophoresis followed by ascending chromatography. Electrophoresis was performed at pH 1.9 in 2.2% formic acid, 8% acetic acid for 40 min at 1300 V in a Multiphor II horizontal electrophoresis unit (Amersham Biosciences) with water cooling, and ascending chromatography was carried out in 62.5% isobutyric acid, 1.9% *n*-butyl alcohol, 4.8% pyridine, and 2.9% glacial acetic acid. The plates were exposed to x-ray film overnight at −80 °C.

Phosphoamino Acid Analysis—GST-GEF-H1-(571–985) was phosphorylated as described above, the phosphorylated protein was localized by autoradiography, and the band was excised from the nitrocellulose. The sample was digested with 1.5 μg of trypsin and directly subjected to hydrolysis with 6 N HCl at 110 °C for 1 h in a sealed tube (18). The hydrolyzed sample was dried using a Speed-Vac (Savant) concentrator, resuspended in 10 μl of 2.2% formic acid, 8% acetic acid (pH 1.9), spotted onto a thin layer cellulose plate, and subjected to electrophoresis at 1500 V for 20 min. Phosphoserine, phosphothreonine, and phosphotyrosine (1 mg/ml) were used as markers. The second electrophoretic separation was performed in 5% acetic acid, 0.5% pyridine (pH 3.5) at 1500 V for 20 min. The plate was then dried and sprayed with ninhydrin to visualize the phosphoamino acid standards. ^{32}P-Labeled proteins on the dried plates were detected by autoradiography.

In Vitro and in Vivo Measurements of GEF-H1 Activity—For *in vitro* measurement of nucleotide exchange activity of GEF-H1 toward Rho we utilized a method by Downward (19) described for immunoprecipitated proteins with minor modifications as in Ref. 12. Immunoprecipitated exchange factor or purified binding reactions were equilibrated in exchange buffer and used to catalyze $[^{35}S]GTP\gamma S$ binding to RhoA. *In vivo* activity of GEF-H1 was measured using an SRE-luciferase reporter gene assay as described previously (12). Briefly GEF-H1 constructs were transfected into COS-1 cells together with SRE-luciferase and β-galactosidase reporter plasmids. Expression of luciferase induced by activated Rho was measured using a luminometer and normalized by the amount of β-galactosidase expressed.

Binding of 14-3-3 to GEF-H1—GST-GEF-H1-(571–985) was phosphorylated by recombinant GST-PAK1 *in vitro* for 30 min in the presence of 0.5 mM ATP. Samples were washed, incubated for 1 h at 4 °C with bovine brain lysate, and further washed to remove nonspecifically bound proteins. For co-immunoprecipitation of 14-3-3 with GEF-H1, COS-1 cells were transfected with the HA-tagged GEF-H1 and Myc-tagged PAK1, and GEF-H1 was immunoprecipitated from cell lysates. 14-3-3 bound to GST-GEF-H1 or HA-GEF-H1 was detected by Western blotting using a polyclonal anti-14-3-3 antiserum (Santa Cruz Biotechnology, Santa Cruz, CA).

RESULTS

Characterization of the Region of GEF-H1 Responsible for Microtubule-dependent Regulation of Its Rho Guanine Nucleotide Exchange Activity—GEF-H1 contains a central tandem dbl

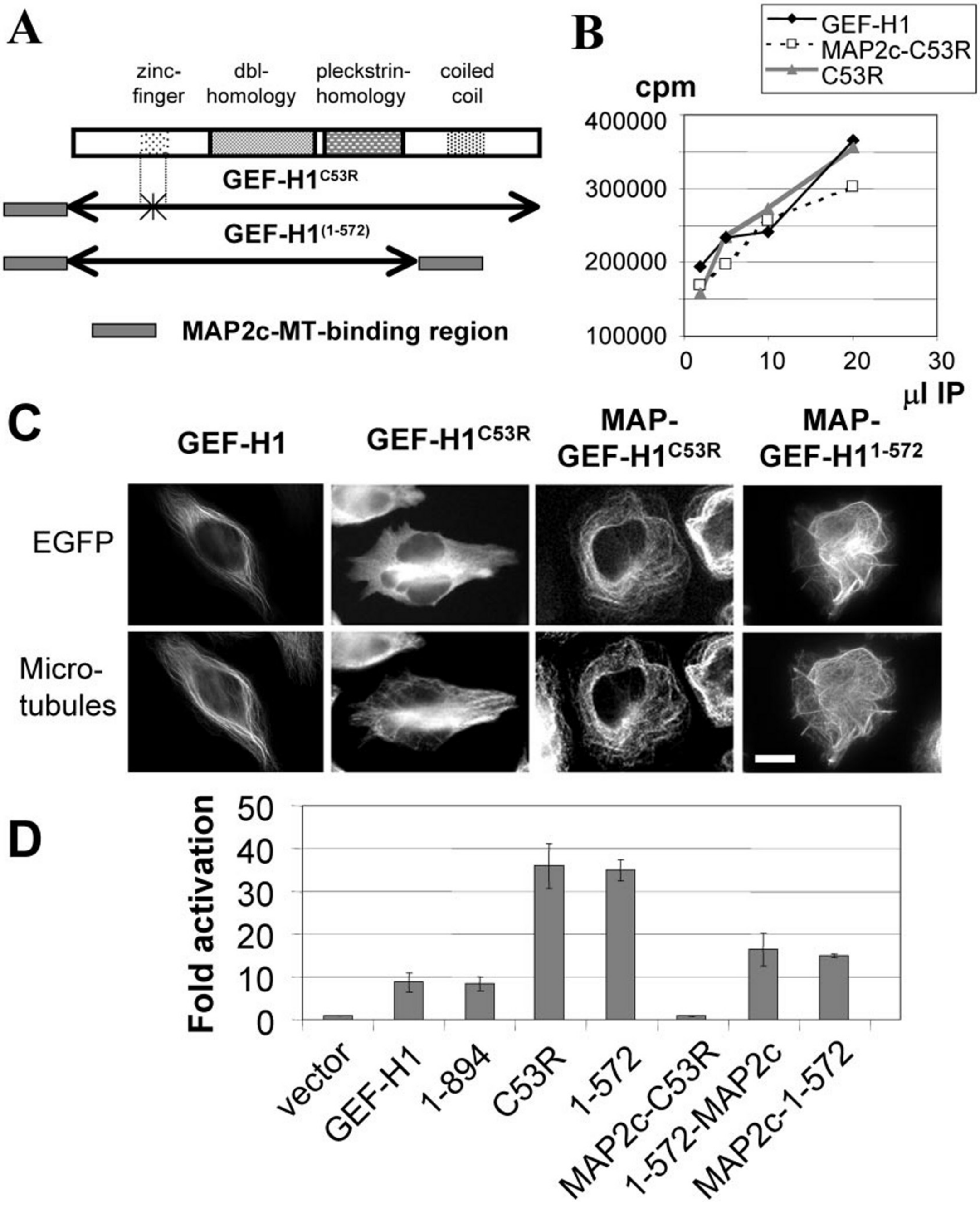

FIG. 1. **Characterization of the region important for GEF-H1 regulation by microtubule binding.** *A*, GEF-H1 constructs used in this study. Full-length GEF-H1 containing a zinc finger mutation (GEF-H1^{C53R}) or truncated versions of wild-type GEF-H1 were expressed as EGFP fusion proteins, some of which contained the MAP2c microtubule-binding region at the amino or carboxyl terminus. *B*, comparison of *in vitro* nucleotide exchange activity of GEF-H1 constructs. EGFP-tagged GEF-H1 constructs were expressed in COS-1 cells, immunoprecipitated from cell lysates, and used to catalyze binding of [^{35}S]GTPγS to recombinant RhoA (see "Experimental Procedures"). Nucleotide exchange activity of MAP2c-GEF-H1^{C53R} was similar to that of wild-type GEF-H1 and GEF-H1^{C53R}. *C*, intracellular localization of GEF-H1 constructs. HeLa cells expressing EGFP-tagged GEF-H1 constructs were fixed and stained with anti-tubulin antibodies. While GEF-H1^{C53R} was diffusely distributed in the cytoplasm, wild-type GEF-H1 and chimeric constructs containing the MAP2c microtubule-targeting domain were co-localized with microtubules. The *scale bar* represents 10 μm. *D*, comparison of *in vivo* activity of various GEF-H1 constructs. Activity of EGFP-GEF-H1 constructs expressed in COS-1 cells toward Rho was measured using the SRE-luciferase reporter gene assay and expressed as -fold activation relative to the vector-transfected control. Full-length GEF-H1 exhibited low *in vivo* activity, while non-microtubule-localized constructs GEF-H1^{C53R} and GEF-H1-(1–572) were highly active. Addition of MAP2c fragment to GEF-H1^{C53R} resulted in the complete loss of activity, while MAP2c-GEF-H1-(1–572) and GEF-H1-(1–572)-MAP2c constructs lacking the carboxyl terminus were more active. Each data point represents the average ± S.D. of at least two independent experiments. *IP*, immunoprecipitation.

and pleckstrin homology domain arrangement typical for Rho guanine nucleotide exchange factors (Fig. 1*A*). In addition to the exchange factor domain, GEF-H1 contains an amino-terminal zinc finger and a carboxyl-terminal coiled-coil domain. GEF-H1 localizes to microtubules, and this localization can be disrupted by removal or mutation of either amino- or carboxyl-terminal portions of the molecule, including the zinc finger domain. We have shown that the lack of microtubule localization of amino- or carboxyl-terminally modified GEF-H1 correlates with an increase in its activity toward Rho GTPase *in vivo*, indicating that microtubules negatively regulate GEF-H1 activity (12).

To further elucidate the mechanism of regulation of GEF-H1 activity, we attempted to restore microtubule localization of GEF-H1 mutants deficient in the ability to interact with microtubules and examine their activity *in vivo*. We have previously shown that a single amino acid substitution in the amino-terminal zinc finger domain of GEF-H1 (C53R) is sufficient to completely abolish microtubule localization. We produced an EGFP-tagged fusion protein consisting of the microtubule-binding region of MAP2c (from amino acid 231 to the end) and GEF-H1^{C53R} (Fig. 1*A*). As expected, this fusion protein co-localized with microtubules (Fig. 1*C*). Comparison of the *in vivo* activity of GEF-H1^{C53R} and MAP2c-GEF-H1^{C53R} using the SRE-luciferase reporter gene assay revealed that while GEF-H1^{C53R} was highly active, MAP2c-GEF-H1^{C53R} exhibited virtually no activity (Fig. 1*D*). Thus, restoration of microtubule localization of GEF-H1^{C53R} mutant was sufficient to fully inhibit its activity. This was not due to inhibitory effects resulting from addition of the MAP2c fragment itself since MAP2c-GEF-H1^{C53R} protein exhibited normal guanine nucleotide exchange activity toward Rho *in vitro* (Fig. 1*B*). The observation that the activity of MAP2c-GEF-H1^{C53R} toward Rho *in vivo* was slightly lower than that of GEF-H1 may reflect differences in microtubule binding sites between MAP2c and GEF-H1 and/or differences in the affinity of the two proteins for microtubules. We find it unlikely, however, that the exact reproduction of the inhibitory profile observed with untagged GEF-H1 by the MAP2c-tagged versions is a result of selective site interactions rather than microtubule localization *per se*.

After confirming that the MAP2c fragment was capable of restoring microtubule localization and inhibition of the GEF-H1^{C53R} mutant, we decided to utilize this microtubule-targeting strategy to identify regions of GEF-H1 that are important for microtubule-dependent regulation of its activity. To do so, we created fusion proteins containing a carboxyl-terminally truncated GEF-H1 construct (amino acids 1–572) fused to the MAP2c fragment at either the amino or carboxyl terminus. While these proteins appeared to localize on microtubules (Fig. 1*C*) and exhibited *in vitro* nucleotide exchange activity similar to that of full-length GEF-H1 (see Supplemental Fig. 1), they were more active *in vivo* than MAP2c-GEF-H1^{C53R} (Fig. 1*D*). To determine whether the increased *in vivo* activity of carboxyl-terminally truncated MAP2c-GEF-H1 fusion proteins could be due to an increase in the amount of protein not bound to microtubules, we performed a quantitative analysis of intracellular distribution of various GEF-H1 constructs using a technique described by Ozer and Halpain (16). This approach relies on the observation that proteins bound to cytoskeletal elements such as microtubules exhibit highly inhomogeneous distribution within a cell that is characterized by high variability in fluorescence intensity between pixels. To verify that this quantitative approach can be applied to GEF-H1, we compared the coefficient of fluorescence intensity variation for GEF-H1^{C53R} and GEF-H1-(1–572) with those for microtubule-bound proteins GEF-H1 and MAP2c-GEF-H1^{C53R} (see Table I). Coefficients of variation for GEF-H1-(1–572) and GEF-H1^{C53R}, which do not co-localize with microtubules and are diffusely distributed in the cytoplasm, were significantly lower than for the microtubule-interacting constructs (t test, $p < 0.01$). Most importantly, coefficients of variation for MAP2c-GEF-H1-(1–572), GEF-H1-(1–572)-MAP2c, and MAP2c-GEF-H1^{C53R} were not significantly different from each other (t test, $p > 0.05$), suggesting that MAP2c-GEF-H1-(1–572) and GEF-H1-(1–572)-MAP2c constructs did not exhibit more pronounced cytoplasmic localization (Table I). Thus, while MAP2c-GEF-H1 fusion proteins lacking the carboxyl-terminal amino acid residues 573–985 were co-localized with microtubules, targeting these proteins to microtubules was no longer sufficient to fully inhibit their exchange activity.

TABLE I

Quantitative analysis of intracellular localization of GEF-H1 constructs

Images of cells expressing EGFP-tagged constructs were analyzed as described under "Experimental Procedures." Fluorescence intensity variation (standard deviation of pixel intensity divided by average pixel intensity) was measured for each cell. The resulting values for each construct were averaged. At least 40 cells were analyzed for each construct.

Construct	Intensity variation (mean ± S.D.)
GEF-H1	0.26 ± 0.11
GEF-H1-(1–572)	0.16 ± 0.07
GEF-H1^{C53R}	0.18 ± 0.06
MAP2c-GEF-H1^{C53R}	0.23 ± 0.11
MAP2c-GEF-H1-(1–572)	0.23 ± 0.12
GEF-H1-(1–572)-MAP2c	0.27 ± 0.11

Identification of GEF-H1 as a Substrate of PAK1—In an independent screen to isolate new substrates of p21-activated kinases, we produced a recombinant protein consisting of glutathione *S*-transferase fused to the PAK1 kinase domain (amino acids 233–544) and used this protein bait to affinity-purify binding proteins from total cell lysates. To differentiate between mere binding partners and substrate proteins, we also performed *in vitro* kinase assays with the bound proteins. Applying this affinity reagent to Jurkat cell lysates, we observed that a prominent band of ~110 kDa (p110) was present in the purified fractions (Fig. 2*A*, *lower panel*, *lanes 3* and *4*). In binding reactions using GST alone (Fig. 2*A*, *lanes 1* and *2*) or a catalytically inactive version of PAK1 (PAK1^{D389A}), p110 was not bound. Using the affinity-purified complex isolated from Jurkat lysates for an *in vitro* kinase assay, we observed that p110 was strongly phosphorylated. Phosphorylation was PAK1-dependent since the presence of the PAK-specific autoinhibitory domain (amino acids 83–149 of human PAK1) markedly reduced incorporation of radiolabeled phosphate (Fig. 2*A*, *lane 3 versus lane 4*). Interestingly the PAK-inhibitory peptide not only inhibited phosphorylation but also interaction with the GST-PAK1 fusion when added to the initial binding reactions (data not shown). The reduction in phosphorylation and binding were both dependent on an intact autoinhibitory domain since an inhibitory-deficient L107F mutant peptide (20) was inactive.

We determined a peptide mass fingerprint from a tryptic digest of gel-purified p110 using MALDI-TOF mass spectroscopy and searched a protein data base using the program ProFound (17). Nine of 17 peptide masses matched fragments from the human protein termed GEF-H1/KIAA-0651. We could confirm the identification in two more independent experiments using the peptide mass fingerprint approach (10 of 20 and 10 of 19 peptide masses matching, respectively). The predicted molecular weight of the GenBank™ entry for KIAA-0651 (GenBank™ accession number gi3327116) was 103 kDa, which is in good agreement with the apparent molecular weight of the affinity-purified protein estimated by SDS-gel electrophoresis. BLAST sequence comparison of the genomic locus of KIAA-0651/GEF-H1 (GenBank™ accession number gi11427616) with the corresponding cDNAs suggested that KIAA-0651 and GEF-H1 represent alternative splice isoforms. Sequence differences are only due to alternative use of a starting exon; all other exons in both isoforms are identical.

Antibodies raised against GEF-H1 (11) were used to confirm that p110 was indeed GEF-H1. The GEF-H1 antibodies recognized a 110-kDa band in Jurkat lysates and strongly reacted with the p110 protein in purified fractions. We additionally generated a polyclonal rabbit antiserum against amino acids

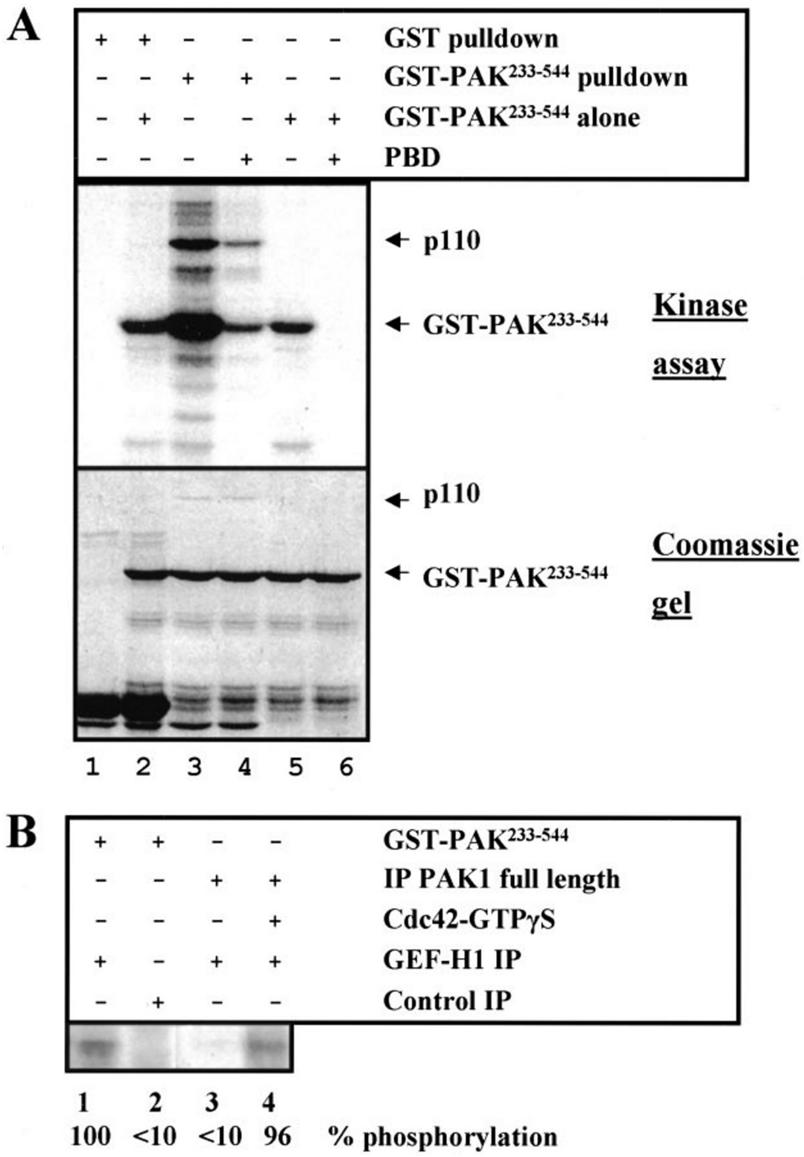

FIG. 2. **GST-PAK binds and phosphorylates a 110-kDa protein from Jurkat cell lysates.** *A*, lysates from Jurkat T cells were incubated with immobilized GST-PAK1-(233–544) for 1 h at 4 °C (*lane 3* and *4*). As a control, immobilized GST was used for binding reactions (*lanes 1* and *2*). Binding reactions were washed extensively and incubated in the presence of radiolabeled ATP (see "Experimental Procedures") to allow phosphorylation of bound proteins by PAK. The PAK autoinhibitory peptide (*PBD*, amino acids 67–150) was added at a final concentration of 4 μM as indicated. Purified GST-PAK1-(233–544) protein was added to the GST pull-down reaction (*lane 2*) to ensure that no 110-kDa protein was phosphorylated unspecifically. An autoradiogram is shown in the *upper panel*. The corresponding Coomassie gel is shown in the *lower panel*. Positions of the GST-PAK-(233–544) and p110 proteins are indicated by *arrows*. *B*, equivalent phosphorylation of GEF-H1 by full-length and active carboxyl-terminal PAK1. GEF-H1 was immunoprecipitated from Jurkat cell lysates, and equivalent amounts of precipitated GEF-H1 (*lanes 1*, *3*, and *4*) or control immunoprecipitate (*lane 2*) were utilized in an *in vitro* kinase assay with GST-PAK1-(233–544) (*lanes 1* and *2*) or full-length PAK1 (*lanes 3* and *4*). Results of the quantitation of the relative levels of phosphorylation are shown *under* each lane. The level of control phosphorylation using MBP as substrate was similar among *lanes 1*, *2* and *4* (not shown). *IP*, immunoprecipitation.

614–803 of GEF-H1 fused to glutathione *S*-transferase, affinity-purified the antiserum, and showed that p110 cross-reacted with our GEF-H1-specific antiserum as well. Epitope-tagged GEF-H1 and KIAA-0651 from COS-1 cell lysates also specifically interacted with GST-PAK1-(233–544), and the ectopically expressed GEF-H1 or KIAA-0651 were also phosphorylated to similar extents in *in vitro* kinase assays with either GST-PAK1-(233–544) or with Cdc42-activated full-length PAK1 (Fig. 2*B*). Taken together, these data confirmed that the isolated protein from Jurkat cell lysates is indeed GEF-H1/KIAA-0651. However, we did not further determine which isoform was purified from the Jurkat cells.

Identification of the PAK1 Phosphorylation Sites on GEF-H1—Full-length GEF-H1 was co-expressed with full-length $PAK1^{T423E,L107F}$ constitutively active mutant in $^{32}PO_4$ metabolically labeled HeLa cells, and the immunoprecipitated GEF-H1 was analyzed by two-dimensional phosphopeptide mapping. As seen in Fig. 3*A*, GEF-H1 exhibited multiple spots in the two-dimensional separation, indicating that PAK1 induced phosphorylation of GEF-H1 on multiple sites *in vivo*.

To identify sites of phosphorylation, we attempted an *in vitro* analysis of GEF-H1 phosphorylation by PAK1. Various regions of GEF-H1 were generated as glutathione *S*-transferase fusion proteins in *Escherichia coli* and used as substrates in *in vitro* kinase assays as described (Fig. 3*B*). Constitutively active GST-PAK1 only phosphorylated the fusion protein containing the carboxyl-terminal amino acids 572–985 of GEF-H1. Further fine mapping of the carboxyl-terminal 572–985 region showed that carboxyl-terminal deletions down to amino acid 912 were tolerated, but further deletion abolished phosphate incorporation. Phosphoamino acid analysis indicated that only serine was phosphorylated by GST-PAK1 (not shown). In addition, only one radiolabeled spot with low electrophoretic, but high chromatographic, mobility appeared in a tryptic phosphopeptide map of this fragment (not shown). These results, when combined with sequence analysis, suggested Ser^{885} (per GEF-H1 numbering) as a candidate phosphorylation site. In confirmation of this, mutation of Ser^{885} to alanine totally abolished phosphate incorporation into GST-GEF-H1-(572–985) (Fig. 3*C*) and resulted in the loss of the single phosphopeptide spot observed on two-dimensional maps of GEF-H1 (572–985) (not shown). While Ser^{885} was present in GEF-H1 fragment containing amino acids 572–894, this fragment was not phosphorylated by PAK (Fig. 3*B*), possibly due to the removal of amino acids involved in substrate recognition or disruption of the secondary structure since this fragment terminates near the phosphorylation site. We verified that Ser^{885} was an actual PAK1 phosphorylation site in intact GEF-H1 *in vivo* by phos-

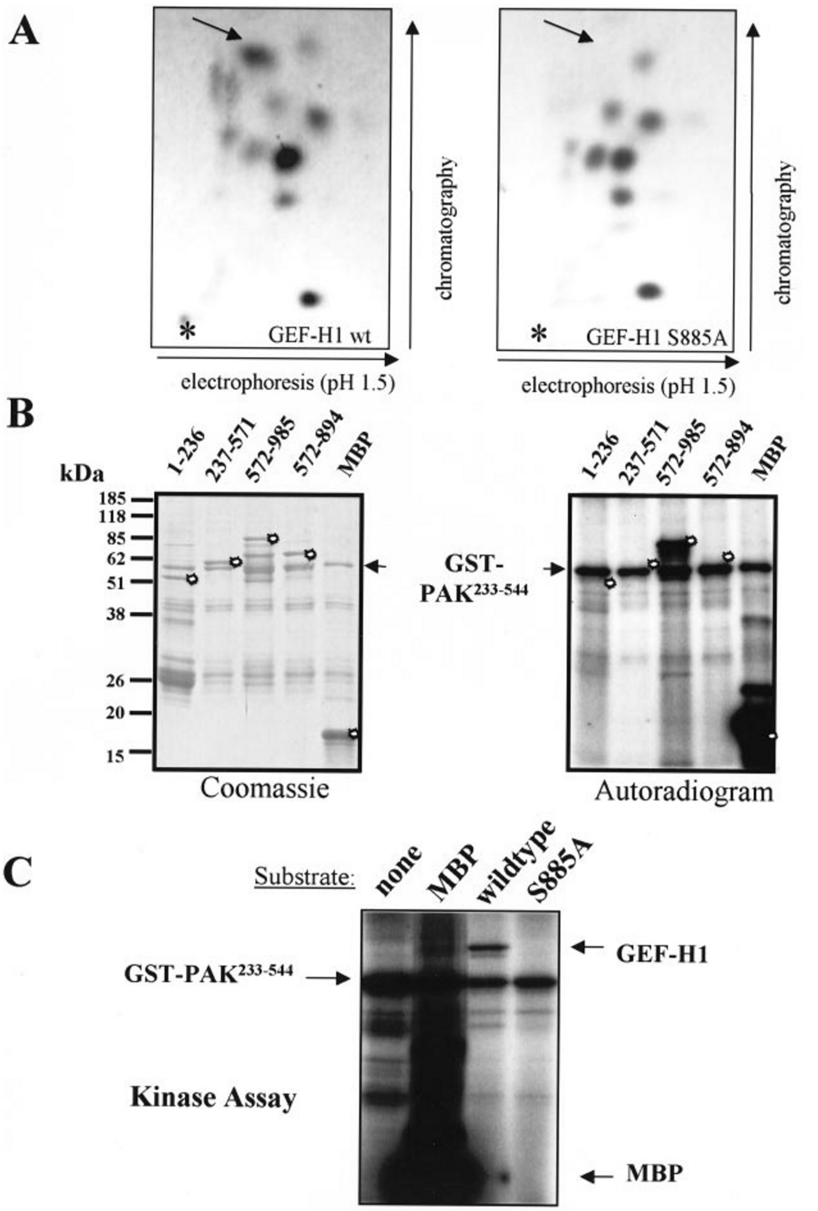

FIG. 3. **Mapping of the PAK phosphorylation site(s) on GEF-H1.** *A*, full-length wild-type (*wt*) EGFP-GEF-H1 or EGFP-GEF-H1^{S885A} was co-expressed in $^{32}PO_4$ metabolically labeled HeLa cells with constitutively active PAK1^{T423E,L107F}. Expressed GEF-H1 was immunoprecipitated with EGFP antibody, and the precipitated protein was subjected to two-dimensional phosphopeptide analysis as described under "Experimental Procedures." The absence of the Ser^{885} spot is indicated by the *arrow*. Starting points of electrophoresis are indicated by *solid asterisks*. *B*, GST-PAK1-(233–544) was mixed with recombinant GST fusion proteins containing the indicated regions of the GEF-H1 protein and subjected to *in vitro* kinase reaction (see "Experimental Procedures"). The Coomassie gel and a corresponding autoradiography exposure are shown. Myelin basic protein (*MBP*) was used as a control substrate. The positions of the GEF-H1 fusion proteins and myelin basic protein on the gel are indicated by the *open asterisks*. Ser^{885} was mutated to alanine, and the corresponding GST-GEF-H1-(572–985) protein was subjected to *in vitro* phosphorylation. *C*, GST-PAK1-(233–544) kinase reactions without a substrate or with the indicated substrate proteins (*MBP*, myelin basic protein; *wild type*, GST-GEF-H1-(572–985); *S885A*, GST-GEF-H1^{S885A}-(572–985)) are shown. Wild-type and S885A mutated GST-GEF-H1-(572–985) were loaded in equivalent amounts.

phopeptide analysis of GEF-H1^{S885A} in metabolically labeled cells (Fig. 3*A*). We observed that the loss of serine at position 885 resulted in the clear loss of one of the phosphopeptide spots present in the wild-type GEF-H1. We have not identified the other phosphorylation sites observed *in vivo* at this time.

To determine whether phosphorylation of GEF-H1 at Ser^{885} by PAK1 had any effect on GEF-H1 localization or activity, we analyzed the GEF-H1^{S885A} mutant. GEF-H1^{S885A} localized normally to microtubules when expressed in HeLa cells, and its *in vivo* GEF activity measured using the SRE-luciferase assay was similar to that of wild-type GEF-H1. Conversely expression of active versions of PAK1 also did not change the binding of wild-type GEF-H1 to microtubules (data not shown). Similarly the Rho GEF activity of GEF-H1 in *in vitro* exchange assays was not altered when the protein was co-expressed with constitutively active PAK1^{T423E}. Expression of active PAK1 alone had an inhibitory effect on SRE-luciferase expression, therefore we were unable to use the SRE-luciferase assay to measure *in vivo* activity of GEF-H1 in cells co-transfected with active PAK1. However, examination of the overall morphology of HeLa cells (which is indicative of the GEF-H1 activation status, see Ref. 12) transfected with GEF-H1 and PAK1^{T423E} also indicated that phosphorylation did not cause an increase in the activity of wild-type GEF-H1 toward Rho.

Phosphorylation-dependent Binding of 14-3-3 to GEF-H1—Sequence analysis of the GEF-H1/KIAA-0651 protein using the program ScanSite (21) suggested a potential binding site for 14-3-3 proteins in the region centered around the PAK1 Ser^{885} phosphorylation site. Furthermore this area was proline-rich and was also predicted to contain a potential SH3 domain binding motif (Fig. 4*A*). Thus, we hypothesized that PAK1-mediated phosphorylation of GEF-H1 might influence the binding of other (regulatory) proteins to this region.

We tested the prediction that GEF-H1 phosphorylation by PAK1 regulates binding of 14-3-3 proteins using *in vitro* phosphorylated GST-GEF-H1-(572–985). The glutathione-agarose-bound GST-GEF-H1-(572–985) protein was incubated with bovine brain lysate, and the bead-bound fraction was analyzed by Western blotting with a generic 14-3-3-specific antiserum (Fig. 4*B*). We detected 14-3-3 in purified fractions containing phosphorylated GST-GEF-H1-(572–985). No 14-3-3 protein was present in the purified fraction with non-phosphorylated GEF-H1 or the phosphorylation-defective mutant GST-H1^{S885A} (amino acids 572–985). Thus, 14-3-3 binds to GEF-H1 in a

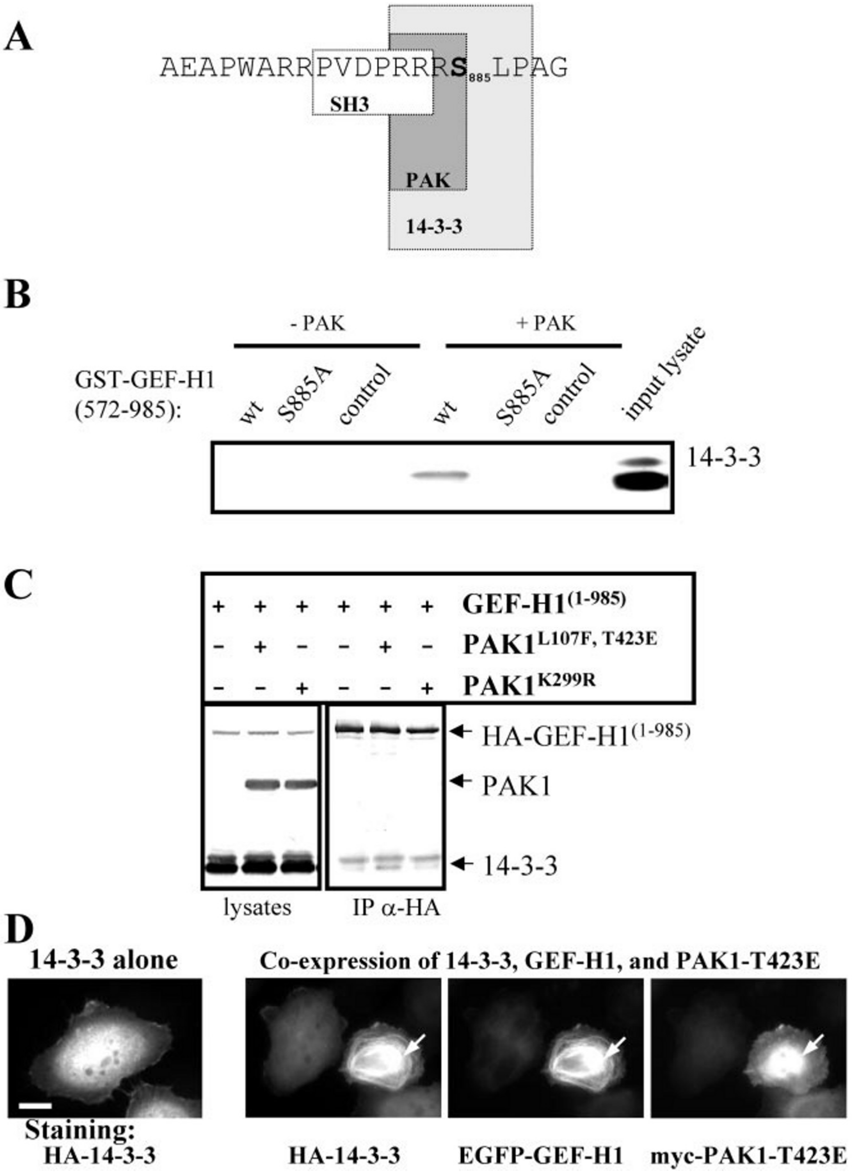

FIG. 4. **Interaction of phosphorylated GEF-H1 with 14-3-3 proteins.** *A*, amino acid sequence (amino acids 870–889) of GEF-H1 surrounding the GEF-H1 phosphorylation site. Using the protein motif search software ScanSite (21) we identified a potential SH3 binding and 14-3-3 binding motif in close vicinity of the experimentally determined GEF-H1 PAK phosphorylation site. *B*, GST fusion proteins containing amino acids 572–985 of GEF-H1 (wild type (*wt*)) and the corresponding Ser^{885} to alanine mutant (*S885A*) were immobilized on glutathione-agarose beads. Glutathione *S*-transferase bound to beads was used as a control. Equal amounts of GST proteins were used in each binding reaction (not shown). The immobilized proteins were either left untreated (*lanes 1–3*) or phosphorylated in the presence of GST-PAK1 and ATP. Samples were washed and incubated with equal amounts of bovine brain lysate. Binding reactions were washed, separated by SDS-polyacrylamide gel electrophoresis, and processed for Western blotting using polyclonal anti-14-3-3 antiserum (Santa Cruz Biotechnology). In *lane 7* bovine brain cytosol was loaded as a control. *C*, COS-1 cells were transfected with HA-tagged GEF-H1 wild-type protein and an active variant of PAK1 (*PAK1^{L107F,T423E}*) as indicated. The *first three lanes* show total amounts of ectopically expressed GEF-H1, PAK1^{L107F,T423E}, and endogenous 14-3-3 protein in cell lysates. In the *next three lanes*, protein complexes immunoprecipitated using anti-HA antibody were analyzed for their 14-3-3 content. *D*, intracellular localization of 14-3-3 in the absence and presence of PAK1-phosphorylated GEF-H1. HA-tagged 14-3-3 ζ was expressed in HeLa cells either alone (*left panel*) or together with EGFP-GEF-H1 and constitutively active Myc-PAK1^{T423E} (*remaining panels*). In the absence of GEF-H1, HA-14-3-3 ζ was diffusely distributed in the cytoplasm, while in cells expressing GEF-H1 and active PAK (*arrow*) we observed co-localization of 14-3-3 ζ with GEF-H1 on microtubules. The *scale bar* represents 10 μm. *IP*, immunoprecipitation.

PAK1 phosphorylation-dependent manner and most likely to the conserved site (RS*X*pS*X*P where pS represents phosphoserine) encompassing residues 882–887 (RRRS^{885}LP).

Co-immunoprecipitation of GEF-H1 and 14-3-3 was also observed in lysates from COS-1 cells transfected with GEF-H1. Endogenous 14-3-3 bound to immunoprecipitated GEF-H1 only when an active form, but not an inactive form, of PAK1 was co-transfected (Fig. 4*C*). Additionally HA-tagged 14-3-3 (isoforms ϵ and ζ) co-immunoprecipitated with GEF-H1 from COS-1 cells expressing constitutively active, but not kinase-dead, PAK1 (data not shown). When HA-tagged 14-3-3 (ϵ or ζ) was expressed in HeLa cells, it was diffusely distributed throughout the cell (Fig. 4*D*). However, when 14-3-3 was co-transfected with GEF-H1, in a substantial number of cells 14-3-3 was observed to co-localize with GEF-H1 on microtubules. This co-localization was particularly pronounced in cells co-expressing 14-3-3 and GEF-H1 together with constitutively active PAK1^{T423E} (Fig. 4*D*). 80% of cells expressing 14-3-3, GEF-H1, and PAK1^{T423E} exhibited microtubule localization of 14-3-3, while only 25% of cells expressing 14-3-3, GEF-H1, and kinase-dead PAK1^{K299R} exhibited co-localization of 14-3-3 with microtubules (n = 100 cells for each experimental condition). These observations suggest that phosphorylation-dependent binding of 14-3-3 to GEF-H1 promotes relocation of 14-3-3 to microtubules. The co-immunoprecipitation of GEF-H1 and 14-3-3 together with the PAK1- and GEF-H1-dependent relocalization of 14-3-3 also indicate that GEF-H1 is phosphorylated by PAK1 *in vivo* at Ser^{885}.

The PAK1- and GEF-H1-dependent relocalization of 14-3-3 to microtubules suggested the possibility that 14-3-3 binding might be responsible for the suppression of microtubule-associated GEF-H1 Rho exchange activity. However, when GEF-H1 was immunoprecipitated from COS-1 cells co-transfected with active PAK1 and used to catalyze nucleotide exchange on Rho, no suppression of intrinsic exchange activity was observed although bound 14-3-3 was present in GEF-H1 immunoprecipitates (data not shown). While this observation suggests lack of an effect of 14-3-3 binding on GEF-H1 activity, the possibility of regulation of GEF-H1 exchange activity by 14-3-3 cannot be conclusively ruled out since the amount of 14-3-3 in GEF-H1 immunoprecipitates may be substoichiometric.

DISCUSSION

We previously reported that the guanine nucleotide exchange activity of GEF-H1 toward Rho was inhibited by interaction of GEF-H1 with microtubules (12). In the present study, we con-

firmed the role of microtubule localization in regulation of activity of GEF-H1 by creating a chimeric protein that consists of the microtubule-binding region of MAP2c (13) and a form of GEF-H1 (GEF-H1^{C53R}) containing a mutation in the zinc finger domain. While GEF-H1^{C53R} did not localize to microtubules and was highly active toward Rho (12), MAP2c-GEF-H1^{C53R} was localized on microtubules and showed no activity toward Rho *in vivo*. This result confirmed that association with microtubules leads to inhibition of exchange activity of GEF-H1. Of particular interest, we determined that removal of the carboxyl terminus (amino acids 573–985) from the MAP2c-GEF-H1 fusion protein resulted in up-regulation of GEF activity although the truncated, MAP2c-tagged proteins (MAP2c-GEF-H1-(1–572) and GEF-H1-(1–572)-MAP2c) were still localized on microtubules. These data suggest that the carboxyl terminus of GEF-H1 contains a region that is necessary for suppression of GEF-H1 exchange activity, possibly through interaction with tubulin and/or other proteins associated with microtubules. Previously we found that the GEF-H1 construct consisting of amino acids 1–894 was localized on microtubules and had low *in vivo* GEF activity similar to full-length GEF-H1. Therefore, the region involved in regulation of GEF-H1 activity can be narrowed down to amino acids 573–894 (and our preliminary studies suggest that it can be further narrowed down to amino acids 732–894). Of interest, this carboxyl-terminal portion of GEF-H1 contains several regions that may be involved in regulatory protein-protein interactions, including a coiled-coil domain, a P*XX*P motif that may bind to SH3 domain-containing proteins, and a 14-3-3 protein binding motif.

Through an independent biochemical screen for novel substrates of PAK1, we also identified GEF-H1 as a PAK1 substrate. Two-dimensional phosphopeptide mapping indicated that the co-expression of active PAK1 with GEF-H1 in HeLa cells resulted in phosphorylation of GEF-H1 at multiple sites (Fig. 3*A*). *In vitro* analysis with recombinant GEF-H1 fragments showed phosphorylation of a single site, Ser^{885}, in the fragment 572–985. While we verified that the Ser^{885} site in GEF-H1 was phosphorylated by full-length PAK1 *in vivo* (Fig. 3*A*), there clearly exist other sites of phosphorylation. The lack of phosphorylation of additional GEF-H1 fragments in our biochemical analyses might be due to improper folding and conformation of the expressed fragments. Alternatively the phosphorylation at additional sites in HeLa cells overexpressing PAK1^{T423E,L107F} might be due to the activation by PAK1 of additional kinases that utilize GEF-H1 as a substrate.

The Ser^{885} phosphorylation site lies within the region (amino acids 573–894) that may be involved in inhibitory modulation of GEF-H1 activity. Interestingly Ser^{885} is embedded in the GEF-H1 consensus binding site for 14-3-3 protein (Fig. 4*A*). The binding of 14-3-3 proteins to their targets typically requires phosphorylation of serine in the binding site (22). Indeed we have demonstrated that 14-3-3 binds to GEF-H1 in a phosphorylation-dependent manner and that a portion of 14-3-3 translocates from the cytoplasm to the microtubules upon binding to phosphorylated GEF-H1. Thus, phosphorylation of Ser^{885} by PAK induces binding of 14-3-3 to GEF-H1. 14-3-3 proteins are regulatory proteins that bind to a variety of cellular targets, including the pro-apoptotic protein Bad, Raf kinase, cell cycle-dependent phosphatase Cdc25, and others (22). 14-3-3 binding regulates the subcellular localization and activity of these proteins and their ability to interact with other components of intracellular signaling pathways. For example, the interaction of 14-3-3 with Cdc25 may prevent it from relocating to the nucleus where it is involved in regulation of cell cycle progression. Similarly binding of 14-3-3 to Bad sequesters Bad in the cytosol so that it cannot interact with antiapoptotic proteins Bcl-2 and Bcl-X_L in mitochondria. In our experiments we did not observe any effect of 14-3-3 binding on microtubule localization of GEF-H1. Instead we observed that a fraction of 14-3-3 translocated from the cytosol to microtubules upon PAK1 phosphorylation-dependent binding to GEF-H1. Interestingly 14-3-3 protein (isoform ζ) has also been shown to interact with the neuronal microtubule-associated protein tau and to co-purify with microtubules from brain extract (23). 14-3-3 and tubulin share a common binding site on tau, and their binding to tau is mutually exclusive (23), suggesting that 14-3-3 may regulate binding of tau to microtubules. 14-3-3 also modulates phosphorylation of tau by protein kinase A (23). 14-3-3 isoforms β, γ, ϵ, and η have been shown to interact with the Rho guanine nucleotide exchange factor p190RhoGEF (24), which is enriched in neurons and also associates with microtubules. The binding of 14-3-3 to p190RhoGEF regulates formation of cytoplasmic aggregates of this exchange factor, although the physiological significance of this observation is unclear. Thus, in neurons several isoforms of 14-3-3 may be involved in regulation of localization and activity of proteins that are normally associated with microtubules.

In our experiments, we observed that 14-3-3 expressed in non-neuronal (HeLa) cells was not localized to microtubules in the absence of phosphorylated GEF-H1 but was recruited to microtubules upon binding to PAK1-phosphorylated GEF-H1. Binding to GEF-H1 and recruitment to microtubules was observed for 14-3-3 isoforms ϵ and ζ, suggesting that both of these isoforms may interact with GEF-H1 in a phosphorylation-dependent fashion. Whether other isoforms of 14-3-3 can also interact with GEF-H1 remains to be determined. We hypothesized that, similar to tau and p190RhoGEF, localization and/or function of GEF-H1 may be modulated as a result of 14-3-3 binding. However, PAK1-mediated phosphorylation of GEF-H1 and/or 14-3-3 binding did not have a discernible effect on GEF-H1 nucleotide exchange activity *in vitro* or *in vivo*. Since microtubule localization and exchange activity of GEF-H1 were unaffected by 14-3-3 binding, we suggest that it is likely that 14-3-3 binding regulates the interaction of GEF-H1 with yet unidentified binding partners. For example, binding of SH3 domain-containing proteins to the proline-rich P*XX*P motif located adjacent to the 14-3-3 binding site may be regulated by 14-3-3. Since the 14-3-3 binding site and the proline-rich motif are located within the region that we found to be important for inhibitory regulation of GEF-H1 activity, it is possible that an interplay between 14-3-3 and an as yet unidentified SH3 domain-containing protein may be important for modulating GEF activity. Our preliminary experiments directed toward identification of the role of the proline-rich motif in regulation of GEF-H1 functions indicate that proline to alanine substitutions in the P*XX*P motif do not affect GEF-H1 localization or activity in HeLa or COS-1 cells. However, it is conceivable that regulation of GEF-H1 activity is cell type-specific and that SH3 domain-containing proteins that bind to GEF-H1 may be present only in a particular cell type, for example, in leukocytes or neurons where GEF-H1 is highly expressed (11). Alternatively PAK1-mediated phosphorylation of GEF-H1 may be involved in regulating GEF-H1 activity in a cell cycle-dependent manner. This possibility is particularly attractive since it has recently been shown that PAK undergoes phosphorylation by cell cycle-dependent kinase Cdc2 and that this phosphorylation modulates postmitotic spreading of fibroblasts (25, 26). Phosphorylation of PAK1 at the same Thr^{212} site is also mediated by the neuronal p35/Cdk5 kinase (27). Of particular interest, phosphorylation of PAK1 at this site induces co-localization of PAK1 with microtubules, and expression of a PAK1^{T212A} mu-

tant induces abnormal microtubular morphology (25). A role for the *Schizosaccharomyces pombe* p21-activated kinase Shk1 in the regulation of microtubule dynamics has been described (28). Further investigation is needed to identify additional components of this regulatory system and their relationship to GEF-H1 function.

It is of interest to note that the interaction of PAK1 with another guanine nucleotide exchange factor, PIX/Cool, has been reported previously (29, 30). PIX is a regulator of guanine nucleotide exchange on Rac and/or Cdc42, which modulates PAK activity (31). Our finding that PAK1, a downstream effector of Rac and Cdc42, binds and phosphorylates a second GEF, GEF-H1, an activator of Rho, suggests a possibility of a cross-talk between signaling pathways mediated by these GTPases. Rho, Rac, and Cdc42 are involved in regulation of certain common aspects of cell physiology. All of these GTPases regulate actin polymerization and organization of the actin cytoskeleton, modulate organization and dynamics of cell-cell and cell-substrate adhesions, and participate in regulation of cell growth and differentiation. In some cases effects of Rho and Rac are antagonistic. For example, activated Rac promotes cell spreading and reduces the number of actin stress fibers, while activated Rho increases cell contractility and enhances stress fiber formation (32). In other cases Rac and Rho are involved in different stages of the same process. One such case is during establishment of cell-substrate contacts where Rac induces assembly of small nascent adhesions, while Rho promotes maturation of these adhesion complexes into larger focal contacts (33). Phosphorylation of GEF-H1 by PAK may serve as a means to coordinate activities of Rac/Cdc42 and Rho through a positive or negative feedback mechanism.

REFERENCES

1. Condeelis, J. (1993) *Annu. Rev. Cell Biol.* **9,** 411–444
2. Small, J. V., Stradal, T., Vignal, E., and Rottner, K. (2002) *Trends Cell Biol.* **12,** 112–120
3. Hall, A. (1998) *Science* **279,** 509–514
4. Ridley, A. J. (2001) *J. Cell Sci.* **114,** 2713–2722
5. Liao, G., Nagasaki, T., and Gundersen, G. G. (1995) *J. Cell Sci.* **108,** 3473–3483
6. Wittmann, T., and Waterman-Storer, C. M. (2001) *J. Cell Sci.* **114,** 3795–3803
7. Waterman-Storer, C. M., Worthylake, R. A., Liu, B. P., Burridge, K., and Salmon, E. D. (1999) *Nat. Cell Biol.* **1,** 45–50
8. Wittmann, T., Bokoch, G. M., and Waterman-Storer, C. M. (2004) *J. Biol. Chem.* **279,** 6196–6203
9. Enomoto, T. (1996) *Cell Struct. Funct.* **21,** 317–326
10. Liu, B. P., Chrzanowska-Wodnicka, M., and Burridge, K. (1998) *Cell Adhes. Commun.* **5,** 249–255
11. Ren, Y., Li, R., Zheng, Y., and Busch, H. (1998) *J. Biol. Chem.* **273,** 34954–34960
12. Krendel, M., Zenke, F. T., and Bokoch, G. M. (2002) *Nat. Cell Biol.* **4,** 294–301
13. Ferralli, J., Doll, T., and Matus, A. (1994) *J. Cell Sci.* **107,** 3115–3125
14. Knaus, U. G., and Bokoch, G. M. (1998) *Int. J. Biochem. Cell Biol.* **30,** 857–862
15. Daniels, R. H., and Bokoch, G. M. (1999) *Trends Biochem. Sci.* **24,** 350–355
16. Ozer, R. S., and Halpain, S. (2000) *Mol. Biol. Cell* **11,** 3573–3587
17. Zhang, W., and Chait, B. T. (2000) *Anal. Chem.* **72,** 2482–2489
18. Boyle, W. J., van der Geer, P., and Hunter, T. (1991) *Methods Enzymol.* **201,** 110–149
19. Downward, J. (1995) *Methods Enzymol.* **255,** 110–117
20. Zenke, F. T., King, C. C., Bohl, B. P., and Bokoch, G. M. (1999) *J. Biol. Chem.* **274,** 32565–32573
21. Yaffe, M. B., Leparc, G. G., Lai, J., Obata, T., Volinia, S., and Cantley, L. C. (2001) *Nat. Biotechnol.* **19,** 348–353
22. Fu, H., Subramanian, R. R., and Masters, S. C. (2000) *Annu. Rev. Pharmacol. Toxicol.* **40,** 617–647
23. Hashiguchi, M., Sobue, K., and Paudel, H. K. (2000) *J. Biol. Chem.* **275,** 25247–25254
24. Zhai, J., Lin, H., Shamim, M., Schlaepfer, W. W., and Canete-Soler, R. (2001) *J. Biol. Chem.* **276,** 41318–41324
25. Banerjee, M., Worth, D., Prowse, D., and Nikolic, M. (2002) *Curr. Biol.* **12,** 1233–1239
26. Thiel, D., Reeder, M., Pfaff, A., Coleman, T., Sells, M., and Chernoff, J. (2002) *Curr. Biol.* **12,** 1227–1232
27. Rashid, T., Banerjee, M., and Nikolic, M. (2001) *J. Biol. Chem.* **276,** 49043–49052
28. Qyang, Y., Yang, P., Du, H., Lai, H., Kim, H., and Marcus, S. (2002) *Mol. Microbiol.* **44,** 325–334
29. Bagrodia, S., Taylor, S. J., Jordon, K. A., Van Aelst, L., and Cerione, R. A. (1998) *J. Biol. Chem.* **273,** 23633–23636
30. Manser, E., Loo, T. H., Koh, C. G., Zhao, Z. S., Chen, X. Q., Tan, L., Tan, I., Leung, T., and Lim, L. (1998) *Mol. Cell* **1,** 183–192
31. Daniels, R. H., Zenke, F. T., and Bokoch, G. M. (1999) *J. Biol. Chem.* **274,** 6047–6050
32. Aspenstrom, P. (1999) *Exp. Cell Res.* **246,** 20–25
33. Rottner, K., Hall, A., and Small, J. V. (1999) *Curr. Biol.* **9,** 640–648
