# Supplementary material for: CD20 tails interact with the 14-3-3/GEF-H1 complex and microtubule network upon PKCδ phosphorylation
Source: EMBO J. 2026 Apr 17;45(11):3859–79. doi: 10.1038/s44318-026-00781-5 (PMC13226681; doi:10.1038/s44318-026-00781-5)

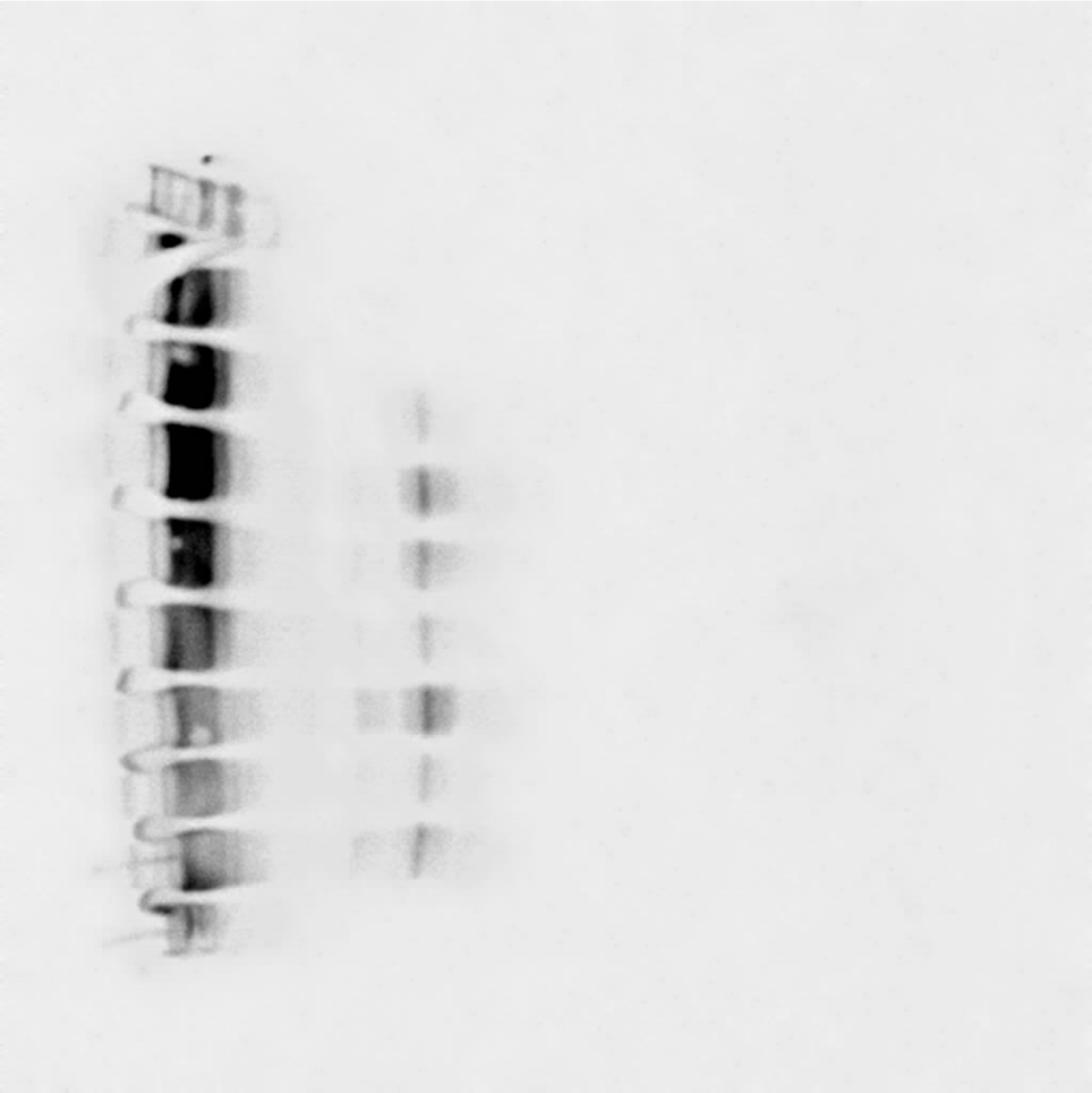

Supplement: Supplementary file 8 — Source data Fig. 4 [file 44318_2026_781_MOESM8_ESM.zip › Figure 4/E/Images WB.zip!/Images WB/Fig 4E active GEF-H1.pdf]
